# LiMA: Robust inference of molecular mediation from summary statistics

**DOI:** 10.1016/j.ajhg.2025.12.005

**Published:** 2026-01-08

**Authors:** Kaido Lepik, Chiara Auwerx, Marie C. Sadler, Adriaan van der Graaf, Sven Erik Ojavee, Zoltán Kutalik

**Affiliations:** 1 University Center for Primary Care and Public Health, Lausanne, Switzerland; 2 Swiss Institute of Bioinformatics, Lausanne, Switzerland; 3 Department of Computational Biology, University of Lausanne, Lausanne, Switzerland

**Keywords:** causality, genetic epidemiology, mediation, omics, statistical genetics, Mendelian randomization

## Abstract

Understanding the molecular mechanisms mediating the causal effects of epidemiological risk factors on complex traits can advance targeted disease interventions. Statistical mediation analysis facilitates this by disentangling direct and indirect causal effects. Current approaches to causal mediation leverage Mendelian randomization, using summary statistics from the exposure, mediator, and outcome studies that estimate the genetic effects of instruments. However, differences in study sample sizes (measurement errors) lead to substantial biases and poorly controlled type I error rates for these methods, which become especially pronounced when simultaneously estimating the mediation proportion of numerous mediators. To address these limitations, we introduce Likelihood-based Mediation Analysis (LiMA), which estimates molecular mediation more accurately and robustly by jointly modeling the variability in all estimates involved. Through extensive simulation studies and benchmarking, we demonstrate that our approach achieves several-fold lower bias and improved control for type I error than state-of-the-art methods. Applying our method to real data highlighted several plausible metabolites—such as glutamate and carnitine—as well as proteins mediating the causal effects of obesity-related risk factors on cardiometabolic outcomes. These findings underscore the potential of our framework to reveal promising molecular pathways underlying complex diseases. By accommodating the variability inherent to summary statistics of varying precision, LiMA enables robust mediation analyses across large sets of mediators.

## Teaser

LiMA and its random-effect variant I-LiMA infer molecular mediation in a Mendelian randomization framework using summary statistics. Jointly modeling direct and mediated effects while accounting for measurement error, they reduce weak-instrument bias and false positives in simulations. Applications to real data reveal metabolites and proteins mediating obesity-related cardiometabolic risk.

## Introduction

Applications of causal inference on large-scale observational data have enabled the discovery of causal links between a wide range of complex traits and diseases.^1^ These have greatly improved our understanding of the intermediate steps from genetic variation to disease outcomes. Mendelian randomization (MR) has become a standard approach for such causal inquiries due to its robustness against confounding and reverse causation. Its applicability to summary statistics, combined with the sharp increase in study sample sizes and the widespread availability of summary data from genome-wide association studies (GWASs) for both complex^2^^,^^3^ and molecular outcomes,^4^^,^^5^^,^^6^^,^^7^ opens up ever-increasing possibilities for insightful applications.

**Figure 1:**
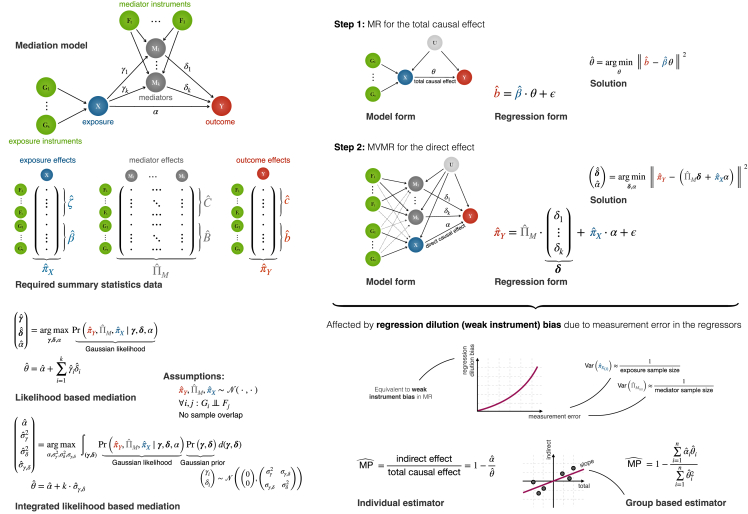
Framework for the mediation analysis (A) Mediation model where the total causal effect from an exposure to an outcome has been decomposed into a direct effect α and indirect effects *γ*_*i*_*δ*_*i*_ via the mediators. Summary statistics of genetic instrument effects on the exposure, mediators, and outcome are used to estimate α and *γ*_*i*_*δ*_*i*_. (B) Two-step procedure to estimate the mediation proportion (MP) via Mendelian randomization (MR), where the total causal effect θ is estimated via univariable MR and the direct effect α via multivariable MR (MVMR). Note how MR is based on solving a regression model, thus assuming that regressors are measured exactly, whereas this is not the case in practice. When the measurements are imprecise (i.e., the sample size underlying the summary estimates is small), the estimated causal effects suffer from regression dilution bias, also known as weak instrument bias. (C) Estimating mediation proportion ($\hat{\text{MP}}$) of the total causal effect between a single exposure-outcome pair and multiple related exposure-outcome pairs. (D) Our proposed maximum-likelihood-based approaches, LiMA and I-LiMA, for reducing the regression dilution bias.

However, conventional univariable MR analyses are confined to estimating the total causal effect between single exposure-outcome pairs and cannot capture more complex relationships. Mediation analyses extend this framework by decomposing total effects into their distinct direct and indirect counterparts^8^ (Figure 1A), allowing deeper insight into causal mechanisms and informing more nuanced intervention strategies. A particularly interesting application is to explore the complex mediatory role of molecular traits, such as metabolites and proteins. Metabolites—small molecules involved in metabolism—serve as biomarkers for disease processes and treatment response in clinical practice,^9^ while proteins—large molecules central to cellular functions—are implicated in various diseases and commonly used as drug targets.^10^ Identifying these mediators enhances biological understanding and guides biomarker discovery as well as drug development.

The most robust approaches for mediation analyses are also based on MR.^11^ In particular, multivariable MR (MVMR)^12^ retains all the desirable properties of conventional MR while simultaneously modeling the effect of many mediators, e.g., dozens of molecular traits in an omics layer.^13^ By incorporating mediators alongside the exposure in the MVMR model, the direct effect of the exposure on the outcome can be estimated, independent of the indirect effects, through any of the mediators. This encapsulates the two-step MR framework for mediation analyses: first, univariable MR is used to estimate the total causal effect of the exposure on the outcome; second, MVMR is employed to estimate the direct effect (Figure 1B). The mediation effect is derived by subtracting the direct effect from the total causal effect. If the direction of all these effects aligns (that is, the direct and mediation effects have the same sign), the mediation proportion (MP) of the total causal effect can be calculated^11^ (Figure 1C).

**Figure 2:**
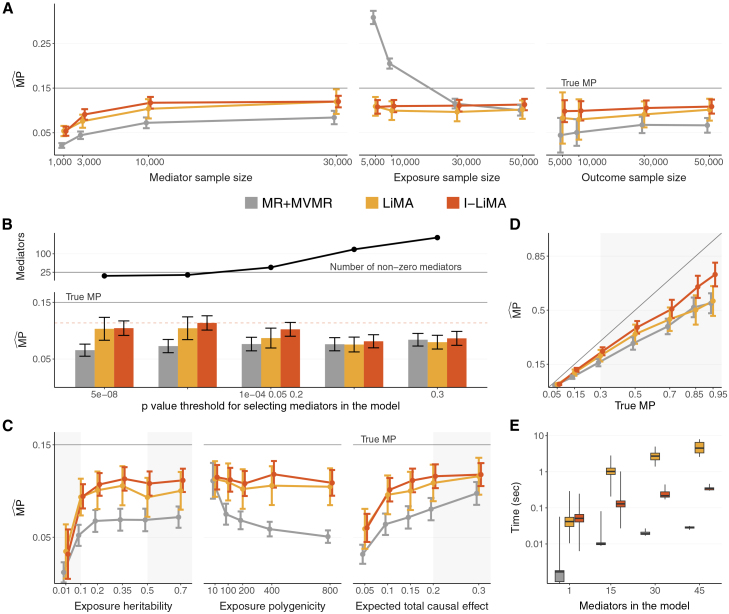
Results of the simulation study over the domains of relevant data, model, and sample characteristics (A–D) The parameter depicted on the *x* axis varies over its domain, while all other parameters used in the simulation have been fixed (Table S1). Error bars represent 95% confidence intervals of the estimates obtained over the simulations. (A) The influence of sample size of the mediators, exposure, and outcome on the $\hat{\text{MP}}$ bias. (B) The bias in $\hat{\text{MP}}$ influenced by null mediators in the model. A mediator is selected in the model if the *p* value of the test statistic measuring its significance passes a pre-specified threshold. (C) The influence of exposure heritability, exposure polygenicity (measured as the number of exposure instruments), and total causal effect on the $\hat{\text{MP}}$ bias. Shaded areas correspond to unrealistic values of these parameters. (D) Comparison of the mediation methods in terms of the bias in $\hat{\text{MP}}$ for different values of true MP. The shaded area corresponds to MP values that are unlikely to be observed in reality. (E) Comparison of the running time of the mediation methods (in logarithmic scale) in case of varying number of mediators in the model.

The two-step MR framework is conceptually straightforward, making it an attractive choice for mediation analyses across a wide range of settings.^11^^,^^12^^,^^13^^,^^14^^,^^15^ However, it does not directly solve the underlying mediation model. As MR, and especially MVMR, is prone to weak instrument bias, the two-step *ad hoc* nature of the MR framework means these biases are propagated and amplified in the estimation of MP. Direct and total effects are also commonly estimated from different data and instrument sets, introducing additional bias. The resulting bias in $\hat{\text{MP}}$ can be substantial enough to render the MR framework completely ineffective for performing mediation analyses when the sample sizes for the mediator and exposure are considerably different^13^^,^^16^ (Figure 2A) or when mediators are numerous. This vulnerability is particularly pronounced when investigating how molecular traits mediate causal effects between complex traits, as the summary data available from the largest quantitative trait locus (QTL) studies are based on sample sizes an order of magnitude smaller than those of complex trait GWASs.

In biomedical contexts, $\hat{\text{MP}}$ bias can misrepresent the perceived importance of biological pathways, potentially diverting resources toward less relevant targets or overlooking more impactful mediators in biomarker discovery and drug development. For instance, mediation analyses have been used to quantify the extent to which classical cardiovascular risk factors account for the elevated risk of cardiovascular events and all-cause mortality in patients with type 2 diabetes (T2D), suggesting that a substantial proportion of the risk remains unexplained by traditional factors, such as insulin resistance or elevated triglycerides.^17^ Accurate MP estimation is therefore critical for correctly identifying and prioritizing the most influential causal pathways and for guiding future research and intervention strategies.

To address this issue, we propose a maximum likelihood (ML) framework for mediation analysis that directly solves the mediation model (Figure 1D) using only association summary statistics. Our method, coined Likelihood-based Mediation Analysis (LiMA), allows for a more reliable estimation of MP compared to the conventional MR-based approach. We extend LiMA to handle hundreds of mediators by integrating out individual mediator effects and focusing on their combined mediatory effect of interest (methods). The resulting Integrated Likelihood-based Mediation Analysis (I-LiMA) method enables accurate estimation of the aggregated role of entire omics layers in the causal chain between two traits of interest. In extensive simulation analyses with realistic settings (Table S1), we demonstrate that I-LiMA is virtually immune to discrepancies in the sample sizes of the exposure and mediator studies and show how it resolves the biases in the estimation of MP (Figure 2).

We then apply the different mediation analysis methods to quantify the role of assayed proteome and metabolome in the mediation of causal relationships between classical epidemiological risk factors (such as obesity, high cholesterol, etc.) and cardiovascular outcomes. Next, we reveal the extent to which proteins and metabolites mediate the causal effects from risk factors to disease, offer a theoretical and practical guide to mediation analyses, demonstrate when and why the classical MR framework fails, and discuss how our ML approach mitigates the biases. We also provide R code for conducting mediation analyses using MR+MVMR as well as LiMA and I-LiMA.

## Methods

### Overview of the mediation framework

Let *X* denote an exposure and *Y* an outcome such that *X* has a non-zero causal effect *θ* on *Y*. Let ***M***: = (*M*_1_,*M*_2_, …,*M*_*k*_) represent *k* potential mediators of the *X*→*Y* relationship. Our aim is to dissect the total causal effect into a direct component *α* and a mediated component *ω*, such that *θ* = *α* + *ω*. In a directed acyclic graph (DAG), *α* represents the direct arrow from *X* to *Y*, while *ω* represents all directed paths from *X* to *Y* that go through ***M*** (Figure 1A). Thus, *ω* = ***γ***′***δ***, where ***γ***: = (*γ*_1_, …,*γ*_*k*_) denotes the causal effects of the exposure *X* on the mediators ***M*** and ***δ***: = (*δ*_1_, …,*δ*_*k*_) denotes the causal effects of the mediators ***M*** on the outcome *Y*.

The causal model we chose (Figure 1A) is a generalization of the MR model and requires three types of GWAS or QTL summary statistics to estimate key parameters—those of the exposure, the outcome, and all of the mediators. We used genetic instruments as anchors to estimate each of the direct and indirect causal effects (Figure 1A). Let ***G***: = (*G*_1_, …,*G*_*m*_) denote *m* instruments for the exposure *X* with effect sizes ***β***: = (*β*_1_, …,*β*_*m*_). Similarly, let ***F***: = (*F*_1_, …,*F*_*l*_) denote *l* instruments for the *k* mediators ***M***, where the effect of *F*_*i*_ on *M*_*j*_ is **B**_*i*,*j*_. Note that a genetic variant *F*_*i*_ is included only if it has a significant effect on at least one of the mediators. As a consequence, the *l* × *k* effect matrix **B** can be very sparse, but the same instrument may have a direct effect on multiple mediators. Let **C**: *m* × *k* and ***c***: = (*c*_*i*_, …,*c*_*m*_) denote the downstream effects of the exposure instruments ***G*** on the mediators ***M***—such that the effect of *G*_*i*_ on *M*_*j*_ is **C**_*i*,*j*_—and on the outcome *Y*, respectively. Similarly, let ***b***: = (*b*_*i*_, …,*b*_*l*_) denote the downstream effects of mediator instruments ***F*** on the outcome *Y*. We denote the effects of ***F*** on the exposure *X* as ***β***_***F***_.

We assume that the effects of ***F*** on *X* are all zero, ***β***_***F***_ = 0_*L*,1_. This assumption can be ensured by only using mediator instruments that have no detectable effects on the exposure. It also implies that ***F*** and exposure instruments ***G*** are independent. We also make use of the InSIDE (INstrument Strength Independent of Direct Effect) assumption, which is commonly made in causal inference studies, expecting any potential pleiotropic effects in the instruments to be uncorrelated with the strength of these instruments, with mean zero. To simplify the math in the derivations of the mediation models and to better interpret the effect estimates in our mediation framework, we further assume—without loss of generality—that *X*, *Y*, ***M***, ***G***, and ***F*** are all standardized to zero mean and unit variance. Finally, we assume the availability of association summary statistics $\hat{\beta }$, $\hat{C}$, $\hat{B}$, $\hat{c}$, and $\hat{b}$.

### Mediation via MR

The MP is commonly determined in a two-step MR approach^13^ (Figure 1B). First, the total causal effect *θ* is estimated by any univariable MR method. We assumed the independence of the instruments and applied the inverse-variance weighted (IVW) approach where the variances of individual Wald ratio estimates were approximated by the first-order Taylor expansion, $\text{Var}\left(\frac{{\hat{c}}_{i}}{{\hat{\beta }}_{i}}\right)\approx \frac{\text{Var}\left({\hat{c}}_{i}\right)}{{\hat{\beta }}_{i}}$,^18^^,^^19^ yielding the weighted least squares solution

$$\hat{\theta }={\left({\hat{\beta }}^{\prime }\Lambda_{\hat{c}}^{-1}\hat{\beta }\right)}^{-1}\cdot {\hat{\beta }}^{\prime }\Lambda_{\hat{c}}^{-1}\hat{c}\text{,}$$

where $\Lambda_{\hat{c}}:m\times m$ is a diagonal matrix with ${\left(\Lambda_{\hat{c}}\right)}_{i,i}=\text{Var}\left({\hat{c}}_{i}\right)$ on the diagonal. If these diagonal variances were not available and the instruments were allowed to be correlated, then $\Lambda_{\hat{c}}$ could be replaced by the linkage disequilibrium (LD) correlation matrix, estimable from reference samples.

Second, the direct causal effect *α* is estimated by MVMR. For this, we use the instruments described above (***G*** and ***F***), i.e., variants that are associated with either the exposure or with any of the mediators. We relied on the IVW approach once again to yield

$$\left(\begin{array}{l} \hat{\alpha }\\ \hat{\delta } \end{array}\right)=\left({\left({\hat{\Pi }}^{\prime }\Lambda_{\hat{\pi }}^{-1}\hat{\Pi }\right)}^{-1}{\hat{\Pi }}^{\prime }\Lambda_{\hat{\pi }}^{-1}\hat{\pi }\right)\text{,}$$

where $\hat{\Pi }:=\left[\begin{array}{cc} \hat{\beta } & \hat{C}\\ {\hat{\beta }}_{F} & \hat{B} \end{array}\right]:\left(m+l\right)\times \left(k+1\right)$ is a block matrix of instrument effects on the exposure and mediators, $\hat{\pi }:={\left[\hat{c}\;\hat{b}\right]}^{\prime }$ is an (*m* + *l*) vector of instrument effects on the outcome, and $\Lambda_{\hat{\pi }}:=\text{diag}\left(\Lambda_{\hat{c}},\Lambda_{\hat{b}}\right)$ is a block diagonal matrix of the variances of these effects, i.e., $\Lambda_{\hat{c}}$ is as previously defined and $\Lambda_{\hat{b}}:l\times l$ is a diagonal matrix with ${\left(\Lambda_{\hat{b}}\right)}_{i,i}=\text{Var}\left({\hat{b}}_{i}\right)$ on the diagonal. Similarly to univariable MR, related instruments could be facilitated by replacing $\Lambda_{\hat{\pi }}$ with the LD matrix. Note that both IVW estimators (for $\hat{\theta }$ and $\hat{\alpha }$) are consistent under the InSIDE assumption made in our mediation framework.

The MR framework for mediation analyses is called three-sample MVMR (3S-MVMR) when independent data sources are used for the summary statistics of *X*, ***M***, and *Y*.^13^ The sample sizes underlying these summary statistics can differ considerably. For example, a complex exposure *X* is often based on a sample size at least an order of magnitude larger than that of molecular mediators ***M***. In this case, MVMR tends to overestimate the direct effect *α* from the exposure to the outcome,^13^ leading to an underestimation of the indirect (mediated) effect due to regression dilution bias, which becomes more pronounced as the mediator sample size decreases.^16^

### LiMA

To overcome the biases in mediation analyses induced by the conventional MR-based approach, we propose LiMA:

$$\left(\begin{array}{l} \hat{\gamma }\\ \begin{array}{c} \hat{\delta }\\ \hat{\alpha } \end{array} \end{array}\right)=\begin{array}{c} \arg\;\;\max\;\;\mathrm{Pr}\\ \gamma ,\delta ,\alpha \end{array}\left(\hat{c},\hat{b},\hat{C},\hat{B},\hat{\beta }\;|\;\gamma ,\delta ,\alpha \right),\hat{\theta }=\hat{\alpha }+\hat{\gamma }\hat{\delta }\text{.}$$

Similarly to the MR+MVMR approach, we assume once again the independence of all instruments. We further assume that mediator and outcome studies are independent with no sample overlap. This is often the case for omics mediators and complex outcome traits and is implicitly assumed in causal analyses based on two-sample MR. Then, the joint likelihood is

(Equation 1) $$\mathrm{Pr}\left(\hat{c},\hat{b},\hat{C},\hat{B},\hat{\beta }\;|\;\alpha ,\gamma ,\delta \right)=\mathrm{Pr}\left(\hat{c},\hat{b},\hat{C}\;|\;\hat{B},\hat{\beta },\alpha ,\gamma ,\delta \right)\cdot \mathrm{Pr}\left(\hat{B},\hat{\beta }\;|\;\alpha ,\gamma ,\delta \right)\propto \mathrm{Pr}\left(\hat{c},\hat{b},\hat{C}\;|\;\hat{B},\hat{\beta },\alpha ,\gamma ,\delta \right)=\mathrm{Pr}\left(\hat{b}\;|\;\hat{B},\delta \right)\cdot \mathrm{Pr}\left(\hat{c}\;|\;\hat{\beta },\alpha ,\gamma ,\delta \right)\cdot \mathrm{Pr}\left(\hat{C}\;|\;\hat{\beta },\gamma \right)\text{,}$$

where we made use of the fact that instrument effects on the exposure and mediators do not depend on the causal effects, reducing the conditional likelihood for $\hat{\beta }$ and $\hat{B}$ to a constant.

The likelihood function is constructed to directly mirror the mediation model (represented by the DAG in Figure 1A), and the causal effects *α*, ***γ***, and ***δ*** can be estimated by maximizing the joint likelihood function for the observed summary statistics data (Figure 1D). As a consequence, all measurement errors in the genetic effects of *X* and ***M*** are accounted for, resulting in less bias in MP estimation (Figure 2).

To be able to optimize the likelihood function (Equation 1) based on our mediation framework, we make a few additional assumptions. First, we assume (due to asymptotic properties) that estimates $\hat{\beta }$, $\hat{C}$, $\hat{B}$, $\hat{c}$, and $\hat{b}$ all follow Gaussian distributions. Second, we assume that the effect sizes for all mediators ***M*** have been estimated from the same sample. This is a slightly stronger assumption, but it is reasonable when mediators come from the same dataset, such as gene expression QTLs (eQTLs). Under these conditions, our mediation model can then be written as follows (see supplemental methods for more details):

(Equation 2) $$\hat{\beta }∼N_{m}\left(\beta ,\frac{1}{n_{X}}\cdot I_{m}\right)\text{,}$$

(Equation 3) $$\text{vec}\left(\hat{B}\right)∼N_{lk}\left(\text{vec}\left(B\right),\frac{1}{n_{M}}\cdot \Sigma ⊗I_{l}\right)\text{,}$$

(Equation 4) $$\text{vec}\left(\hat{C}\right)∼N_{mk}\left(\text{vec}\left(\beta \gamma^{\prime }\right),\left(\frac{1}{n_{M}}+\sigma_{C}^{2}\right)\cdot \Sigma ⊗I_{m}\right)\text{,}$$

(Equation 5) $$\hat{c}∼N_{m}\left(\beta \left(\alpha +\gamma^{\prime }\delta \right),\left(\frac{1}{n_{Y}}+\sigma_{c}^{2}\right)\cdot I_{m}\right),\text{and}$$

(Equation 6) $$\hat{b}∼N_{l}\left(B\delta ,\left(\frac{1}{n_{Y}}+\sigma_{b}^{2}\right)\cdot I_{l}\right)\text{,}$$

where $\sigma_{C}^{2}$, $\sigma_{c}^{2}$, and $\sigma_{b}^{2}$ denote additional variance components due to potential pleiotropy; Σ: *k* × *k* is the phenotypic correlation matrix of the mediators (or in case the mediator effects are estimated in partially overlapping samples, Σ_*i*,*j*_ is the cross-trait LD score regression intercept for mediators *M*_*i*_ and *M*_*j*_); and *n*_*X*_, *n*_*Y*_, and *n*_*M*_ are the sample sizes of the exposure, outcome, and mediator studies, respectively. Variances of the effect size estimates reduce—approximately—to the inverse of the studies’ sample sizes because, as per our mediation framework, all the variables were standardized to unit variance.

To reduce numeric overflow in likelihood calculations, we optimized the logarithm of the likelihood function:

(Equation 7) $$\log\left(\mathrm{Pr}\left(\hat{c},\hat{b},\hat{C},\hat{B},\hat{\beta }\;|\;\alpha ,\gamma ,\delta \right)\right)\propto \sum_{i=1}^{l}\log\left(\mathrm{Pr}\left({\hat{b}}_{i}\;|\;{\hat{B}}_{i,\cdot },\delta \right)\right)+\sum_{i=1}^{m}\log\left(\mathrm{Pr}\left({\hat{c}}_{i}\;|\;{\hat{\beta }}_{i},\alpha ,\gamma ,\delta \right)\right)+\sum_{i=1}^{m}\log\left(\mathrm{Pr}\left({\hat{C}}_{i,\cdot }\;|\;\hat{\beta },\gamma \right)\right)\text{,}$$

where we made use of the assumption of independent instruments to yield a more manageable sum of independent log likelihoods. LiMA estimates the direct effect *α* together with indirect effects ***γ*** and ***δ*** by maximizing the joint log likelihood function (Equation 7). The total indirect effect is estimated as $\hat{\omega }={\hat{\gamma }}^{\prime }\hat{\delta }$, and the total causal effect is estimated as $\hat{\theta }=\hat{\alpha }+\hat{\omega }$.

To speed up LiMA—via more efficient matrix inversions and determinant calculations—we further simplified the individual log-likelihood components in Equation 7. Interested readers can find these derivations in the supplemental methods.

### I-LiMA

Increasing the number of mediators requires the estimation of more parameters (***γ*** and ***δ***) by LiMA, leading to increased algorithmic complexity and runtime (Table 1). Thus, optimizing the likelihood quickly becomes impractical and time-consuming, even when using our above-mentioned simplifications (Figure 2E). However, note that it is not necessary to know the causal effect through each individual mediator, as estimating MP only requires the total mediation effect (***γ***′ · ***δ***). Hence, we extended LiMA by integrating out ***γ*** and ***δ***:

(Equation 8) $$\begin{array}{c} \left(\begin{array}{l} \begin{array}{c} \hat{\alpha }\\ {\hat{\sigma }}_{\gamma }^{2} \end{array}\\ \begin{array}{c} {\hat{\sigma }}_{\delta }^{2}\\ {\hat{\sigma }}_{\gamma ,\delta } \end{array} \end{array}\right)\\ \hat{\theta }=\hat{\alpha }+k.{\hat{\sigma }}_{\gamma ,\delta } \end{array}=\begin{array}{c} \arg\;\;\max\\ \alpha ,\sigma_{\gamma }^{2},\sigma_{\delta }^{2},\sigma_{\gamma ,\delta } \end{array}\int \mathrm{Pr}\left(\hat{c},\hat{b},\hat{C},\hat{B},\hat{\beta }\;|\;\gamma ,\delta ,\alpha \right)\mathrm{Pr}\left(\gamma ,\delta \right)d\left(\gamma ,\delta \right)\text{,}$$

where we assumed a Gaussian prior for individual mediation effects:

(Equation 9) $$\left(\begin{array}{l} \gamma \\ \delta \end{array}\right)∼N\left(\left(\begin{array}{l} 0\\ 0 \end{array}\right),\left(\begin{array}{cc} \sigma_{\gamma }^{2}\cdot I_{k} & \sigma_{\gamma ,\delta }\cdot I_{k}\\ \sigma_{\gamma ,\delta }\cdot I_{k} & \sigma_{\delta }^{2}\cdot I_{k} \end{array}\right)\right)\text{.}$$

**Table 1:** Computation characteristics and performance metrics of mediation methods

		**Computation characteristics**			**Performance metrics**^a^			
**Methods**		**Algorithmic complexity**^b^	**Params**^c^	**Runtime**^d^	**|Bias|**	**Coverage**	**Power**^e^	**T1E**
Exact	MR+MVMR	*O*(*k*^3^ + *k*^2^*m* + *km*)	*k* + 2	0.01	51.6%	38.2%	63.1%	24.7%
Approx.	LiMA	*O*(*t*_1_(*k*^3^ + *k*^2^*m* + *k* + *m*))	2*k*	1.02	31.3%	17.8%	81.4%	30.1%
	I-LiMA	*O*(*t*_2_(*k*^3^ + *k*^2^*m* + *km*^2^))	4	0.13	24.5%	63.2%	53.1%	11.5%

a Reported metrics are based on the default settings used in the simulation study (Tables S1 and S2). Type I error (T1E) is based on *k* = 1 in Figure 3E.

b The algorithmic complexity of a single iteration over a method’s objective function depends on the number of mediators (*k*), mediator instruments (*l*), and exposure instruments (*m*). Since E(*l*) = const·*k*, we have grouped the *l* terms under *k* terms for simplicity. Approximate methods need additional iterations to converge to a solution—*t*_1_ or *t*_2_ in the table—which depends on the number of parameters, shape of the objective function, and other criteria.

c The number of parameters directly estimated by the methods. In the MR framework, this is done in two steps, as *θ* is estimated with univariable MR and the other *k* + 1 parameters with MVMR. LiMA estimates all the parameters at the same time, while I-LiMA takes three steps, estimating $\sigma_{\gamma }^{2}$ and $\sigma_{\delta }^{2}$ separately from *α* and *σ*_*γ*,*δ*_ (methods).

d Median running time (in seconds) of the methods (implemented in R) based on the simulation study with 15 mediators in the model (Figure 2E). Computing was performed on an x86_64 Linux system using a single core of an AMD EPYC 7443 2.85 GHz CPU.

e Power values should be interpreted with caution when T1E is not adequately controlled, as inflated error rates can lead to overestimated power.

We refer to this approach as I-LiMA. Mediator independence is assumed to simplify the marginal likelihood for computational purposes. Our simulations show this to be an acceptable trade-off, even when the mediators are strongly correlated (Figures S1 and S2).

The prior parameters $\sigma_{\gamma }^{2}$, $\sigma_{\delta }^{2}$, and *σ*_*γ*,*δ*_ determine the joint variability of the indirect effects but also have other functional importance. Most crucially, the covariance of the indirect effects *σ*_*γ*,*δ*_, along with the number of mediators *k*, controls the expected value of the total indirect effect *ω*:

(Equation 10) $$E\left(\omega \right)=E\left(\gamma^{\prime }\delta \right)=k\cdot E\left(\gamma_{i}\delta_{i}\right)=k\cdot \sigma_{\gamma ,\delta }\text{.}$$

The variance of *ω* further depends on the prior variances $\sigma_{\gamma }^{2}$ and $\sigma_{\delta }^{2}$:

(Equation 11) $$\sigma_{\omega }^{2}:=\text{Var}\left(\omega \right)=\text{Var}\left(\gamma^{\prime }\delta \right)=k\cdot \text{Var}\left(\gamma_{i}\delta_{i}\right)=k\cdot \left(\sigma_{\gamma }^{2}\sigma_{\delta }^{2}+\sigma_{\gamma ,\delta }^{2}\right)\text{.}$$

Obtaining reliable estimates of MP strongly relies on accurately estimating these prior parameters (Figure S10; supplemental methods).

#### Deriving the marginal distribution

Since each component of the joint likelihood function (Equation 1) follows a Gaussian distribution, the joint likelihood itself is a multivariate Gaussian:

$$\mathrm{Pr}\left(\hat{c},\hat{b},\hat{C},\hat{B},\hat{\beta }\;|\;\alpha ,\gamma ,\delta \right)=\mathrm{Pr}\left(\hat{b}\;|\;\hat{B},\delta \right)\cdot \mathrm{Pr}\left(\hat{c}\;|\;\hat{\beta },\alpha ,\gamma ,\delta \right)\cdot \mathrm{Pr}\left(\hat{C}\;|\;\hat{\beta },\gamma \right)∼N\left(\left(\begin{array}{l} \hat{B}\delta \\ \hat{\beta }\cdot \left(\alpha +\gamma^{\prime }\delta \right)\\ \text{vec}\left(\hat{\beta }\gamma^{\prime }\right) \end{array}\right),\left(\begin{array}{ccc} \Lambda_{s_{b}^{2}} & 0 & 0\\ 0 & \Lambda_{s_{c}^{2}} & 0\\ 0 & 0 & S_{C}⊗I_{m} \end{array}\right)\right)\text{.}$$

Hence, the marginal distribution in Equation 8 is also a Gaussian. Furthermore, as any other Gaussian, it is defined by its mean and variance. Deriving these is a lengthy affair, which we have left for the supplemental methods. Here, we simply express the final form of the marginal distribution, which depends on the direct effect *α* and prior parameters $\sigma_{\gamma }^{2}$, $\sigma_{\delta }^{2}$, and *σ*_*γ*,*δ*_:

(Equation 12) $$N\left(\left(\begin{array}{l} 0\\ \hat{\beta }\left(\alpha +k\sigma_{\gamma ,\delta }\right)\\ 0 \end{array}\right),\left(\begin{array}{ccc} \Lambda_{\mu_{s_{b}^{2}}}+\sigma_{\delta }^{2}\hat{B}{\hat{B}}^{\prime } & 0 & \sigma_{\gamma ,\delta }\cdot \left(\hat{B}⊗{\hat{\beta }}^{\prime }\right)\\ 0 & \Lambda_{\mu_{s_{c}^{2}}}+\sigma_{\omega }^{2}\hat{\beta }{\hat{\beta }}^{\prime } & 0\\ \sigma_{\gamma ,\delta }\cdot \left({\hat{B}}^{\prime }⊗\hat{\beta }\right) & 0 & \Sigma ⊗\Lambda_{s_{M}^{2}}+I_{k}⊗\sigma_{\gamma }^{2}\left(\Lambda_{n_{X}^{-1}}+\hat{\beta }{\hat{\beta }}^{\prime }\right) \end{array}\right)\right)\text{,}$$

where Σ still refers to the phenotypic correlation matrix of the mediators, and

$$\begin{array}{l} \mu_{s_{b}^{2}}:=E\left(s_{b}^{2}\right)=\frac{1}{n_{Y}}+\sigma_{b}^{2}+\frac{1}{n_{M}}\cdot k\sigma_{\delta }^{2}\text{,}\\ \mu_{s_{c}^{2}}:=E\left(s_{c}^{2}\right)=\frac{1}{n_{Y}}+\sigma_{c}^{2}+\frac{1}{n_{X}}\cdot \left(\sigma_{\omega }^{2}+{\left(\alpha +k\sigma_{\gamma ,\delta }\right)}^{2}\right)\text{,}\\ s_{M}^{2}:=\frac{1}{n_{M}}+\sigma_{C}^{2}\text{,} \end{array}$$

where the parameters $\sigma_{c}^{2}$, $\sigma_{b}^{2}$, and $\sigma_{C}^{2}$ control the level of pleiotropy in the summary statistics data and can also be optimized for.

I-LiMA estimates the direct effect *α* together with the total indirect-effect-controlling variance components $\sigma_{\gamma }^{2}$, $\sigma_{\delta }^{2}$, and *σ*_*γ*,*δ*_ by maximizing the logarithm of the marginal likelihood represented by Equation 12. The total indirect effect is estimated as $\hat{\omega }=k{\hat{\sigma }}_{\gamma ,\delta }$, and the total causal effect is estimated as $\hat{\theta }=\hat{\alpha }+\hat{\omega }$.

The covariance matrix in the marginal likelihood (Equation 12) has dimensions (*mk* + *m* + *l*) × (*mk* + *m* + *l*), where the number of instruments of a complex polygenic trait can easily be *m* > 100, the number of potential mediators to consider in omics layers can be *k* > 100, and the total number of mediator instruments *l* can be even bigger. Large matrix operations can render the optimization infeasible due to high computation costs. To mitigate this complexity, we actually further simplified the marginal likelihood, relying on the assumption Σ = I_*k*_. Interested readers can find these derivations in the supplemental methods.

### Variances of the direct and total causal effect estimates

In the MR+MVMR approach, $\hat{\alpha }$ and $\hat{\theta }$ are OLS estimators; thus, their variances $\text{Var}\left(\hat{\alpha }\right)$ and $\text{Var}\left(\hat{\theta }\right)$ simply follow from the standard variance formulae of the ordinary least squares (OLS) regression. To estimate these variances in LiMA and I-LiMA, we relied on the likelihood-ratio test (LRT):

$$\text{Var}\left(\hat{\alpha }\right)=\frac{\left|\hat{\alpha }\right|}{\sqrt{{\hat{\chi }}_{\alpha }^{2}}},\text{Var}\left(\hat{\omega }\right)=\frac{\left|\hat{\omega }\right|}{\sqrt{{\hat{\chi }}_{\omega }^{2}}},\text{Var}\left(\hat{\theta }\right)=\text{Var}\left(\hat{\alpha }\right)+\text{Var}\left(\hat{\omega }\right)\text{,}$$

where $\hat{\alpha }$ and $\hat{\omega }$ are now ML estimates of either method, while ${\hat{\chi }}_{\alpha }^{2}$ and ${\hat{\chi }}_{\omega }^{2}$ are LRT statistics of appropriate likelihood function L(α,ω):

$${\hat{\chi }}_{\alpha }^{2}=-2\cdot \log\frac{L\left(\alpha =0,\omega =\hat{\omega }\right)}{L\left(\alpha =\hat{\alpha },\omega =\hat{\omega }\right)},{\hat{\chi }}_{\omega }^{2}=-2\cdot \log\frac{L\left(\alpha =\hat{\alpha },\omega =0\right)}{L\left(\alpha =\hat{\alpha },\omega =\hat{\omega }\right)}\text{.}$$

For LiMA, we used Equation 5 for the likelihood L(α,ω) as the only component that depends on *α* and *ω* = ***γ***′***δ***. For I-LiMA, we used the full marginal likelihood (Equation 12).

### Estimating the MP

For a single exposure and outcome pair, we estimated the MP by the simple ratio of indirect effect on the total causal effect and approximated its variance by the Delta method:

$$\hat{\text{MP}}=1-\frac{\hat{\alpha }}{\hat{\theta }},\text{Var}\left(\hat{\text{MP}}\right)=\frac{{\hat{\alpha }}^{2}}{{\hat{\theta }}^{2}}\cdot \left(\frac{\text{Var}\left(\hat{\alpha }\right)}{{\hat{\alpha }}^{2}}+\frac{\text{Var}\left(\hat{\theta }\right)}{{\hat{\theta }}^{2}}\right)\text{.}$$

The estimator $\hat{\text{MP}}$ is valid under the assumptions of our mediation framework (Figure 1), including when the mediator also influences the exposure (reverse causation).^20^^,^^21^

For groups of exposure-outcome pairs, we estimated the average MP by a simple linear regression of the indirect effects on the total causal effects, without the intercept^13^:

(Equation 13) $$\hat{\text{MP}}=1-\frac{\sum_{i=1}^{n}{\hat{\alpha }}_{i}\cdot {\hat{\theta }}_{i}}{\sum_{i=1}^{n}{\hat{\theta }}_{i}^{2}},\text{Var}\left(\hat{\text{MP}}\right)=\frac{\sigma^{2}}{\sum_{i=1}^{n}{\hat{\theta }}_{i}^{2}}\text{,}$$

where *σ*^2^ denotes the error variance in the regression model.

We did not consider MP estimates and excluded from the analysis those rare cases in which the likelihood-based optimization failed to converge (Table S7).

### Simulation study

In order to test the performance of the mediation methods, we performed simulations under various realistic scenarios. For each scenario, we set up 300 different mediation analyses by generating artificial causal effects *α*, ***γ***, and ***δ*** and simulating the summary statistics $\hat{\beta }$, $\hat{B}$, $\hat{C}$, $\hat{c}$, and $\hat{b}$ with respective variances. Each time, we estimated the direct and total causal effects under both the MR framework and our proposed ML approaches. The estimates $\left({\hat{\alpha }}_{1},{\hat{\theta }}_{1}\right),\ldots ,\left({\hat{\alpha }}_{n},{\hat{\theta }}_{n}\right)$ for each mediation method in each scenario were then plugged into Equation 13 to calculate $\hat{\text{MP}}$ and its variance. These results were used to compare the methods in terms of bias and variability.

#### Generating mediation effects

We assumed the mediation effects (*γ*_*i*_,*δ*_*i*_) are independent identically distributed random variables, following a Gaussian spike-and-slab distribution

(Equation 14) $$\left(\begin{array}{l} \gamma_{i}\\ \delta_{i} \end{array}\right)∼\left\{ \begin{array}{cc} N\left(0,\left(\begin{array}{cc} \sigma_{\gamma }^{2} & \sigma_{\gamma ,\delta }\\ \sigma_{\gamma ,\delta } & \sigma_{\delta }^{2} \end{array}\right)\right), & i=1,\ldots ,k_{s}\\ 0, & i=k_{s}+1,\ldots ,k \end{array}\right.\text{,}$$

where *k* is the total number of mediators, *k*_*s*_ = *p*_*k*_·*k* denotes the proportion *p*_*k*_ of non-zero mediators, and the Gaussian slab is the same as in Equation 9. Running any simulation requires plugging in for the variances $\sigma_{\gamma }^{2}$ and $\sigma_{\delta }^{2}$, together with the covariance *σ*_*γ*,*δ*_.

Let $\sigma_{Y,M}^{2}$ denote the outcome variance explained by the mediators. Since $Y=X\alpha +\sum_{i=1}^{k_{s}}M_{i}\delta_{i}+\epsilon$ and we assumed *X*, *Y*, and ***M*** to all be standardized to zero mean and unit variance, we can approximate it as $\sigma_{Y,M}^{2}\approx \sum_{i=1}^{k_{s}}\delta_{i}^{2}$. We make use of Equations 10 and 11 to approximate the unknown variance components of Equation 14 in terms of the squared indirect effect:

$${\left(\text{MP}\cdot E\left(\theta \right)\right)}^{2}=E\left(\omega^{2}\right)=\text{Var}\left(\omega \right)+{\left(E\left(\omega \right)\right)}^{2}=k_{s}\sigma_{\gamma }^{2}\sigma_{\delta }^{2}+\left(k_{s}^{2}+k_{s}\right)\rho_{\gamma ,\delta }^{2}\sigma_{\gamma }^{2}\sigma_{\delta }^{2}\text{,}$$

where *ρ*_*γ*,*δ*_ denotes the correlation between the mediation effects. All the unknown components of the (*γ*_*i*_,*δ*_*i*_) covariance matrix can thus be calculated as follows:

$$\begin{array}{l} \sigma_{\delta }^{2}=\frac{1}{k_{s}}\cdot \sum_{i=1}^{k_{s}}\delta_{i}^{2}=\frac{1}{k_{s}}\cdot \sigma_{Y,M}^{2}\text{,}\\ \sigma_{\gamma }^{2}=\frac{{\left(\text{MP}\cdot E\left(\theta \right)\right)}^{2}}{k_{s}\sigma_{\delta }^{2}+\left(k_{s}^{2}+k_{s}\right)\rho_{\gamma ,\delta }^{2}\sigma_{\delta }^{2}}\text{,}\\ \sigma_{\gamma ,\delta }=\rho_{\gamma ,\delta }\cdot \sigma_{\gamma }\sigma_{\delta }\text{,} \end{array}$$

where realistic values for $\sigma_{Y,M}^{2}$, *MP*, E(*θ*), and *ρ*_*γ*,*δ*_ are provided over their domains of function, leveraging real data applications.

Once the indirect effects have been generated, the direct effect easily follows:

$$\alpha =\frac{1-\text{MP}}{\text{MP}}\cdot \sum_{i=1}^{k_{s}}\gamma_{i}\delta_{i}\text{.}$$

The only exception to this is when MP = 0. In order to investigate the type I error (T1E) rate of the mediation analysis methods, we simply generated *α* values uniformly around E(*θ*).

#### Generating effect vectors and matrices

Let mediators *M*_*j*_ have heritabilities $h_{M_{j}}^{2}$ with an effective number of instruments *l*_*j*_. We generated realistic values for these quantities by using the gene expression summary statistics of the eQTLGen Consortium.^5^ As such, we approximated the distribution for the number of mediator instruments, *l*_*j*_, by the number of pruned (in a 500 kb window) significant *cis*-eQTLs of genes in the eQTLGen data (Table S9). For each gene, we considered the variance explained as measured by *R*^2^ of the gene’s top eQTLs as a sufficient proxy to its narrow-sense heritability. We approximated the distribution of these *R*^2^—and thus mediator $h_{M_{j}}^{2}$ values in our simulation study—by a Weibull distribution with shape 0.5 and scale 0.05, truncated to [0,1] (Figure S11). This approximation generalizes well to protein data.^22^

To run simulations, we also relied on the exposure heritability $h_{X}^{2}$ and polygenicity (proxied by the number of exposure instruments) *m*. These we controlled directly, allowing them to take different realistic values, depending on the simulation scenario. Thus, we could generate true values for instrument effects **B** and ***β***,

$$\begin{array}{l} B_{i,j}∼\left\{ \begin{array}{cc} N\left(0,\frac{h_{M_{j}}^{2}}{l_{j}}\right), & \text{if}\;\sum_{u=1}^{j-1}l_{u}+1\leq i\leq \sum_{u=1}^{j}l_{u}\\ 0, & \text{otherwise} \end{array}\right.\text{,}\\ \beta ∼N\left(0_{m},\frac{h_{X}^{2}}{m}\cdot I_{m}\right)\text{,} \end{array}$$

which we scaled post hoc to match the heritabilities. We then used Equations 2, 3, 4, 5, and 6—controlling the level of pleiotropy by fixing $\sigma_{C}^{2}$, $\sigma_{c}^{2}$, and $\sigma_{b}^{2}$ to different values (Figure S9)—to generate the estimates $\hat{\beta }$, $\hat{B}$, $\hat{C}$, $\hat{c}$, and $\hat{b}$, respectively.

#### Generating the mediator correlation matrix

Generating noise for $\hat{B}$ and $\hat{C}$ requires the mediator covariance matrix Σ. We used different strategies for this (Figure S1): (1) sampling from the covariance matrix calculated based on the gene expression data in the CoLaus cohort,^23^ (2) substituting with an identity matrix, and (3) generating random vector-based correlations Σ_*i*,*j*_ = Cor(*V*_*i*_,*V*_*j*_), where $V_{i},V_{j}∼N\left(0,I_{10}\right)$. Note that due to the low number of simulated variables (10), the empirical covariance matrix will be far from the identity. If necessary, to ensure full rank Σ in case of a large number of mediators *k*, we shrank the generated matrix slightly toward identity (shrinkage *λ* = 10^−6^). We used Cholesky decomposition of the final Σ to speed up the matrix computations in Equations 3 and 4.

#### Simulation procedure

Each scenario with its generated data represents a fictional study, albeit constructed to be as realistic as possible. To this end, we also incorporated the mediator selection into the models. We tested for a causal effect of the exposure on each of the mediators with IVW MR and included only those mediators in the analysis whose *p* value passed a fixed threshold *P*. We varied this threshold to assess the impact of omitting non-zero mediators or including null mediators on MP estimation bias (Figure 2B). If no mediators met the threshold, the simulation was repeated with newly generated data, which might have introduced some winner’s curse.

Altogether, we used many different parameters in our exhaustive simulation study: sample size of the exposure (*n*_*X*_), sample size of the mediators (*n*_*M*_), sample size of the outcome (*n*_*Y*_), number of exposure instruments (*m*), number of mediators (*k*), proportion of significant mediators (*p*_*k*_), *p* value threshold for selecting mediators in the analysis (*P*), expected total causal effect (E(*θ*)), mediation proportion (MP), exposure heritability ($h_{X}^{2}$), outcome variance explained by the mediators ($\sigma_{Y,M}^{2}$), correlation of mediation effects (*ρ*_*γ*,*δ*_), the type of mediator correlation matrix (Σ), and pleiotropy components ($\sigma_{C}^{2}$, $\sigma_{c}^{2}$, and $\sigma_{b}^{2}$). We investigated the individual influence of each parameter on mediation analysis by running simulations in which the parameter of interest varied across its respective domain, while the remaining parameters were held constant (Table S1).

### Mediation study

We studied mediation of pairwise causal effects between a set of 43 medically relevant traits from the UK Biobank (UKBB) (Table S8) that could inform on the molecular mechanisms through which the risk factors contribute to disease progression.^24^ We used summary statistics from Neale Lab UKBB v.3 (http://www.nealelab.is/uk-biobank). With the exception of binary traits, which were only considered as outcomes, each pair of traits was first tested for bidirectional causal effects with IVW MR. If the trait pair had a Bonferroni-corrected significant total causal effect in either direction, we studied how much of this total causal effect from the corresponding exposure to the outcome was mediated by proteins and metabolites. For protein QTLs, we used the INTERVAL study^22^; for metabolite QTLs, we used the study by Lotta et al.^9^ As such, we used only summary statistics from publicly available data sources.

#### Harmonizing the summary statistics

To match and combine summary statistics from different sources for the mediation analysis, we harmonized the data. Throughout, we used the human genome reference GRCh37.

All the effect sizes and standard errors were transformed to correspond to random variables with zero mean and unit variance. Further, we only worked with biallelic unambiguous (i.e., non-palindromic alleles) single-nucleotide polymorphisms (SNPs) that were available and had matching alleles in the relevant data, including the UK10K resource,^25^^,^^26^ which we used as the reference panel. All other genetic variants were filtered out. The effect alleles of the remaining variants were oriented to the minor alleles of the reference data. We preferred the UK10K panel over other public resources, such as the International HapMap Project^27^ or the 1000 Genomes Project,^28^ due to a larger sample size (*n* = 3,781) and similar ancestry with the UKBB participants.

#### MR between exposure-outcome pairs

For an MR analysis between an exposure-outcome pair, we used only strong instruments. Qualifying genetic variants were required to have a genome-wide significant effect (*p* < 5 × 10^−8^) on the exposure. Among those, through a procedure called Steiger filtering,^29^ we eliminated variants exhibiting an even stronger causal effect on the outcome (*p* < 0.05), which would indicate an indirect effect through reverse causation. Finally, to obtain a roughly independent set of instruments, we further removed genetic variants in LD by clumping with Plink-1.9,^30^ ensuring that no instruments closer than 10,000 kb to each other had *R*^2^ > 0.001.

If only a single instrument remained, we estimated the causal effect with an MR Wald ratio estimator; otherwise, we did so with an IVW MR as implemented in TwoSampleMR (https://mrcieu.github.io/TwoSampleMR/), an R package curated by MR-Base.^1^ If no strong instruments could be found for the exposure, the MR analysis was not performed.

#### Selecting mediators into the mediation model

Along with instruments for the exposure, the mediation framework also requires that all mediators be instrumented. Thus, each mediator selected into the analysis was required to have at least one strong instrument in our data (*p* < 5 × 10^−8^). Further, our simulation study showed no benefit of including mediators without a non-zero mediation effect (Figure 2B). Consequently, selected mediators were also required to have at least nominally significant (*p* < 0.05) causal effects from the exposure and to the outcome.

To determine the causal effect from the exposure to the mediator and from the mediator to the outcome, we used MR once again. With metabolites from the Lotta et al. study, for each trait pair, we performed MR following the exact procedure laid out in the section above. Since protein data were much more abundant, we made a few approximations to this procedure for computational reasons.

With the INTERVAL study, we first found the genetic variants overlapping between all the proteins in the data source, all the exposures and outcomes selected into the mediation analyses (i.e., with non-zero causal effect between them), and the reference panel. In the same way as before but considering only the overlapping variants, we clumped the genome-wide significant potential instruments in all of the exposures and all of the proteins. In case of each trait pair, we further applied Steiger filtering and only then performed MR. In ideal settings, Steiger filtering (like any other filtering procedure) would have been done before LD clumping. We did it retrospectively due to computational reasons—to avoid LD clumping each mediator’s data for every exposure and outcome. We expect the potential impact on the analysis from our chosen order of steps (should there be any) to be negligible.

#### Constructing the mediation model

Having identified promising mediators for an exposure-outcome pair, we made a few additional steps to accommodate the assumptions of our mediation framework. First, we specified a common set of instruments for all of the mediators. Chosen variants were required—along with genome-wide significance and the availability in all of the data sources—to pass Steiger filtering with both the exposure and outcome. This was to satisfy the exclusion restriction assumption of MR. If, based on the identified set of instruments, the selected mediators were correlated, we pruned them in a forward stepwise manner. We prioritized mediators with stronger mediation effects and ensured that no two mediators shared a correlation greater than 0.1. To ensure that instruments of all mediators taken together were roughly independent, we performed another round of LD clumping with the instruments of the remaining mediators. Here, the strength of association for each instrument was defined by the minimal *p* value over all mediators.

Second, we made sure that all exposure instruments were also genome-wide significant, ubiquitously available, passed Steiger filtering with the outcome and each mediator, and were clumped to a roughly independent set.

Finally, we combined the exposure and mediator instruments together and clumped them once more to ensure independence between the instrument sets.

## Results

### LiMA and I-LiMA decrease $\hat{\text{MP}}$ bias in simulations

We performed an extensive simulation study to examine how bias in $\hat{\text{MP}}$ is affected by the characteristics of the exposure, mediators, and outcome data. The study was primarily designed to mimic a mediation analysis where the exposure and outcome are complex traits (*n* = 300,000) and the mediators are molecular traits (*n* = 10,000), but we also explored various other realistic settings (Table S1; methods). We assumed the mediation model depicted in Figure 1A, generating all the necessary summary statistics to estimate MP within the mediation framework. We then compared $\hat{\text{MP}}$ estimates obtained from the classical MR+MVMR approach, LiMA, and I-LiMA (Figure 2).

The sample sizes underlying the exposure, mediator, and outcome summary effect estimates used in the mediation analysis—inversely proportional to the measurement errors—affect the $\hat{\text{MP}}$ bias, but each in a distinct way (Figure 2A). When the mediator sample size is small, the true MP tends to be underestimated. This was most pronounced in the conventional MR approach used as a baseline and is consistent with theoretical expectations.^16^ With sample size *n*_*X*_ = 300,000 for the exposure, *n*_*M*_ = 10,000 for the mediators, and *n*_*Y*_ = 300,000 for the outcome, the MR+MVMR model underestimated the true *MP* by as much as 51.6% (95% confidence interval [CI]: [49.3%,53.9%]) (Tables 1 and S2). LiMA reduced this bias by nearly 2-fold, to 31.3% ([27.4%,35.3%]). I-LiMA alleviated the bias even further, to 24.5% ([22.1%,27.0%]). As expected, the bias of all methods converges to zero as the measurement error in the mediators decreases (Figure 2A).

With a low exposure sample size, the bias in the MR+MVMR model is reversed—$\hat{\text{MP}}$ tends to be overestimated. This upward bias can be alleviated slightly by shrinking the effects of mediator instruments on the exposure to zero (Figure S3). However, in both likelihood methods, the level of measurement error in the exposure does not affect the amount of $\hat{\text{MP}}$ bias (Figure 2A). With a low outcome sample size, MP tends to be underestimated by each method, though the standard errors for $\hat{\text{MP}}$ are also higher.

The criteria for selecting mediators in the model also affect the bias in $\hat{\text{MP}}$ (Figure 2B). To assess the robustness of our method to the inclusion of null mediators, we considered *k* = 500 mediators, of which only 5% were non-zero, and varied the *p* value threshold for mediator selection (Figure 2B; methods). If the threshold was too stringent, contributing mediators were omitted from the model, which naturally led to a downward bias in $\hat{\text{MP}}$. If the threshold was too lenient, many null mediators were selected—this did not affect the MR+MVMR model (Z-test^31^ *p* > 0.05 for the $\hat{\text{MP}}$ difference between Bonferroni selection threshold of 0.05/*k* and 0.2, corresponding to downward biases of 51.4% and 49.2%, respectively) but significantly increased the downward bias in both LiMA (from 30.5% to 49.6%, *p* = 0.02) and I-LiMA (from 24.2% to 45.8%, *p* = 0.0002). The reason for this bias stems from the fact that the mediator selection procedure changes the distribution of the exposure-to-mediator and mediator-to-outcome causal effects to a spike-and-slab distribution (a mixture of a bivariate Gaussian and a null), which increases the variance of *ω*. In supplemental methods, using the delta method, we derive how the downward bias for (I-)LiMA depends on the stringency of the mediator selection. Although MR+MVMR appeared slightly less sensitive to null mediators, weak instrument bias could still cause it to behave erratically.^13^ These results suggest that less stringent filtering leads to downward bias due to including more null mediators, which is not counterbalanced by including more true mediators. Therefore, we recommend preemptive mediator filtering.

Exposure genetics together with the true total causal effect from the exposure to the outcome also influence $\hat{\text{MP}}$ bias (Figure 2C). Both low exposure heritability and a low total causal effect increase the downward bias in all methods. This results from insufficient power to identify non-zero mediators. When assuming an exposure heritability of 0.35, a total causal effect of 0.15, and 25 non-zero mediators, approximately 11 of these mediators were selected in the model after filtering with a strict Bonferroni threshold. Conversely, when the exposure heritability was set to 0.01 or the total causal effect to 0.05, only 1 or 2 mediators were selected on average. In the presence of missing mediators, the likelihood methods estimate MP more accurately than the MR baseline. However, all the methods benefit from a less stringent mediator selection threshold when tiny genetic effects are suspected. The downward bias of $\hat{\text{MP}}$ in the MR+MVMR model also increases with exposure polygenicity, which we approximate by the number of exposure instruments. For a fixed exposure heritability, this is inversely proportional to the effect size of the instruments—and thus proportional to weak exposure instrument bias. Consistent with Figure 2A, the likelihood methods are not affected by this phenomenon.

The bias in $\hat{\text{MP}}$ also tends to increase as the true MP grows, particularly for the conventional MR approach and LiMA (Figure 2D). In contrast, the bias in I-LiMA is less dependent on the true MP.

#### $\hat{\text{MP}}$ is unreliable for small total causal effects

Bias is an aggregated measure, representing the average difference between observations and truth. However, individual MP estimates can be wildly influenced by other model properties, such as the variability of the model’s predictions and its confidence in them. We quantified these properties using relevant metrics—variance, coverage, power, and T1E—across various simulation settings (Figure 3; Table S2).

**Figure 3:**
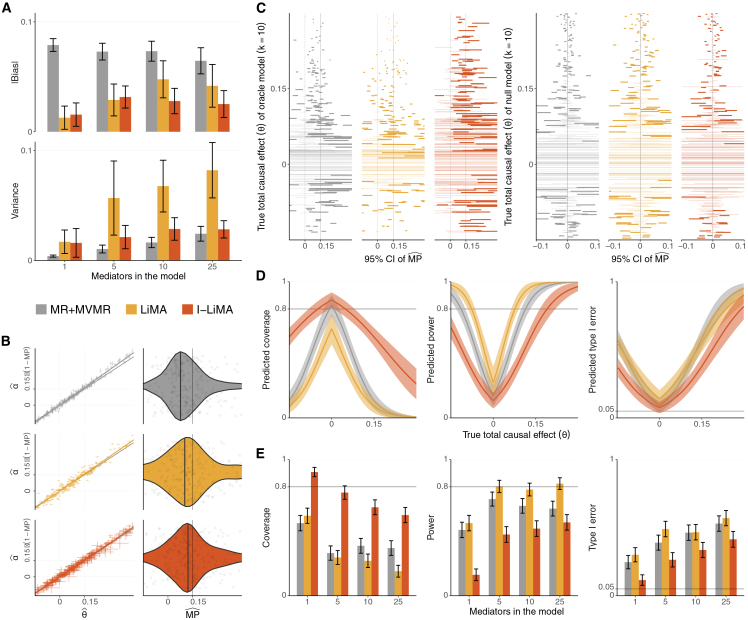
Performance metrics of the mediation analysis methods in the simulation study In each subplot, simulation scenarios consist of *i* = 1, …,300 simulations, and error bars always correspond to 95% confidence intervals (CIs). (A) Bias and variance in oracle simulation scenarios with *k* non-zero mediators for true MP = 0.15. (B) (Left) Direct and total causal effect estimates $\left({\hat{\alpha }}_{i},{\hat{\theta }}_{i}\right)$ with standard errors (corresponding to line lengths along each axis) for *k* = 10. Slopes of regression lines represent estimated direct effect proportions $1-\hat{\text{MP}}$. (Right) Distribution of estimates ${\hat{\text{MP}}}_{i}=1-{\hat{\alpha }}_{i}/{\hat{\theta }}_{i}$. Thin vertical lines indicate true *MP*, and bold vertical lines represent $\hat{\text{MP}}$ estimated by the regression slopes. (C) 95% CIs of ${\hat{\text{MP}}}_{i}$ for the oracle model (left) and the null model (right), both at *k* = 10 and ordered by the simulated true total causal effect *θ*_*i*_. Intervals that do not contain zero contribute to power and T1E, respectively, and are emphasized. The solid vertical lines represent the true expected *MP* (methods). (D) Predicted coverage, power, and T1E for different values of true total causal effect θ at *k* = 10. (E) Coverage and power in oracle simulation scenarios with *k* non-zero mediators for MP = 0.15 and T1E for simulation models with *k* null mediators for MP = 0. In both cases, simulations were done with the expected total causal effect E(*θ*) = 0.15.

Consider an oracle simulation scenario where variability from the mediator selection procedure is removed (all and only non-zero mediators are selected in the model). We already know that mediator selection affects model bias but not the relative ranking of the models (Figure 2B). The oracle scenario thus still demonstrates the familiar pattern—likelihood methods reduce $\hat{\text{MP}}$ bias compared to MR+MVMR—but highlights how this comes at a cost of increased estimator variability in LiMA (Figure 3A). This is akin to the well-known bias-variance trade-off in statistical machine learning—model complexity increases flexibility to match underlying data points (less bias) but is more attuned to subtle differences in said data (more variance). I-LiMA addresses this complexity by integrating out individual mediator effects.

Despite the relative differences in bias and variance, $\hat{\text{MP}}$ can take extreme values in individual simulation runs for all methods (Figure 3B, right). This is largely due to $\hat{\text{MP}}$ being a ratio estimator. When the total effect (denominator in the ratio) is estimated to be small, even minor differences in the direct effect (numerator) estimates can easily offset $\hat{\text{MP}}$—potentially pushing it outside the valid MP domain to negative values. However, this is not necessarily problematic and often does not lead to spurious results, as $\text{Var}\left(\hat{\text{MP}}\right)$ tends to be large when $\left|\hat{\theta }\right|$ is small (methods), keeping the null effect consistent with the 95% CI of $\hat{\text{MP}}$ (Figure 3C, right). At the same time, large variances reduce the power to detect non-zero MP values (Figure 3C, left)—a limitation that is exacerbated when the true |*θ*| is small (Figure 3D, middle), though not necessarily when the true MP is small (Table S2). In real applications, it can be advantageous to consider mediation only when the total causal effect is estimated to be substantial, which also helps reduce the multiple-testing burden. A threshold of 0.1 on a standardized scale performed well in our standard simulation settings but should be balanced with biological plausibility to avoid overlooking meaningful mediation with smaller total effects.

#### I-LiMA better controls the T1E rate at minor loss of power

While all methods experience reduced power when the total causal effect is small, our simulations revealed that this reduction gradually increases from LiMA to MR+MVMR to I-LiMA (Figure 3E, middle; Table 1). In the default simulation settings with a true MP = 0.15, the statistical power of LiMA to correctly reject the null was 81.4% [80.2%,82.7%], whereas MR+MVMR achieved 63.1% [61.5%,64.7%]. I-LiMA’s power was at 53.1% [51.5%,54.8%].

However, power values must be interpreted with caution when T1E is not sufficiently controlled. The conventional MR-based approach is susceptible to bias, while LiMA exhibits higher variance. Both methods (unlike I-LiMA) tend to be overly confident in their estimates, as reflected by small standard errors (Figure 3B, left), consequently affecting T1E control. In no-mediation settings (MP = 0) with E(*θ*) = 0.15, the T1E for MR+MVMR in a model with a single null mediator reached 24.7% [19.8%,29.6%] and was even higher for LiMA, at 30.1% [24.9%,35.3%]. In contrast, I-LiMA achieved a several-fold reduction in T1E at 11.5% [7.6%,15.4%] (Figure 3E, right). While T1E increased across all methods with each additional null mediator included in the model—and all methods struggle to control the T1E rate when the true total causal effect is large (Figure 3D, left)—I-LiMA consistently maintained 12%–13% better error control than the standard MR-based approach.

The bias-variance-confidence dynamics also translate to low coverage for both the MR-based methodology and LiMA (Figures 3C–3E, left; Table 1). In the default simulation settings, the 95% CI of $\hat{\text{MP}}$ estimated by MR+MVMR contained the true MP in only 38.2% [36.6%,39.8%] of the cases. While LiMA’s coverage was slightly lower at 17.8% [16.6%,19.1%], I-LiMA reached 63.2% [61.6%,64.8%].

The relationship between the methods in terms of bias, variance, coverage, T1E, and power remains consistent across other simulation scenarios with different parameter configurations (Figures 3A–3E and S4; Table S2).

#### I-LiMA is computationally efficient

The computational complexity of mediation methods depends on the number of mediators (*k*) and instruments (*m* and *l*) in the model (Table 1; methods). Additionally, the runtime of LiMA and I-LiMA is influenced by the number of iterations required for convergence, which depends on the number of parameters to estimate, the shape of the objective function, and other factors.

Pre-filtering mediators is advantageous not only for obtaining more robust $\hat{\text{MP}}$ estimates (Figure 2B) but also for reducing computational burden (Figure 2E). Without pre-filtering, running LiMA can become impractical, as each additional mediator introduces two new parameters to estimate (Table 1). On an x86_64 Linux system using a single core of an AMD EPYC 7443 2.85 GHz CPU, our R implementation of LiMA had a median runtime of 1.02 s for a mediation model with 15 mediators. Since I-LiMA always estimates a fixed set of 4 parameters, it is computationally efficient, requiring just 0.13 s in the same settings (Figure 2E).

### Omics traits mediate the causal effects from epidemiological risk factors to cardiovascular outcomes

**Figure 4:**
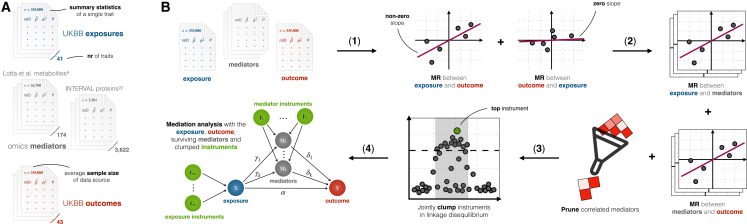
Overview of the data and methods used in the mediation study (A) Summary statistics used in the study. Exposure and outcome data (altogether 43 traits, though only 41 were used as exposures) originate from the UK Biobank (UKBB) with sample sizes around *n* ≈ 335,000. As mediators, we used metabolite data from Lotta et al.^9^ (*n* ≈ 26,700) and protein data from the INTERVAL study^22^ (*n* ≈ 3,301). Available mediators ranged from hundreds, in the case of metabolites, to thousands, in the case of proteins, with sample sizes up to two orders of magnitude smaller than those of UKBB complex traits. This (discrepancy in) measurement error means that MR-based mediation analysis would be unreliable. (B) Analysis pipeline for estimating omics-wide mediation of the causal effects between complex traits. Starting from an exposure, a set of mediators, and an outcome, we first test the presence of a unidirectional causal effect from the exposure to the outcome, with no reverse effect. If such a causal effect exists, we identify mediators with non-zero mediation of this causal effect. If any such mediators are correlated, we prune them. Then, we perform clumping to ensure that exposure and mediator instruments are uncorrelated. Finally, we apply the mediation methods on the data.

We applied the mediation analysis methods to examine the influence of 174 metabolites from Lotta et al.^9^ and 3,622 proteins from the INTERVAL study^22^ on the causal relationship between 43 epidemiological risk factors and cardiovascular outcomes measured in the UKBB (Figure 4A; Table S8). The relative mismatch in the sample sizes of protein (*n* ≈ 3,301) and metabolite (*n* ≈ 26,700) mediators compared to the complex traits used as exposures and outcomes (*n* ≈ 335,000) can introduce bias in MP estimates. Furthermore, quantifying omics-wide mediation is computationally challenging, as thousands of traits may need to be considered in any given mediation model. To reduce $\hat{\text{MP}}$ bias as well as computational burden, we applied the LiMA methods following the pre-filtering principles observed to be efficient in the simulation study (Figure 4B).

First, we considered only exposure-outcome pairs with a strong total causal effect *θ* (MR *p* ≤ 5 × 10^−8^) and no reverse causation—altogether, 178 pairs (Figure S5; Table S3). Second, we filtered out null mediators from the model by selecting only those for which both the exposure → mediator and mediator → outcome causal effects were estimated to be non-zero (MR *p* ≤ 0.05). Third, we applied additional LD-based filtering to prune any correlated mediators. For each selected exposure-mediator-outcome tuple, we identified genetic instruments for both the exposure and mediators, jointly clumped the instruments based on LD (methods), and finally performed the mediation analyses. While our simulations showed optimal results using a Bonferroni-corrected threshold on exposure → mediator effects, applying the same criterion to real data yielded too few mediators for meaningful analysis after pruning—often leaving only a single mediator. This is largely due to small sample bias and the low statistical power of MR, which strongly depends on the outcome (in this case, the mediator) sample size.^19^^,^^32^ To address this and balance mediator inclusion but still control the false positive rate, we applied a nominal threshold to both directions, including mediator → outcome effects.

#### Glutamate as a mediator of adiposity-driven systemic changes

**Figure 5:**
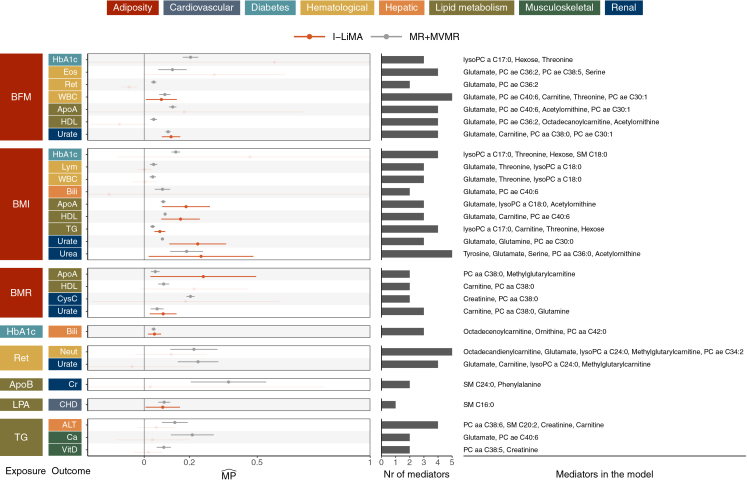
Proportion of causal effects from cardiometabolic risk factors to cardiovascular outcomes mediated by metabolites from the Lotta et al. study Exposures and outcomes are colored based on their annotation (top legend). In the forest plot, line lengths correspond to 95% confidence intervals. The bars in the middle show the number of mediators selected in the models. The latter are depicted on the right, ordered based on MR IVW *p* values of the causal effects from the exposure to the mediators. Results are shown, for both MR+MVMR and I-LiMA, if $\hat{\text{MP}}$ from either method was correctly defined within [0,1] and was significant after Bonferroni correction. Among those, nominally significant results are emphasized, while others are shown transparently. ALT, alanine aminotransferase; ApoA, apolipoprotein A; ApoB, apolipoprotein B; BFM, body fat mass; Bili, total bilirubin; BMI, body mass index; BMR, basal metabolism rate; Ca, calcium; CHD, chronic ischemic heart disease; Cr, creatinine; CysC, cystatin C; Eos, eosinophil count; HbA1c, glycated hemoglobin; HDL, high-density lipoprotein cholesterol; LPA, lipoprotein A; Neut, neutrophil count; Ret, reticulocyte count; TG, triglycerides; Urate, serum urate; Urea, urea; VitD, vitamin D; WBC, white blood cell count.

In the metabolite-focused analysis, we identified significant mediation of causal relationships (both MR+MVMR and I-LiMA $\hat{\text{MP}}$ *p* ≤ 0.05) between adiposity-related traits—body mass index (BMI), body fat mass (BFM), and basal metabolic rate—and traits involved in inflammation (white blood cell [WBC] count and C-reactive protein), lipid metabolism (high-density lipoprotein [HDL] cholesterol, triglycerides, and apolipoprotein A [ApoA]), and renal function (urate and urea) (Figure 5). In terms of pairwise causal relationships, these results corroborate findings from existing literature,^33^^,^^34^^,^^35^^,^^36^ but our analysis provides additional insights into the metabolic pathways involved.

Glutamate emerged as a key metabolite mediating adiposity-driven causal effects. Its role was observed in metabolite mediation of causal pathways linking BFM to WBC (I-LiMA $\hat{\text{MP}}=0.076$, *p* = 0.03) and urate ($\hat{\text{MP}}=0.12$, *p* = 1.17 × 10^−8^), as well as BMI to ApoA ($\hat{\text{MP}}=0.19$, *p* = 0.0006), HDL cholesterol ($\hat{\text{MP}}=0.16$, *p* = 0.0002), urate ($\hat{\text{MP}}=0.24$, *p* = 0.0002), and urea ($\hat{\text{MP}}=0.25$, *p* = 0.03) (Figure 5). Specifically, glutamate levels increased with adiposity and were associated with elevated WBC count (indicative of inflammation), higher urate levels, and reduced HDL and ApoA (Table S4).

Elevated glutamate levels have previously been implicated in oxidative stress, inflammation, and metabolic disorders,^37^^,^^38^^,^^39^ supporting its role as a mediator in adiposity-driven phenotypic changes. Glutamate’s role as a precursor to glutathione^40^—a major antioxidant involved in reducing inflammation^41^^,^^42^—may underpin its observed mediatory effect on decreased ApoA levels. Additionally, dietary glutamate supplementation has been shown to reduce HDL cholesterol^43^ (and, consequently, ApoA, its primary protein constituent) in humans. Conversely, glutamate’s metabolism into purines, which degrade into urate,^40^ aligns with its observed contribution to elevated urate levels. Together, these findings underscore the intricate metabolic interplay linking glutamate to adiposity-related systemic changes.

#### Metabolites as intermediaries of glycemic control-oxidative stress dynamics

Consistent with other studies, we also observed an inverse association between glycated hemoglobin (HbA1c) and bilirubin levels (MR IVW $\hat{\theta }=-0.23$, *p* = 4.32 × 10^−83^). The I-LiMA estimate suggests that a proportion of this causal effect is mediated through metabolites, with $\hat{\text{MP}}=0.046$ (*p* = 0.002). HbA1c measurements reflect average blood glucose concentrations over 2–3 months,^44^ while bilirubin serves as a potent endogenous antioxidant.^42^^,^^45^ Elevated HbA1c, potentially indicative of chronic hyperglycemia,^44^ is associated with increased oxidative stress and tissue damage,^46^ which may disrupt bilirubin production, metabolism, and clearance. Our results suggest that metabolites may play a significant role in the interplay between glycemic control and antioxidant pathways, particularly through octadecenoylcarnitine, a long-chain acylcarnitine involved in mitochondrial energy production, and ornithine, an amino acid of the urea cycle affecting nitrogen metabolism and nitric oxide production (Figure 5)—both linked to processes that regulate reactive oxygen species and oxidative stress.^47^^,^^48^

#### Protein mediation aligns with metabolite findings

In addition to our metabolite analysis, we explored the potential mediatory roles of INTERVAL proteins^22^ in the causal relationships between complex traits. We observed overlap in significant protein mediation (identified by both MR+MVMR and I-LiMA) with findings from the metabolite mediation study, including mediation of BMI → HDL (I-LiMA $\hat{\text{MP}}=0.029$, *p* = 9.73 × 10^−5^) and HbA1c → Bili (I-LiMA $\hat{\text{MP}}=0.015$, *p* = 0.004) causal effects (Figure S6A). The interleukin-6 receptor (IL-6R), a pro-inflammatory cytokine and drug target of immunological conditions, was identified as a mediator of the latter relationship, suggesting that both oxidative stress and inflammation link glycemic control with bilirubin metabolism.^49^

Candidate protein mediators of the BMI → HDL relationship include TMEM132D, LILRA5, GSTA1, and LCT. While these proteins have diverse annotated functions, they converge on processes relevant to metabolic regulation in obesity. TMEM132D has been associated with anxiety phenotypes,^50^ aligning with neuroendocrine pathways that influence adiposity and lipid homeostasis.^51^ LILRA5 stimulates innate immune responses via cytokine signaling, reflecting inflammatory mechanisms that can suppress HDL in obesity.^52^^,^^53^ GSTA1 reduces hepatic triglyceride accumulation and mitigates steatosis, suggesting a potential interplay with HDL.^54^^,^^55^ Finally, LCT encodes lactase, which hydrolyzes lactose into absorbable sugars^56^ and may indirectly influence lipid levels through modulation of nutrient absorption and hepatic metabolic flux.

We also observed putative protein mediation in the causal relationships of low-density lipoprotein cholesterol with coronary heart disease (I-LiMA $\hat{\text{MP}}=0.02$, *p* = 5.28 × 10^−5^) and triglycerides with calcium levels (I-LiMA $\hat{\text{MP}}=0.19$, *p* = 0.02). However, these associations did not remain robust under sensitivity analyses in which mediators included in the model were varied through significance thresholds (Figure S6B). Complete results of the protein-focused analysis are provided in Table S4.

### I-LiMA corrects for downward $\hat{\text{MP}}$ bias and increases T1E control on real data, consistent with the simulation study

**Figure 6:**
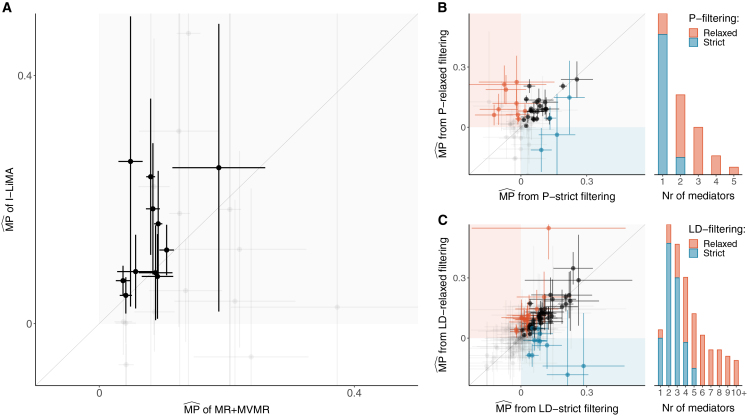
Comparisons of $\hat{\text{MP}}$ obtained using different methods and strategies of mediator filtering In the scatterplots, each point corresponds to a mediation analysis with UKBB exposure-outcome and Lotta et al.^9^ metabolite mediators. Line lengths along each axis correspond to 95% confidence intervals. Black points indicate that metabolites are nominally significant along both axes; red and blue points indicate significance only along the *y* or *x* axis, respectively. (A) Differences in $\hat{\text{MP}}$ obtained by MR+MVMR and I-LiMA. (B) The influence of MR IVW *p* value-based mediator selection on $\hat{\text{MP}}$. Relaxed (default) filtering assumes exposure → mediator and mediator → outcome causal effects to be nominally significant, and strict filtering assumes Bonferroni significance. (C) The influence of LD-correlation-based mediator selection on $\hat{\text{MP}}$. Strict (default) filtering assumes that the correlation among any two mediators is at most 0.1, and relaxed filtering uses the threshold 0.5. For both (B) and (C), the histograms depict the distribution of the number of metabolites selected in the mediation analyses with each filtering strategy.

In the analysis with real data, we do not know the true MPs of the total causal effects between complex traits attributed to our metabolite and protein mediators. However, we can interpret the observed results on real data based on expectations from the synthetic data in the simulation study (Figure 6).

In our analysis with metabolite mediators, MR+MVMR identified 28 significant $\hat{\text{MP}}$ findings, whereas I-LiMA identified only 11 significant results (Figure 5), all of which were also detected by MR+MVMR. Our simulation results showed that I-LiMA offers better T1E control with minimal loss of power compared to MR+MVMR; hence, we speculate that most of the extra hits observed here are likely false positives. Furthermore, among the 11 findings supported by both methods, I-LiMA reported higher $\hat{\text{MP}}$ values in 9 cases (*p* = 0.03) (Figure 6A), which is in line with the milder downward bias in $\hat{\text{MP}}$ observed in our simulations for I-LiMA.

#### Mediator filtering reduces computational load with negligible impact on $\hat{\text{MP}}$, also consistent with the simulation study

We considered two distinct strategies for selecting mediators in the model. First, mediators were selected based on the potential of their individual contribution to the overall $\hat{\text{MP}}$, determined using univariate MR causal effect estimates of exposure → mediator and mediator → outcome. A mediator was included if both MR *p* values satisfied a pre-defined threshold. A stringent threshold requiring Bonferroni significance rarely left more than a single mediator in any of the metabolite models and never more than two (Figure 6B). In contrast, a more relaxed threshold—requiring nominal significance—selected up to five metabolites. Based on our simulations, we know that mediator filtering is beneficial for weeding out null mediators, but too stringent a threshold can downward bias $\hat{\text{MP}}$ by excluding important mediators. Our real data analysis supports this, as $\hat{\text{MP}}$ tended to be higher with relaxed filtering, resulting in more findings compared to strict filtering (9 unique hits compared to 4), even though the MP estimates themselves were generally in agreement ($\hat{r}=0.65$, Figure 6B).

Second, we pruned mediators based on LD correlation. Here, we defined relaxed filtering as a correlation threshold of 0.5, whereas strict filtering allowed at most a 0.1 correlation between any two mediators (methods). Allowing for higher correlation among mediators resulted in a larger number of mediators included in the model (up to 10+, compared to at most 5 with strict filtering) but had a negligible effect on the $\hat{\text{MP}}$ as estimated by I-LiMA (Figure 6C). This outcome is also consistent with our simulation study.

Applying mediator filtering is beneficial for computational efficiency, as it reduces the data size. Taking into account insights from the simulation study, we recommend a more relaxed filtering approach for individual mediator contributions to avoid downward bias in $\hat{\text{MP}}$, while correlated mediators can be filtered more stringently.

## Discussion

Performing MR has become widespread due to easily accessible software (e.g., the R packages MendelianRandomization^57^ and TwoSampleMR^1^), as well as summary statistic databases such as MR-Base.^1^^,^^58^ Mediation analysis methods incorporate more information than their MR counterparts, allowing for the distinction between direct and indirect (mediated) effects. Thus, mediation methods represent a step forward in building causal networks and uncovering complex relationships within biological systems. At the same time, the added layer of complexity has introduced challenges regarding the credibility and accuracy of mediation results.^13^^,^^16^ Applications have also been limited due to the lack of easy-to-use and reliable software for conducting such analyses. We have addressed these issues by (1) demonstrating the necessary decision-making involved in conducting mediation analyses; (2) outlining the limitations of the baseline MR+MVMR methodology; (3) presenting methods that mitigate biases inherent in many mediation applications; (4) elucidating how MP estimation is influenced by factors such as the sample sizes of exposure-mediator-outcome studies, mediator selection in the model, the total causal effect, and other parameters; and (5) providing accessible software implementations for conducting mediation analyses.

The conventional MR-based approach for estimating MP in two steps—using univariable MR to estimate the total causal effect *θ* and then MVMR to estimate the direct effect *α*—is susceptible to severe biases. As any MR (uni- or multivariable) can be treated as an OLS regression of outcome effects on exposure effects, the OLS assumptions apply, including that exposure effects as regressors should be measured without error (Figure 1B). The smaller the sample size of the exposures, the larger the variance (measurement error) of the exposure effects acting as regressors in the model, and the greater the potential bias. While this is less of a concern when the effects are estimated from well-powered GWASs with large samples, classical MR estimators are always biased in finite samples, even when all the MR assumptions are satisfied.^19^ The primary contributor to $\hat{\text{MP}}$ bias is exactly the regression dilution bias induced by measurement error in the exposure effects, often referred to as weak instrument bias in the MR field. The bias in univariable two-sample MR is toward the null effect,^59^ making $\hat{\theta }$ a conservative estimate, although sample overlap and winner’s curse in instrument selection can somewhat skew the bias toward the observed exposure-outcome correlation (Table S5).^60^ In MVMR, the bias can occur in any direction, depending on the size, direction, measurement accuracy, correlation structure of the exposure and mediator effects, and variability in the risk factor effect estimates.^16^^,^^60^ The bias in $\hat{\text{MP}}$, as a ratio of MVMR and MR estimates, can thus be further amplified. When mediators have more measurement error than the exposure—such as when the mediator study has a smaller sample size than the exposure study, which is often the case with currently available datasets—the indirect effect is underestimated, and vice versa (Table S6).

The theoretical properties and consequences of measurement error in MVMR have been tackled previously.^16^ Here, we generalize the treatment to potentially hundreds of mediators, with a focus on molecular mediation between complex traits across the phenome. Our proposed methods are based on likelihood function maximization. We performed extensive simulation analyses in realistic settings to demonstrate that our LiMA and I-LiMA approaches significantly reduce bias in $\hat{\text{MP}}$ compared to MR+MVMR (Figure 2). I-LiMA also offers better control of T1E and achieves higher coverage of the true MP, with only a minor loss in statistical power (Tables 1 and S2). The latter is not a limitation of I-LiMA but rather a reflection of its more appropriate T1E control. While caution is warranted when interpreting the power values, as none of the methods fully controlled T1E, I-LiMA consistently demonstrated better performance relative to the other methods. The general improvements from our proposed methods stem from directly handling the noise in all estimates, eliminating the need for the assumption of no measurement error. Furthermore, by integrating out nuisance parameters, I-LiMA improves both computational efficiency and estimator accuracy. Importantly, I-LiMA’s performance across all metrics remains robust even relative to alternative MVMR methods that relax the no-measurement error assumption^16^; such methods can reduce $\hat{\text{MP}}$ bias in the standard MR-based mediation approach but at the cost of inflated standard errors (Figures S7 and S8).

We applied I-LiMA to investigate metabolite and protein mediation of the causal effects from epidemiological risk factors to cardiovascular outcomes. Our analysis highlighted biologically plausible mechanisms, including glutamate in adiposity-driven inflammation, metabolite-mediated regulation of the HbA1c-bilirubin axis via oxidative stress, and protein mediation of BMI → HDL through metabolic regulation in obesity. To strengthen inference, we combined evidence from both MR+MVMR and I-LiMA. Although theory and simulations show that the former exhibits poorer T1E control and yields more biased MP estimates when exposure and mediator sample sizes differ, its simplicity and grounding in MR make it an attractive baseline for mediation analysis. By correcting the bias and improving T1E control, I-LiMA facilitates prioritization and interpretation of findings. Consistent with MR best practices, we advocate triangulation of evidence across complementary methods based on distinct assumptions.

Although our likelihood-based methods improve $\hat{\text{MP}}$ estimation, simulations suggest that some residual bias remains (Figure 2). Additional factors beyond measurement error could contribute to this persisting bias. In applied settings, instrument selection may lead to the winner’s curse, though we also observed bias in oracle simulation scenarios where the winner’s curse was eliminated (Figure 3A). To further reduce $\hat{\text{MP}}$ bias in mediation analyses, in addition to using our likelihood-based methodology, careful attention should be given to the selection of mediators in the model. The selection of mediators is often based on statistical evidence for an exposure-to-mediator (and mediator-to-outcome) link, and this process can introduce biases: too stringent filters fail to include non-zero mediators, leading to underestimation of $\hat{\text{MP}}$, as does the inclusion of too many null mediators (Figure 2B). Optimal feature selection was beyond the scope of this paper, as it is orthogonal to benchmarking biases in mediation effects given a set of mediators. However, it would complement any mediation analysis and represents an important direction for future work. For example, methods such as MR-BMA (Mendelian Randomization Bayesian Model Averaging) could be leveraged to prioritize true mediators among a set of correlated candidates.^61^ Guided by the simulation study, we applied mild filtering in the omics analysis to narrow large pools of metabolites and proteins to promising subsets. While a more comprehensive mediator selection strategy might improve robustness further, $\hat{\text{MP}}$ bias is not fully controlled even with an optimal set of mediators (Figure 3A). Importantly, however, relative differences between the methods persist in the oracle scenario, with I-LiMA outperforming the competition.

Our likelihood-based methods rely on some assumptions that need to be considered in applications, even if they can typically be met under realistic scenarios. We allow for horizontal pleiotropy in the instruments, provided the heterogeneity in the summary effect estimates is independent of instrument strength (InSIDE assumption) and averages to zero. This is a common assumption, which is also adhered to by the standard MR IVW estimator for permitting valid inference. Just like for any other method, violations of the InSIDE assumption—such as when some mediators (shared pathways) are missed or not accounted for in the model (Figure 2B)—can introduce biases in the MP estimator.

Although LiMA and I-LiMA allow explicit modeling of pleiotropy, they provide robust $\hat{\text{MP}}$ estimates even in the unlikely scenario where horizontal pleiotropy is entirely absent (Figure S9), with the added benefit of improved computational efficiency due to fewer parameters to optimize. In the derivations of our methods, we also assume that there is no sample overlap between mediator and outcome studies and that mediator effects have been estimated from the same sample (i.e., the underlying sample size *n*_*M*_ is roughly the same across all mediators). These assumptions are likely satisfied in studies investigating molecular mediation between complex traits. No sample overlap is also generally assumed in two-sample MR scans, and its violation does not lead to noticeable bias.^60^

In I-LiMA, we further assume that mediators are independent (Σ = I_*k*_), which is a strong assumption that might not hold in practice. While this assumption provides computational advantages, its violation could compromise T1E control and mediation effect estimation. That said, we have shown in simulations that I-LiMA is relatively robust to the violation of this assumption, outperforming MR+MVMR in terms of $\hat{\text{MP}}$ bias even when mediators are highly correlated (Figure S1). Furthermore, mediators can be orthogonalized, even at the summary statistic level, through an appropriate transformation, such as principal-component analysis (supplemental methods). The principal components are naturally uncorrelated and could be used to replace the original mediators. Finally, mediators can be easily pruned to meet the assumption of independence. This is even recommended, as all the methods covered in this paper benefit from prior mediator filtering in terms of the assessed metrics: bias, variance, coverage, power, T1E, and runtime (Figures 2B, 2E, and 3E; Table S2). We leave the formal modeling of mediator correlations for future research.

The mediation proportion $\hat{\text{MP}}$ can be an unreliable measure of mediation. As a ratio of the indirect effect to the total causal effect, it should have a lower bound of 0 and an upper bound of 1. However, these bounds cannot always be guaranteed in finite sample estimation or when the assumptions of the mediation framework and methods are violated. Values of $\hat{\text{MP}}$ can be particularly extreme when the total causal effect (the denominator in the ratio) is close to zero (or the coefficient of variation of its estimator is high), as even chance sampling variation could substantially distort the estimate.^62^^,^^63^ In such cases, the delta method yields inflated variance estimates (methods), and statistical power to reject the null is severely limited (Figures 3C and 3D). Alternative approaches to the delta method in order to obtain the estimator variance, such as the inverse of the Fisher information matrix or via the likelihood ratio test, may be more robust in certain cases. Still, mediation investigations are typically conducted once the total causal effect has been well established,^13^^,^^16^ when the delta method is expected to be robust. A more critical limitation is that MP is undefined when the signs of direct and indirect causal effects are opposite, even if feedback mechanisms between traits are realistic and feasible. Therefore, we also recommend considering the direct and indirect causal effects separately when interpreting results from mediation analyses. This also allows the identification of underlying mediators once non-zero total mediation is established. Since I-LiMA estimates only the overall MP, additional tools are required for this purpose; in our analysis, we used MR+MVMR.

To conclude, we have presented advanced mediation methods, termed LiMA and I-LiMA, which can model the impact of hundreds of traits—potentially entire omics layers—as mediators simultaneously, thus bringing us closer to building comprehensive causal networks. Compared to conventional MR-based methodologies, our proposed methods reduce bias, increase coverage, and better control T1E in the mediation proportion ($\hat{\text{MP}}$) by jointly modeling the variability of each estimate used in the mediation model. Our LiMA methods require only summary statistics and, along with MR+MVMR, are implemented in R and made available under the GPLv3 license.

## Data and code availability

The software to run the analyses includes Nextflow workflows and R scripts that have been released under the GPLv3 license, available on GitHub: https://github.com/kaidolepik/LiMA.

## Acknowledgments

This work was supported by funding from the Department of Computational Biology of the 10.13039/501100006390University of Lausanne and the University Center for Primary Care and Public Health (Unisanté) of Lausanne, Switzerland. Z.K. was funded by the 10.13039/501100001711Swiss National Science Foundation (310030-189147). Computations were carried out in part in the high-performance computing clusters JURA and Urblauna of the University of Lausanne. We thank the participants of the CoLaus Study whose RNA-seq data we used to inform gene expression correlations, and we are grateful to Sven Bergmann for making these data available to us.

## Author contributions

Z.K. conceived the study. K.L. and Z.K. designed the study and derived the methods. S.E.O. contributed to the derivations. K.L. implemented the methods and performed the statistical analyses. M.C.S. contributed to the statistical analyses. A.v.d.G., C.A., K.L., and M.C.S. biologically interpreted the results. K.L. drafted the first version of the manuscript. Z.K. supervised the study. All the authors read, approved, and provided feedback on the final manuscript.

## Declaration of interests

The authors declare no competing interests.

## Web resources

Metabolites from Lotta et al., GWAS Catalog, https://www.ebi.ac.uk/gwas/publications/33414548
Neale Lab, http://www.nealelab.is/uk-biobank
Omics Science, https://omicscience.org
Plasma pQTLs in INTERVAL cohort, https://ega-archive.org/studies/EGAS00001002555
UK10K, https://www.uk10k.org/data_access.html

## Supplemental information

Document S1. Figures S1–S12, Tables S1, S2, and S5–S9, and supplemental methods

Table S3. MR IVW pairwise causal effect estimates A–E have varying types of exposure and outcome data.

Table S4. Estimates of the proportion of causal effect from risk factors to cardiovascular outcomes mediated by metabolites and proteins A–D compare different methods, mediator filtering strategies, and mediator types.

Document S2. Article plus supplemental information
