# Supplementary material for: LiMA: Robust inference of molecular mediation from summary statistics
Source: Am J Hum Genet. 2026 Jan 8;113(1):202–20. doi: 10.1016/j.ajhg.2025.12.005 (PMC12824626; doi:10.1016/j.ajhg.2025.12.005)
Supplement: Document S1. Figures S1–S12, Tables S1, S2, and S5–S9, and supplemental methods [file mmc1.pdf]

The American Journal of Human Genetics, Volume *113*

Supplemental information

LiMA: Robust inference of molecular mediation from summary statistics

Kaido Lepik, Chiara Auwerx, Marie C. Sadler, Adriaan van der Graaf, Sven Erik Ojavee, and Zoltán Kutalik

Contents

Supplemental Figures

S1 The influence of mediator correlation matrix Σ on simulation results . . . .
S2 The performance of different flavours of LiMA in the simulation study . . . .
S3 The performance of different flavours of MR framework in the simulation study . . . .
S4 Comparison of T1E control and statistical power of methods . . . .
S5 Mendelian randomization results between UKBB complex traits . . . .
S6 Proportion of causal effect mediated by INTERVAL proteins . . . .
S7 The performance of the method by Zhu et al. 2022 in the default simulation settings .
S8 The performance of the method by Zhu et al. 2022 in the basic simulation settings . .
S9 The influence of pleiotropy on simulation results . . . .
S10 The performance of different flavours of I-LiMA in the simulation study . . . .
S11 Distribution of mediator heritabilities $h^2_{M_j}$ . . . .

Supplemental Tables

S1 Default values of parameters used in the simulation study . . . .
S2 Bias, variance, coverage and power in estimating MP in the simulation study . . . . . .
S3 MR IVW estimates of pairwise causal effects . . . .
S3A UKBB exposures, UKBB outcomes . . . .
S3B UKBB exposures, Lotta et al. 2021 outcomes . . . .
S3C UKBB exposures, INTERVAL outcomes . . . .
S3D Lotta exposures, UKBB outcomes . . . .
S3E INTERVAL exposures, UKBB outcomes . . . .
S4 Estimates of the proportion of causal effect mediated . . . .
S4A Lotta et al. 2021 mediators, default . . . .
S4B Lotta et al. 2021 mediators with strict P-value based filtering . . . .
S4C Lotta et al. 2021 mediators with relaxed LD based filtering . . . .
S4D INTERVAL mediators, default . . . .
S5 Bias, variance, coverage and power in estimating θ in the simulation study . . . .
S6 Bias, variance, coverage and power in estimating α in the simulation study . . . .
S7 Failure characteristics of likelihood function optimization in the simulation study . . .
S8 UK Biobank complex traits used in the mediation study . . . .
S9 Distribution of the number of mediator instruments l_j . . . .

Supplemental Methods

Maximum likelihood method . . . .
Integrated likelihood method . . . .
Prior distribution . . . .
Marginal distribution . . . .
Optimizing the likelihood . . . .

Simplifying the likelihood function for $\hat{c}$ . . . .
Simplifying the likelihood function for $\begin{pmatrix} \hat{b} \\ \text{vec}(\hat{C}) \end{pmatrix}$ . . . .
Determining the variance components σ_γ^2 and σ_δ^2 . . . .
Relaxing the assumption of uncorrelated mediators . . . .
Impact of mediator selection on (I-)LiMA bias . . . .
References . . . .

Supplemental Figures

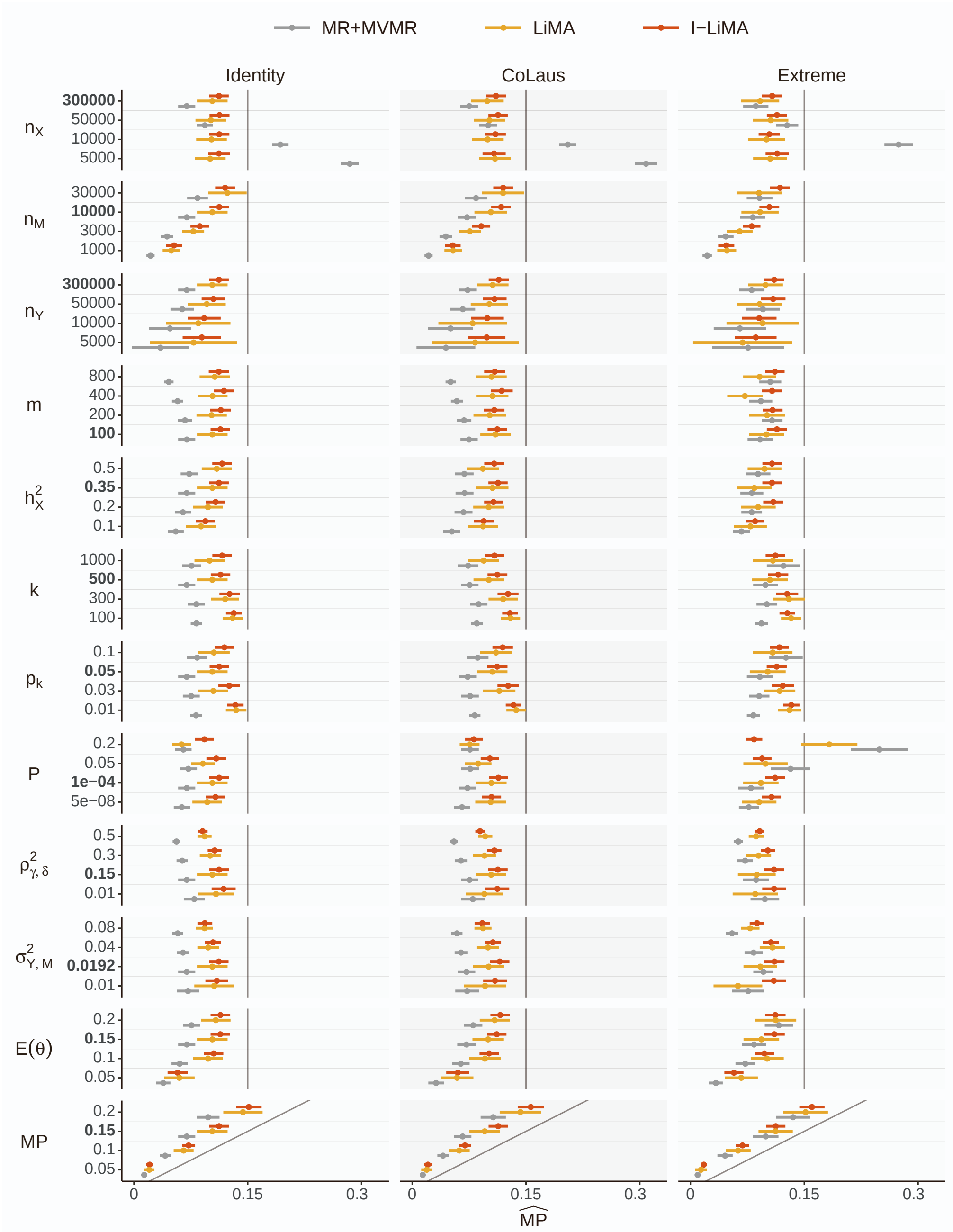

Fig. S1 | Simulation results for all parameter and mediator correlation matrix Σ combinations. For each parameter, the other parameters have been held fixed to the values in bold. In the main text, we reported the simulation results using Σ based on the CoLaus gene expression correlations (shaded). Error bars correspond to the 95% confidence interval. The true MP is displayed by the grey vertical line (diagonal in case of MP).

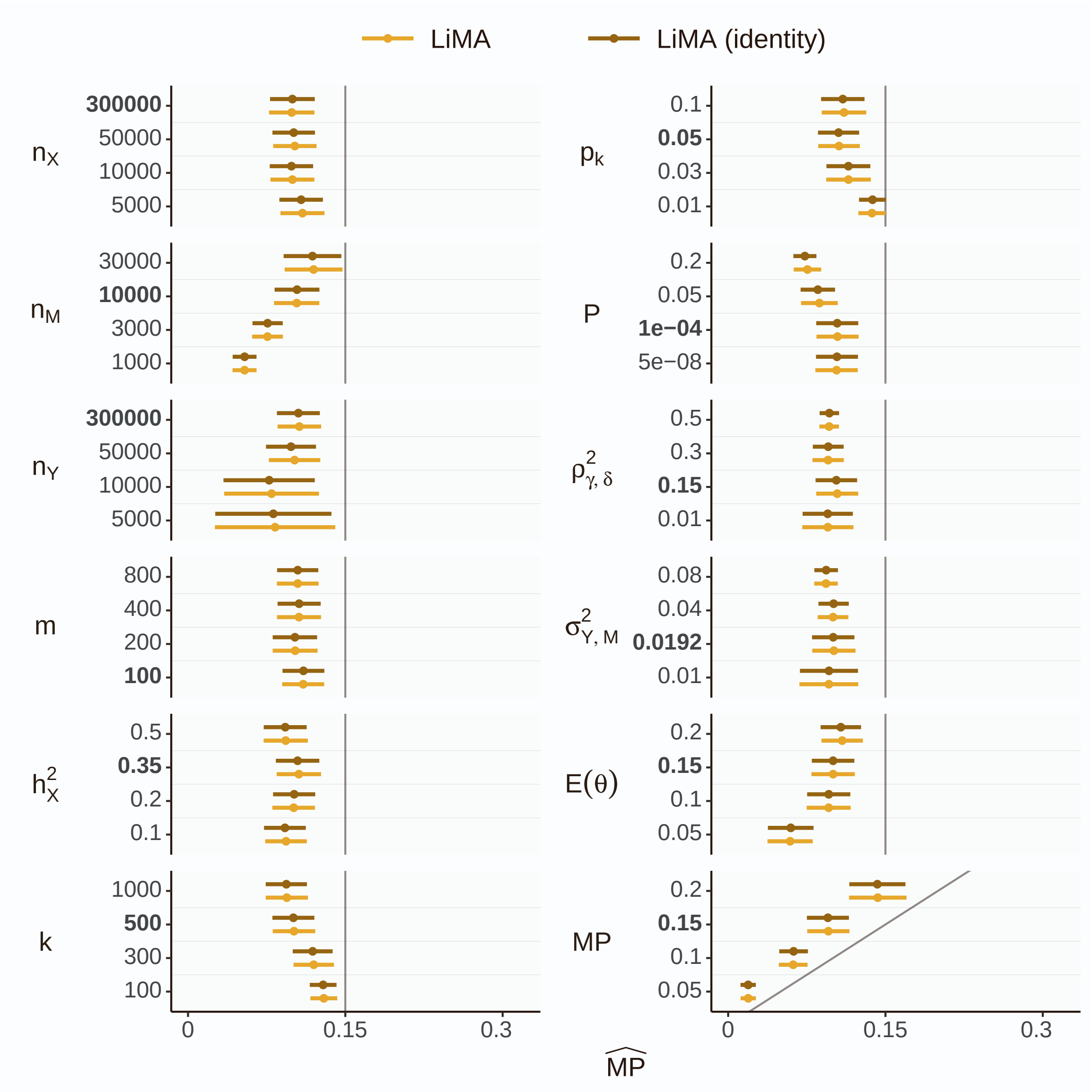

Fig. S2 | The influence of mediator correlation matrix on LiMA performance in the simulation study. The original LiMA estimates Σ based on the mediator instrument effect matrix $\widehat{\mathbf{B}}$ whereas LiMA (identity) takes $\widehat{\Sigma} = \mathrm{I}$. Data generation procedure assumed Σ based on the CoLaus gene expression correlations. Error bars correspond to the 95% confidence interval. The true MP is displayed by the grey vertical line (diagonal in case of MP).

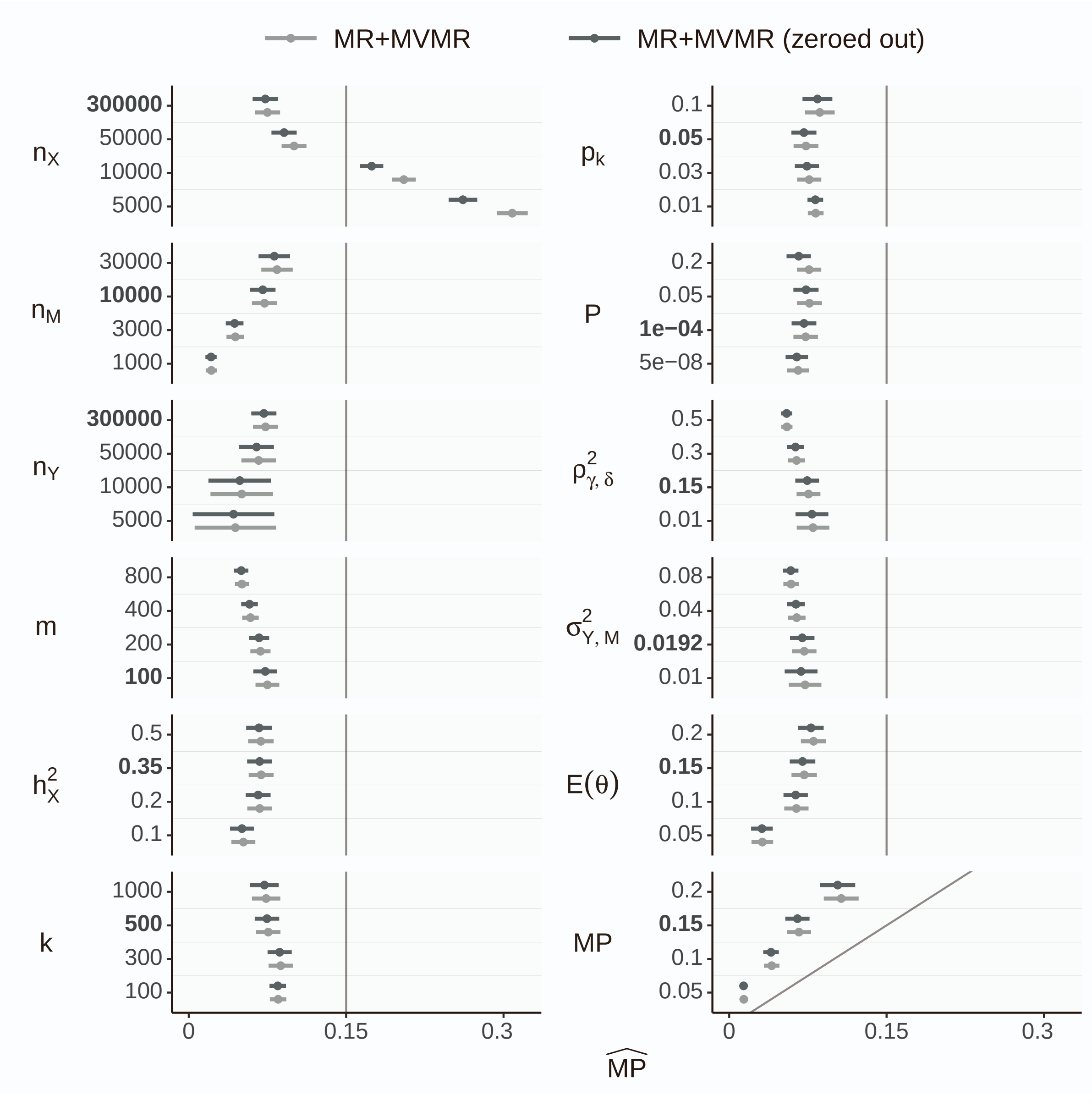

Fig. S3 | The influence of setting mediator instrument effects on the exposure to zero on MR framework performance in the simulation study. Error bars correspond to the 95% confidence interval. The true MP is displayed by the grey vertical line (diagonal in case of MP).

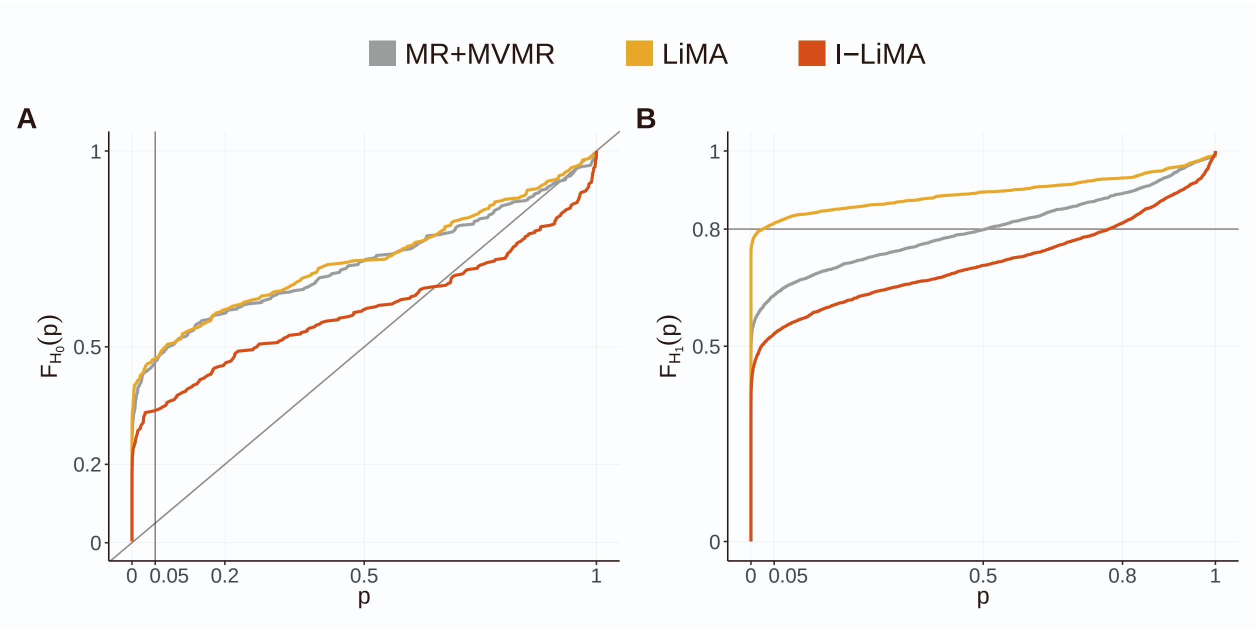

Fig. S4 | Comparison of T1E control and statistical power of methods. **A** Empirical cumulative distribution function (CDF) of P-values under the null hypothesis, $F_{H_0}(p)$, as a function of the P-value threshold p, where H_0 is based on $k = 10$ in Fig. 3e. The diagonal line represents the uniform distribution expected under proper calibration. **B** Empirical CDF of P-values under the alternative hypothesis, $F_{H_1}(p)$, where H_1 is based on the default simulation setting defined in Table S1.

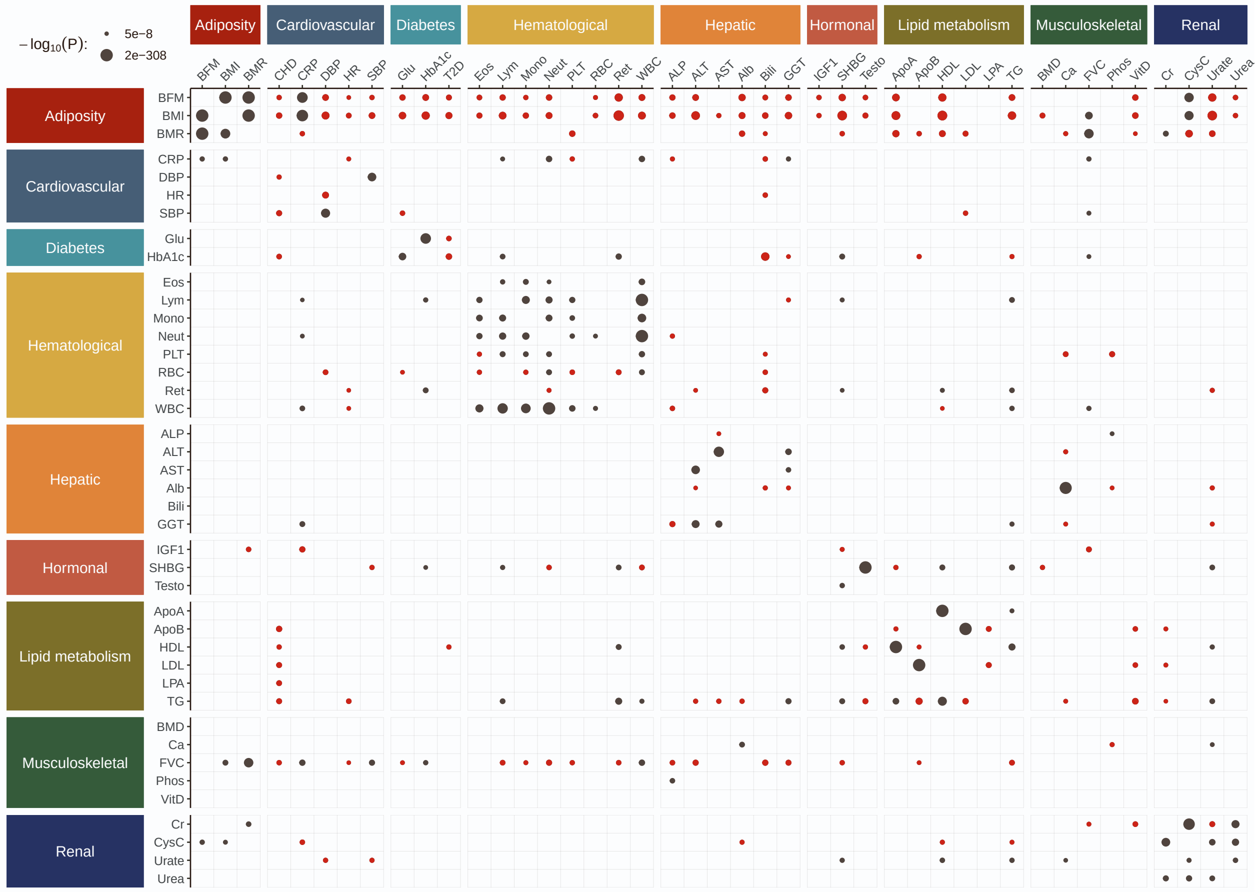

Fig. S5 | Results from Mendelian randomization analyses between UKBB complex traits used in the mediation study. Each point indicates a non-zero causal effect (MR IVW P $\leq 5 \times 10^{-8}$) from an exposure in a row to an outcome in a column, the size of which is relative to $-\log_{10}(\mathrm{P})$. Black points depict bidirectional causal effects and we are not studying those further. We are investigating mediation only if there is a causal effect from a single direction, depicted by points in red.

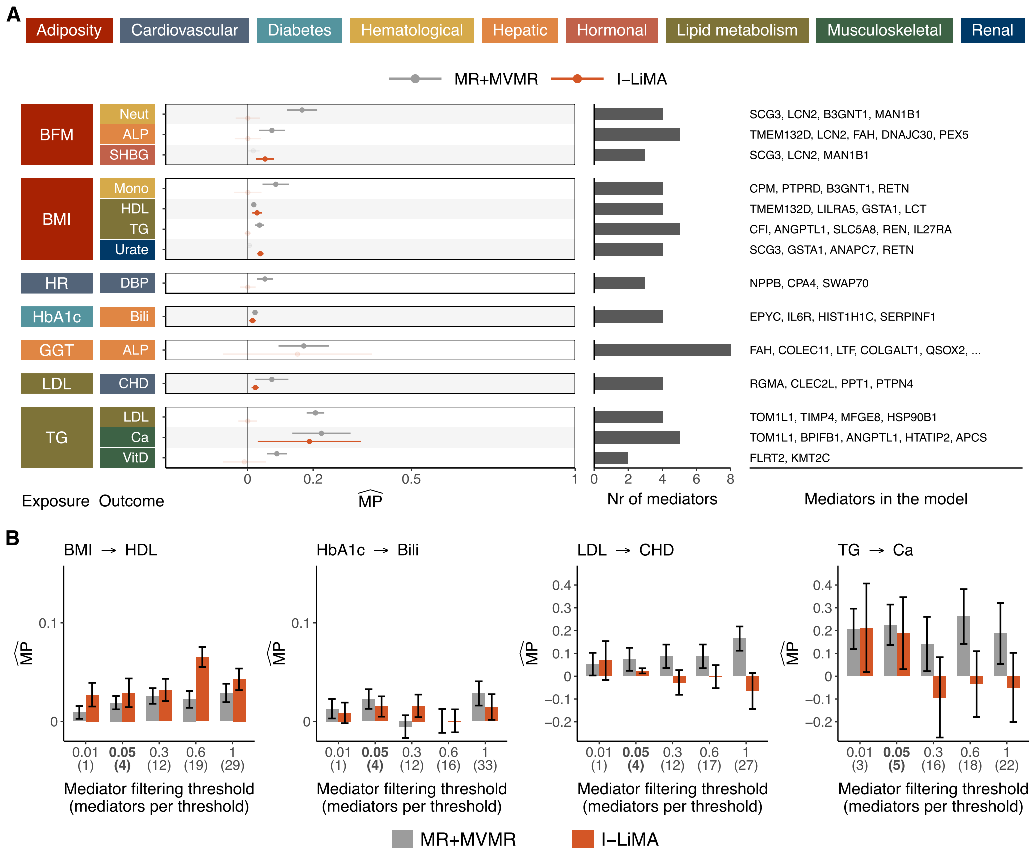

Fig. S6 | Proportion of causal effects from cardiometabolic risk factors to cardiovascular outcomes mediated by proteins from the INTERVAL 2014 study [1]. A Exposures and outcomes are colored based on their annotation (top legend). In the forest plot, line lengths correspond to 95% confidence intervals. The bars in the middle show the number of mediators selected in the models. The latter are depicted on the right, ordered based on MR IVW P-values of the causal effects from the exposure to the mediators. Results are shown, for both MR+MVMR and I-LiMA, if $\widehat{\text{MP}}$ from either method was correctly defined within $[0, 1]$ and significant after Bonferroni correction. Among those, nominally significant results are emphasized, while others are shown transparently. **B** Sensitivity analysis for the traits that had at least nominal support for non-zero protein mediation by both MR+MVMR and I-LiMA. Mediator filtering threshold 0.05 corresponds to the results in panel **A**. Mediators included at stricter thresholds are not necessarily retained at more relaxed thresholds, as filtering is followed by pruning for mediator correlation with threshold 0.1 to maintain consistency with the main analysis, introducing some additional variability.

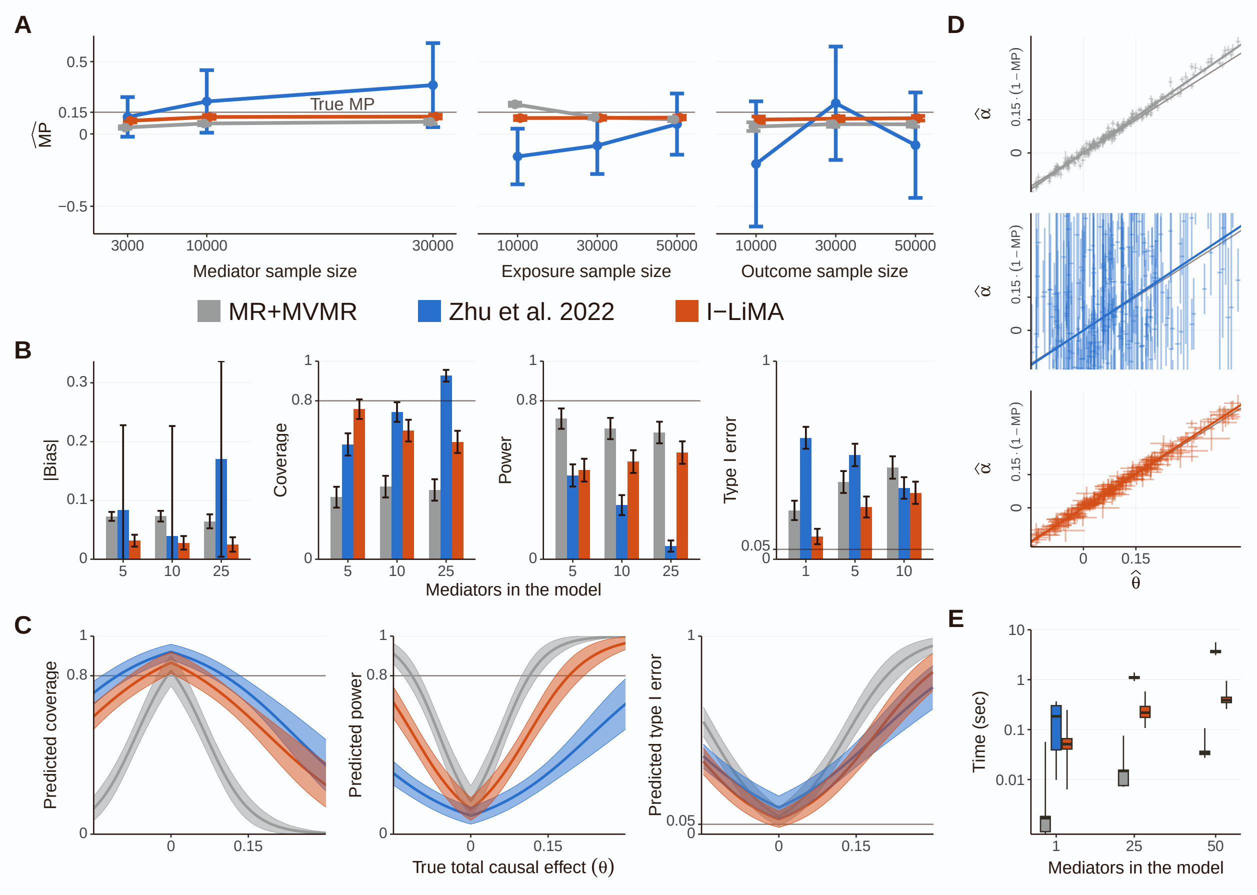

Fig. S7 | The relative performance of the method by Zhu et al. 2022 [2] in our simulations with added pleiotropy. The simulation settings, including the level of pleiotropy and CoLaus-based mediator correlation matrix Σ, are exactly like presented in the main text (Figs. 2 and 3). Error bars always correspond to 95% confidence intervals. **A** The influence of sample size of the mediators, exposure and outcome on the $\widehat{\mathrm{MP}}$ bias. **B** Model metrics in oracle simulation scenarios with variable number of mediators (all non-zero for bias, coverage and power; all null for T1E) for true $\mathrm{MP} = 0.15$. **C** Logistic regression-fitted metrics by the true total causal effect θ. **D** Direct and total causal effect estimates $(\hat{\alpha}_i, \hat{\theta}_i)$, where line lengths along each axis correspond to $2\times$ standard errors for $k = 10$. **E** Running time (in logarithmic scale) of the methods by the number of mediators in the model.

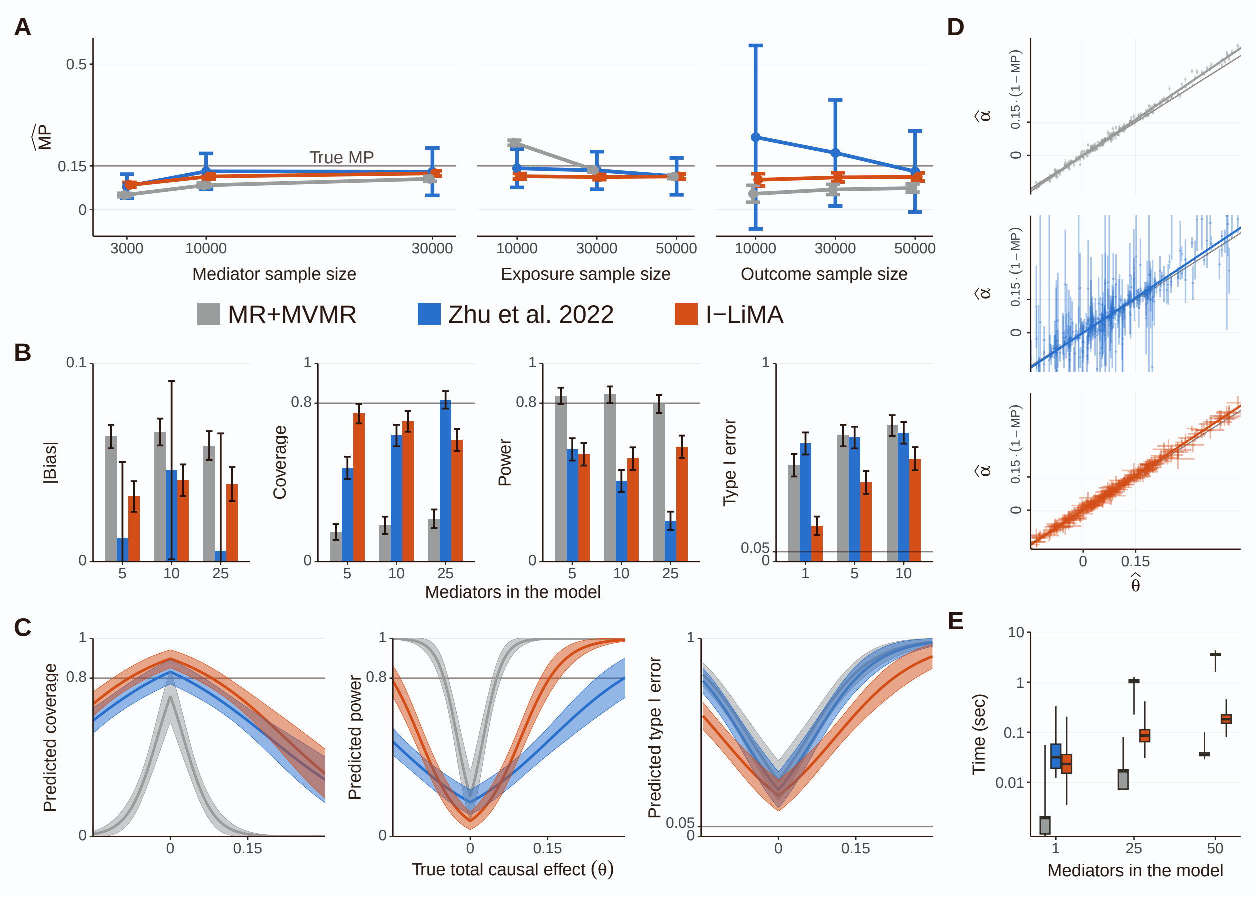

Fig. S8 | The relative performance of the method by Zhu et al. 2022 [2] in our simulations with no added pleiotropy and $\Sigma = \mathrm{I}_k$. Error bars always correspond to 95% confidence intervals. **A** The influence of sample size of the mediators, exposure and outcome on the $\widehat{\mathrm{MP}}$ bias. **B** Model metrics in oracle simulation scenarios with variable number of mediators (all non-zero for bias, coverage and power; all null for T1E) for true MP $= 0.15$. **C** Logistic regression-fitted metrics by the true total causal effect θ. **D** Direct and total causal effect estimates $(\hat{\alpha}_i, \hat{\theta}_i)$, where line lengths along each axis correspond to $2\times$ standard errors for $k = 10$. **E** Running time (in logarithmic scale) of the methods by the number of mediators in the model.

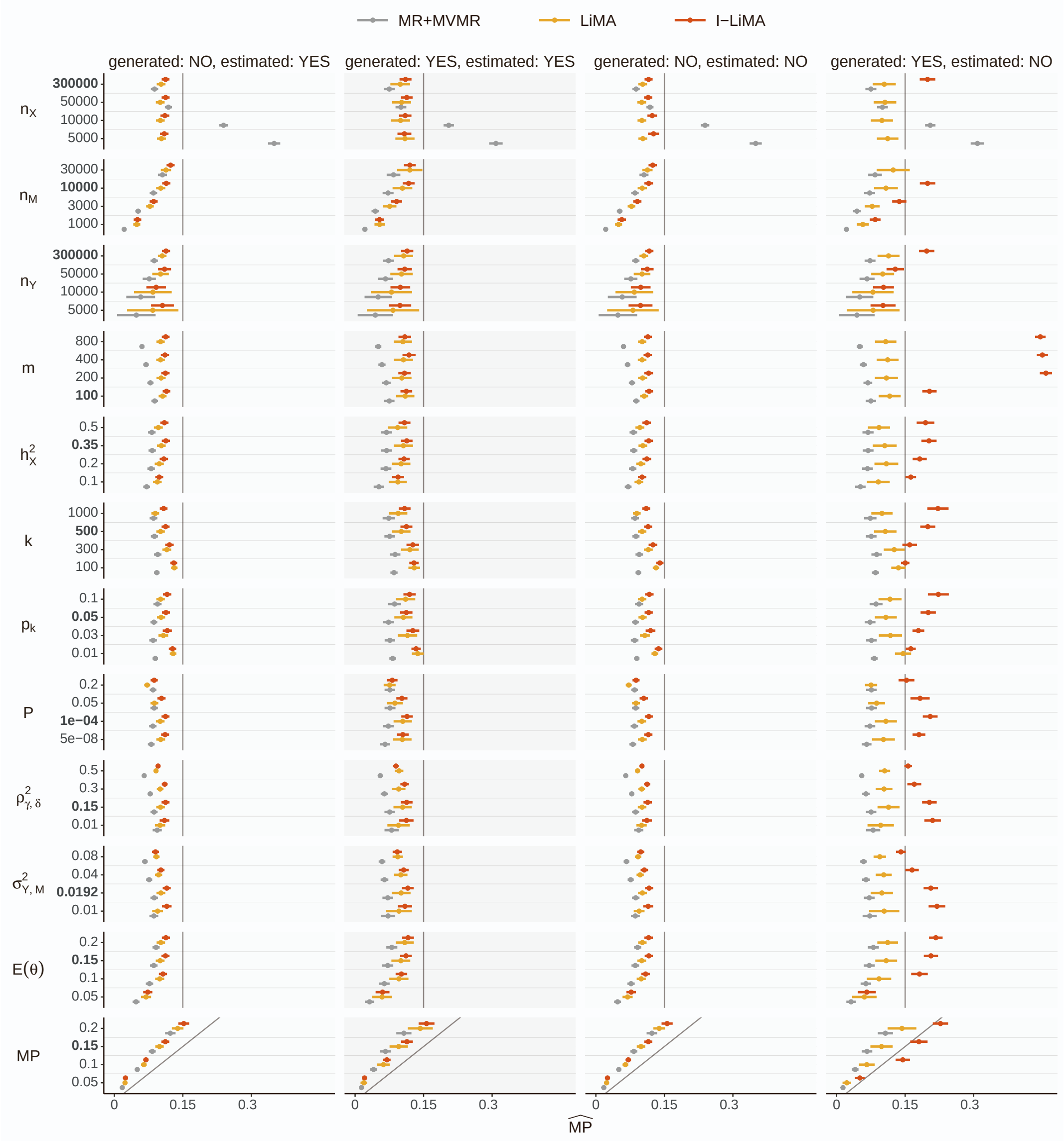

Fig. S9 | Simulation results for all combinations of parameters and pleiotropy configurations. For each parameter, the other parameters have been held fixed to the values in bold. We generated pleiotropy by fixing $\sigma_C^2 = 0.000036$, $\sigma_c^2 = 0.000012$ and $\sigma_b^2 = 0.00001$, based on estimates from the mediation analyses with UKBB exposures, Shin et al. [3] metabolite mediators and UKBB outcomes. We set these parameters to zero when generating without pleiotropy. Similarly, we fixed them to zero in estimation when we assumed no pleiotropy. In the main text, we reported the simulation results with both generated and estimated pleiotropy (shaded). Error bars correspond to the 95% confidence interval. The true MP is displayed by the gray vertical line (diagonal in case of MP).

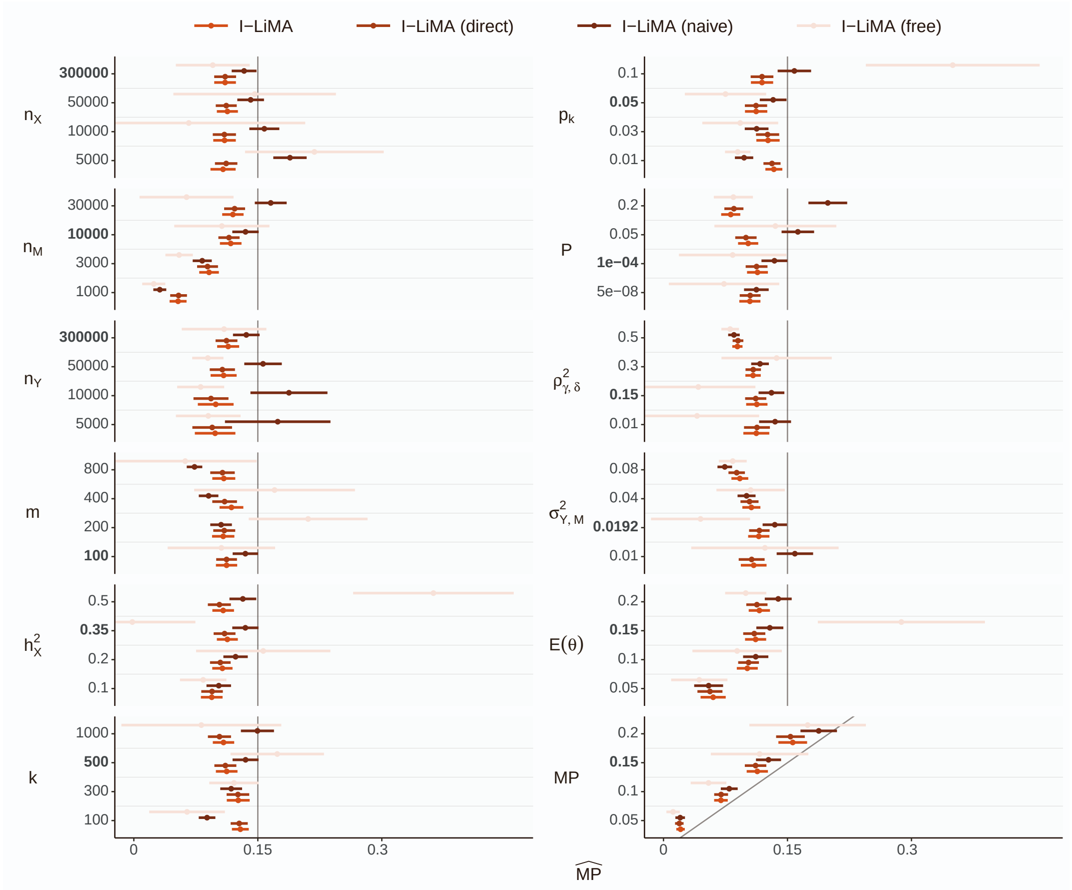

Fig. S10 | The influence of variances of mediation effects on I-LiMA performance in the simulation study. The original I-LiMA first estimates σ_γ^2 and σ_δ^2, holding them fixed before separately estimating the direct effect α and the total causal effect θ. I-LiMA (direct) fixes the variances in the same way but additionally leverages the MR framework to also fix θ before optimizing separately for α. I-LiMA (naive) utilizes the MR framework to fix $\hat{\sigma}_\gamma^2 = \mathrm{Var}(\hat{\gamma})$ and $\hat{\sigma}_\delta^2 = \mathrm{Var}(\hat{\delta})$. I-LiMA (free) does not fix the variances separately at all but rather optimizes for them together with α and θ. Error bars correspond to the 95% confidence interval. The true MP is displayed by the gray vertical line (diagonal in case of MP).

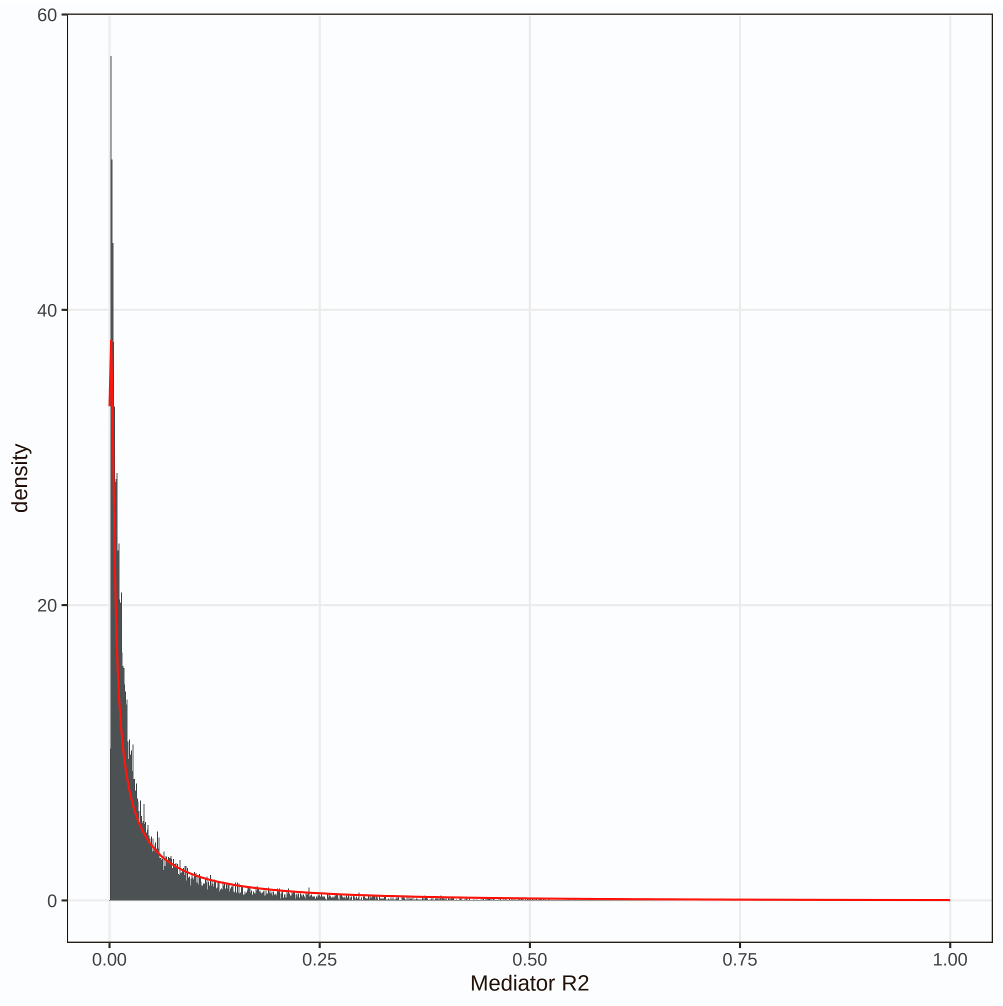

Fig. S11 | Distribution of the mediator heritabilities approximated by top eQTLs. We used the distribution of R^2 of top cis-eQTLs for each gene in the eQTLGen data [4], approximated by a Weibull distribution with shape 0.5 and scale 0.05.

Supplemental Tables

Table S1 | Default values of parameters used in the simulation study. When investigating the influence of one parameter on the bias in $\widehat{\mathrm{MP}}$, it was allowed to take values freely over its domain while the other parameters were fixed to their default values.

Parameter description	Symbol	Default value
Exposure sample size	n_X	300 000
Mediator sample size	n_M	10 000
Outcome sample size	n_Y	300 000
Exposure polygenicity (number of exposure instruments)	m	100
Exposure heritability	h^2_X	0.35
Number of mediators	k	500
Proportion of non-zero mediators	p_k	0.05
P-value threshold for selecting mediators in the model	P	$0.05/k$
Correlation of mediation effects	$\rho_{\gamma,\delta}$	0.15
Outcome variance explained by mediator instruments	$\sigma^2_{Y,M}$	0.0192
Expected total causal effect	$\mathrm{E}(\theta)$	0.15
Mediated proportion of the total causal effect	MP	0.15
Assumed mediator correlation matrix	Σ	CoLaus-based
Generated pleiotropy components	σ^2_C<br>σ^2_c<br>σ^2_b	0.000036<br>0.000012<br>0.00001
Models are optimized by assuming the presence of pleiotropy		True

Table S2 | Bias, variance, coverage and power in estimating MP by different mediation analysis methods in the simulation study. For each simulation parameter, the other parameters have been held fixed to the values in bold. The "default" row encapsulates the simulation results where each parameter has been set to their default value. Increasingly positive values are represented in darker shades of red, negative values in blue.

		Bias			Variance			Coverage			Power		
		MR fmw	LiMA	I-LiMA	MR fmw	LiMA	I-LiMA	MR fmw	LiMA	I-LiMA	MR fmw	LiMA	I-LiMA
	**default**	**−51.6%**	**−31.3%**	**−24.5%**	**0.03**	**0.09**	**0.03**	**38.2%**	**17.8%**	**63.2%**	**63.1%**	**81.4%**	**53.1%**
n_X	**300000**	−50%	−34.1%	−26.4%	0.03	0.09	0.03	37.3%	19.3%	63.9%	63.3%	82%	53%
	50000	−33.1%	−32.1%	−24.5%	0.03	0.08	0.03	38.3%	15.3%	64.9%	65.3%	82.7%	55.4%
	10000	36.7%	−33.6%	−26.8%	0.02	0.09	0.03	42.3%	17.3%	63.2%	72.3%	81.3%	52.8%
	5000	105.5%	−27.2%	−28%	0.02	0.08	0.03	33%	21%	60%	78%	83.3%	51.9%
n_M	30000	−43.9%	−20.2%	−20.1%	0.04	0.1	0.03	42.7%	16%	64.7%	59%	82%	52.2%
	**10000**	−51.8%	−30.9%	−21.9%	0.03	0.1	0.03	38.3%	20%	65.6%	62.7%	80%	54%
	3000	−70.4%	−49.5%	−39.3%	0.01	0.03	0.02	30%	21%	59.6%	67.3%	79.3%	48.3%
	1000	−85.7%	−64.1%	−64.3%	0.006	0.03	0.02	23%	20.7%	49.6%	63.3%	78.3%	40.4%
n_Y	**300000**	−51.2%	−29.2%	−23.9%	0.03	0.1	0.03	38%	17.3%	62.2%	63.3%	83.7%	52.2%
	50000	−55.6%	−32.3%	−27.6%	0.05	0.1	0.04	48.3%	26.3%	63.2%	52%	76.7%	49.5%
	10000	−66.3%	−46.9%	−34%	0.2	0.4	0.08	69%	40%	66.7%	32%	58.7%	35.8%
	5000	−70.4%	−44.7%	−34.4%	0.3	0.8	0.1	80.3%	46%	72.1%	22.7%	50.7%	26.4%
m	800	−66.2%	−30.2%	−27.4%	0.009	0.08	0.03	30.3%	18.1%	63.3%	68.3%	78.9%	51.6%
	400	−60.8%	−29.5%	−21.3%	0.01	0.08	0.03	32.7%	19%	58.8%	64%	79.3%	54.3%
	200	−54.5%	−31.9%	−27.9%	0.02	0.08	0.03	34.3%	18.7%	64.2%	61.7%	81.7%	51.4%
	**100**	−50%	−26.8%	−25.2%	0.03	0.09	0.03	40%	16.7%	63.2%	63%	81.7%	53.2%
h^2_X	0.5	−54.2%	−37.9%	−27.9%	0.03	0.09	0.03	35.7%	13%	58.4%	64%	85.3%	51.4%
	**0.35**	−54%	−29.5%	−24.6%	0.03	0.08	0.03	37.7%	19.3%	63.6%	61.7%	80.7%	53.4%
	0.2	−54.9%	−32.8%	−28.6%	0.02	0.08	0.03	42.7%	23%	62.5%	56%	72%	49.3%
	0.1	−65.3%	−37.7%	−37.2%	0.02	0.09	0.03	49.7%	40.7%	67.1%	50.7%	65%	38.5%
k	1000	−50.8%	−37.2%	−27.7%	0.04	0.07	0.03	35%	15.7%	51%	62.3%	82.7%	56.5%
	**500**	−49.5%	−32.6%	−25.1%	0.03	0.09	0.03	37.7%	18%	64.3%	62.7%	81%	53.1%
	300	−41.6%	−20.1%	−15.8%	0.03	0.07	0.04	39.3%	22.7%	71.3%	64.7%	79.3%	43.7%
	100	−43.2%	−13.7%	−14.1%	0.01	0.05	0.02	31.3%	24.7%	79%	69.7%	76.7%	43.1%
p_k	0.1	−42.4%	−26.3%	−20.5%	0.04	0.1	0.05	36%	14.7%	48.6%	63%	87%	59.1%
	**0.05**	−51.2%	−29.5%	−25.3%	0.03	0.08	0.03	35.7%	16.7%	62.3%	60.7%	82.3%	53.8%
	0.03	−49.2%	−23.5%	−15.7%	0.03	0.08	0.04	37%	25%	65.5%	61.3%	76.7%	44.4%
	0.01	−45%	−8.6%	−11%	0.01	0.03	0.02	31.7%	27%	78.9%	70.7%	78%	45.3%
P	0.2	−49.2%	−49.6%	−45.8%	0.03	0.03	0.02	35%	17%	34.6%	65%	79%	65.1%
	0.05	−49%	−42%	−31.7%	0.03	0.06	0.03	37.3%	22.3%	47.5%	64.7%	80.3%	58.6%
	**1e-04**	−51.4%	−30.5%	−24.2%	0.03	0.08	0.03	36.3%	17.7%	63.1%	62%	81.3%	51.9%
	5e-08	−56.2%	−31.1%	−30.3%	0.03	0.08	0.03	35.3%	19.7%	61.8%	61.7%	81.3%	50.2%
$\rho_{Y,\delta}$	0.5	−63.3%	−35.8%	−40.4%	0.004	0.01	0.005	11%	14%	35.5%	85%	89%	75.9%
	0.3	−57.2%	−36.5%	−27.8%	0.01	0.05	0.01	26.7%	14.7%	52%	74.7%	85.3%	63.9%
	**0.15**	−49.6%	−30.7%	−24.7%	0.02	0.08	0.03	41.7%	15.7%	61.4%	66.3%	80.3%	54.3%
	0.01	−46.7%	−36.6%	−25.1%	0.05	0.1	0.04	41.3%	19.3%	68.4%	62.3%	83.3%	42.2%
$\sigma^2_{Y,M}$	0.08	−60.7%	−37.8%	−38.4%	0.01	0.03	0.02	27.7%	22.7%	63.1%	72%	77%	45.2%
	0.04	−57.1%	−33.3%	−29.1%	0.01	0.04	0.02	29.3%	18.7%	64.3%	68.7%	80.3%	50.8%
	**0.0192**	−52.3%	−32.8%	−23.1%	0.03	0.09	0.03	39.3%	16%	59.2%	60.7%	81.3%	52.7%
	0.01	−51.8%	−36%	−27.2%	0.04	0.1	0.03	46%	18.7%	57.7%	57%	84.3%	50.9%
$E(\theta)$	0.2	−46.4%	−27.5%	−22.7%	0.05	0.1	0.04	36%	13.7%	62.7%	66.3%	87.3%	54.1%
	**0.15**	−52.4%	−33.3%	−25.7%	0.03	0.09	0.03	38.3%	18.3%	62.7%	68.3%	82%	53.2%
	0.1	−57.3%	−36.1%	−32.4%	0.02	0.05	0.02	43%	26%	65.1%	56.3%	71.7%	43.7%
	0.05	−79%	−60.6%	−59.9%	0.006	0.04	0.02	59%	54.3%	75%	27.7%	37%	17.5%
MP	0.2	−46.6%	−28.7%	−21.8%	0.05	0.1	0.05	45%	14.7%	70.7%	60.3%	82.3%	48.3%
	**0.15**	−55.7%	−36.3%	−24.3%	0.03	0.08	0.03	38%	18.7%	66.8%	62.3%	81%	52.9%
	0.1	−59.5%	−38%	−30.5%	0.01	0.04	0.01	34%	19.7%	52.5%	63.3%	75.6%	50.2%
	0.05	−72.2%	−61.7%	−58.7%	0.002	0.01	0.005	36.7%	31.7%	54.6%	54.3%	63.3%	42%

Table S3 | MR IVW pairwise causal effect estimates. A-E have varying types of exposure and outcome data.

A | UKBB exposures, UKBB outcomes.

Sheet UKBB_to_UKBB in Table S3.xlsx

B | UKBB exposures, Lotta et al. 2021 outcomes.

Sheet UKBB_to_Lotta in Table S3.xlsx

C | UKBB exposures, INTERVAL outcomes.

Sheet UKBB_to_INTERVAL in Table S3.xlsx

D | Lotta exposures, UKBB outcomes.

Sheet Lotta_to_UKBB in Table S3.xlsx

E | INTERVAL exposures, UKBB outcomes.

Sheet INTERVAL_to_UKBB in Table S3.xlsx

Table S4 | Estimates of the proportion of causal effect from risk factors to cardiovascular outcomes mediated by metabolites and proteins. A-D compare different methods, mediator filtering strategies and mediator types.

A | Lotta et al. 2021 mediators, default.

Sheet Lotta_default in Table S4.xlsx

B | Lotta et al. 2021 mediators with strict P-value based filtering.

Sheet Lotta_P_strict in Table S4.xlsx

C | Lotta et al. 2021 mediators with relaxed LD based filtering

Sheet Lotta_LD_relaxed in Table S4.xlsx

D | INTERVAL protein mediators, default

Sheet INTERVAL_default in Table S4.xlsx

Table S5 | Bias, variance, coverage and power in estimating the total causal effect θ by different mediation analysis methods in the simulation study. For each simulation parameter, the other parameters have been held fixed to the values in bold. The "default" row encapsulates the simulation results where each parameter has been set to their default value. Increasingly positive values are represented in darker shades of red, negative values in blue.

		Bias			Variance			Coverage			Power		
		MR fmw	LiMA	I-LiMA	MR fmw	LiMA	I-LiMA	MR fmw	LiMA	I-LiMA	MR fmw	LiMA	I-LiMA
	**default**	**1.2%**	**1.2%**	**1.2%**	**0.00004**	**0.00004**	**0.00005**	**95.3%**	**99.7%**	**99.6%**	**92.7%**	**89.7%**	**78.6%**
n_X	**300000**	1.2%	1.2%	1.3%	0.00004	0.00004	0.00004	95.3%	99.7%	99.7%	92.7%	89.7%	78%
	50000	0.8%	0.8%	0.7%	0.00004	0.00004	0.00004	95.3%	99.7%	100%	92.7%	90.3%	78%
	10000	−1.2%	−1.2%	−1.2%	0.00007	0.00007	0.00007	91.7%	97.7%	99.3%	93%	90%	78.1%
	5000	−3.9%	−3.9%	−3.7%	0.0001	0.0001	0.0001	86.3%	93.3%	99.3%	93%	90%	77.4%
n_M	30000	1.2%	1.2%	1.3%	0.00004	0.00004	0.00004	95.3%	99.7%	99.7%	92.7%	89.7%	77.2%
	**10000**	1.2%	1.2%	1.2%	0.00004	0.00004	0.00004	95.3%	99.7%	99.7%	92.7%	89.7%	77.7%
	3000	1.2%	1.2%	1.2%	0.00004	0.00004	0.00004	95.3%	99.7%	99.3%	92.7%	89.7%	81.2%
	1000	1.1%	1.1%	1.4%	0.00004	0.00004	0.00005	95.3%	99.7%	98.6%	92.7%	90%	83.9%
n_Y	**300000**	1.2%	1.2%	1.1%	0.00004	0.00004	0.00004	95.3%	99.7%	99.7%	92.7%	89.7%	77.7%
	50000	2.7%	2.7%	2.8%	0.00009	0.00009	0.00009	95.7%	99.7%	99.7%	90%	86%	75.9%
	10000	7.9%	7.9%	7.8%	0.0003	0.0003	0.0003	96%	99.7%	100%	81.7%	73%	70.2%
	5000	0.8%	0.8%	−0%	0.0006	0.0006	0.0006	95.7%	99.7%	99.3%	75.7%	64.7%	64.3%
m	800	1.1%	1.1%	0.6%	0.00004	0.00004	0.0002	96%	100%	99.3%	93.7%	88.6%	78.2%
	400	1%	1%	0.9%	0.00004	0.00004	0.00004	97%	98.7%	100%	93%	90.7%	79%
	200	0.7%	0.7%	0.5%	0.00004	0.00004	0.00004	93.7%	99.7%	99.7%	93.3%	89.7%	77.4%
	**100**	1.2%	1.2%	1.2%	0.00004	0.00004	0.00004	95.3%	99.7%	99.7%	92.7%	89.7%	79.3%
h^2_X	0.5	0.9%	0.9%	0.9%	0.00003	0.00003	0.00003	95.3%	99.7%	99.7%	93.7%	91%	78.4%
	**0.35**	1.2%	1.2%	1%	0.00004	0.00004	0.0001	95.3%	99.7%	99.3%	92.7%	89.7%	79.6%
	0.2	2.2%	2.2%	2.1%	0.00007	0.00007	0.00007	95.3%	99.7%	99.7%	90.3%	87.7%	77.4%
	0.1	4.1%	4.1%	4.1%	0.0001	0.0001	0.0002	96%	99.7%	99%	86.7%	83.3%	76.2%
k	1000	0.1%	0.1%	0.1%	0.00005	0.00005	0.00005	95.3%	99.7%	100%	94.3%	92.7%	82.3%
	**500**	1.2%	1.2%	1.2%	0.00004	0.00004	0.00005	95.3%	99.7%	99.3%	92.7%	89.7%	78.9%
	300	1.3%	1.3%	1.4%	0.00004	0.00004	0.00004	95.3%	100%	100%	91%	86.7%	73.4%
	100	0.8%	0.8%	0.8%	0.00004	0.00004	0.00004	92.7%	99.3%	99%	94.3%	91%	79%
p_k	0.1	1.3%	1.3%	1.4%	0.00004	0.00004	0.00004	96%	99.7%	99.7%	94.7%	91.7%	84.8%
	**0.05**	1.2%	1.2%	1.1%	0.00004	0.00004	0.00004	95.3%	99.7%	99.7%	92.7%	89.7%	79.1%
	0.03	0.5%	0.5%	0.3%	0.00004	0.00004	0.00004	95%	99.7%	99.7%	90.7%	86.7%	73%
	0.01	0.5%	0.5%	0.5%	0.00004	0.00004	0.00005	94.3%	99.3%	98.9%	93%	90%	76.1%
P	0.2	1.2%	1.2%	1.2%	0.00004	0.00004	0.00004	95.3%	99.7%	99.7%	92.7%	90%	88.1%
	0.05	1.2%	1.2%	1.1%	0.00004	0.00004	0.00008	95.3%	99.7%	99.7%	92.7%	89.7%	83.4%
	**1e-04**	1.2%	1.2%	1.2%	0.00004	0.00004	0.00004	95.3%	99.7%	99.7%	92.7%	89.7%	78.5%
	5e-08	1.2%	1.2%	1.1%	0.00004	0.00004	0.00004	95.3%	99.7%	99.7%	92.7%	89.7%	78.2%
$\rho_{Y,\delta}$	0.5	0.4%	0.4%	0.2%	0.00004	0.00004	0.00005	95.7%	99.7%	99%	98.7%	98.3%	96.9%
	0.3	1%	1%	1%	0.00004	0.00004	0.00004	95.3%	99.7%	100%	95%	93%	89.1%
	**0.15**	1.2%	1.2%	1.2%	0.00004	0.00004	0.00004	95.3%	99.7%	99.7%	92.7%	90%	77.8%
	0.01	0.3%	0.3%	0.3%	0.00004	0.00004	0.00004	95%	99.7%	100%	92.7%	90.3%	69.7%
$\sigma^2_{Y,M}$	0.08	1.4%	1.4%	1.5%	0.00004	0.00004	0.00005	95.3%	99.7%	100%	93%	90.7%	79.7%
	0.04	1.1%	1.1%	1.1%	0.00004	0.00004	0.00004	95.3%	99.7%	100%	92.7%	90%	79.1%
	**0.0192**	1.2%	1.2%	1%	0.00004	0.00004	0.0001	95.3%	99.7%	99.7%	92.7%	89.7%	79.3%
	0.01	1.2%	1.2%	1.2%	0.00004	0.00004	0.00004	95.3%	99.7%	99.7%	93%	90.3%	78.7%
$E(\theta)$	0.2	1.2%	1.2%	1.3%	0.00004	0.00004	0.00004	95.7%	99.7%	99.7%	94.3%	92.3%	79.1%
	**0.15**	1.2%	1.2%	1.1%	0.00004	0.00004	0.00004	95.3%	99.7%	100%	92.7%	89.7%	78.3%
	0.1	1.8%	1.8%	1.7%	0.00004	0.00004	0.00004	95.7%	99.7%	99.7%	88.7%	85.3%	74.9%
	0.05	3.7%	3.7%	3.7%	0.00005	0.00005	0.00005	95%	99.7%	99.3%	79.3%	71.7%	65.7%
MP	0.2	1.2%	1.2%	1.2%	0.00004	0.00004	0.00004	95.3%	99.7%	99.7%	92.7%	89.7%	72.4%
	**0.15**	1.2%	1.2%	1.2%	0.00004	0.00004	0.00004	95.3%	99.7%	99.7%	92.7%	89.7%	79.3%
	0.1	1.5%	1.5%	1.5%	0.00004	0.00004	0.00004	95.3%	99.7%	99.3%	92.3%	89.6%	83.8%
	0.05	1.5%	1.5%	1.8%	0.00004	0.00004	0.00004	95%	99.7%	98.9%	93.3%	90.3%	88.8%

Table S6 | Bias, variance, coverage and power in estimating the direct effect α by different mediation analysis methods in the simulation study. For each simulation parameter, the other parameters have been held fixed to the values in bold. The "default" row encapsulates the simulation results where each parameter has been set to their default value. Increasingly positive values are represented in darker shades of red, negative values in blue.

		Bias			Variance			Coverage			Power		
		MR fmw	LiMA	I-LiMA	MR fmw	LiMA	I-LiMA	MR fmw	LiMA	I-LiMA	MR fmw	LiMA	I-LiMA
	**default**	**9.7%**	**4.7%**	**3.3%**	**0.0005**	**0.0009**	**0.0004**	**87.4%**	**36.3%**	**91.5%**	**83.7%**	**92.8%**	**79.6%**
n_X	**300000**	10.1%	5%	3.6%	0.0005	0.0009	0.0004	86.3%	35.3%	90.9%	82%	94.3%	78.4%
	50000	5.5%	2.8%	2.4%	0.0004	0.0009	0.0004	89.7%	35%	90.9%	82.3%	92%	79.1%
	10000	−6.8%	1.2%	0.6%	0.0005	0.0008	0.0004	89.7%	34.7%	91.7%	82%	94.3%	80.6%
	5000	−20.7%	−2.8%	−1.9%	0.002	0.0008	0.0004	72.3%	37.3%	92.2%	78.3%	92.3%	78.9%
n_M	30000	8.3%	2.2%	2.8%	0.0006	0.001	0.0004	91.3%	28.7%	93.8%	78.3%	92%	77.9%
	**10000**	9.8%	5.4%	3.6%	0.0005	0.001	0.0004	87.7%	39.3%	91.4%	83.3%	93.7%	79.7%
	3000	14.1%	9.9%	8.4%	0.0005	0.0006	0.0004	73%	48.7%	86.6%	90.3%	92.7%	82.2%
	1000	16.5%	13%	13.4%	0.0006	0.0006	0.0005	55.7%	45%	77.9%	91.3%	93%	85.7%
n_Y	**300000**	9.4%	3.4%	2.9%	0.0005	0.0009	0.0004	88.3%	36%	92.1%	84%	92.7%	79.7%
	50000	10.8%	4.6%	5.5%	0.0008	0.001	0.0006	90.3%	39.3%	86.6%	74.3%	90%	75.6%
	10000	19.7%	10.3%	14%	0.002	0.004	0.001	92.3%	41.3%	83.9%	56.7%	80.3%	71.9%
	5000	21.3%	−6.4%	13.7%	0.004	0.007	0.002	95%	47.7%	87.5%	46.3%	73.3%	65.7%
m	800	12.5%	4.9%	3.8%	0.0004	0.0008	0.0006	71%	39.8%	90.3%	87.3%	93.3%	79.6%
	400	11.7%	5.9%	3.4%	0.0004	0.0009	0.0004	75.3%	36.7%	92.4%	89.3%	93%	78.4%
	200	10.3%	6.6%	4.4%	0.0004	0.0009	0.0004	85.3%	37.3%	92.2%	84%	91.3%	80.1%
	**100**	9.9%	3.1%	3.5%	0.0005	0.0008	0.0004	87.7%	37.7%	91.6%	82.3%	91%	80.3%
h^2_X	0.5	9.6%	6.3%	3.5%	0.0005	0.0009	0.0004	87.7%	30%	91.9%	83%	94%	79.7%
	**0.35**	9.7%	4.1%	2.9%	0.0005	0.0009	0.0004	86.7%	36.3%	91.5%	84.3%	92%	79.6%
	0.2	12%	6.2%	5.4%	0.0006	0.0009	0.0005	86%	46.7%	91.9%	83%	89.7%	77.4%
	0.1	16%	9.9%	9.9%	0.0007	0.001	0.0006	85.3%	61%	92%	79.7%	86%	74.5%
k	1000	9.7%	7.9%	7%	0.0005	0.0008	0.0004	88.3%	32.7%	85.7%	83%	93.3%	87.1%
	**500**	9.7%	4.9%	3.8%	0.0005	0.0008	0.0004	88%	32.7%	89.8%	85%	92.7%	79.9%
	300	10.5%	5.2%	8.1%	0.0004	0.0008	0.0004	86.3%	47.7%	91.6%	80%	89%	77.3%
	100	10.3%	7.6%	6.8%	0.0003	0.0004	0.0002	79.7%	61.3%	93.4%	91.7%	91.7%	82.8%
p_k	0.1	8.6%	4.7%	3.6%	0.0005	0.0009	0.0004	90.3%	38%	84.5%	82%	94.3%	84.1%
	**0.05**	9.8%	3.9%	3.4%	0.0005	0.0008	0.0005	85.7%	37%	90.1%	84.3%	93.3%	80.1%
	0.03	10.1%	2.6%	4.7%	0.0004	0.0009	0.0004	86%	41%	90.8%	80.7%	92.7%	73.7%
	0.01	9.9%	3.2%	3.5%	0.0003	0.0004	0.0003	83.7%	59.3%	93%	89%	90.7%	78.2%
P	0.2	9.7%	10.4%	9.3%	0.0005	0.0005	0.0004	86.7%	43.7%	69.8%	82.7%	92.3%	90.2%
	0.05	10.3%	9.9%	6.1%	0.0005	0.0007	0.0004	88.3%	37%	83.7%	84%	93%	84.1%
	**1e-04**	8.9%	4.6%	2.8%	0.0005	0.0008	0.0004	89%	38.7%	92.2%	83.3%	92.7%	78.8%
	5e-08	11.3%	5.6%	5%	0.0005	0.0009	0.0004	87.7%	39.3%	91.8%	83%	91.7%	78.5%
$\rho_{Y,\delta}$	0.5	11.6%	6.8%	7.8%	0.0003	0.0003	0.0002	67.3%	60%	83.8%	98.3%	98.3%	97.6%
	0.3	11%	7.3%	5.9%	0.0004	0.0005	0.0003	81.3%	46%	89.5%	91%	93.3%	90.5%
	**0.15**	9.4%	4.8%	3.2%	0.0004	0.0008	0.0004	85.7%	36.3%	91.8%	84.7%	93%	79.9%
	0.01	8.2%	5.8%	4.7%	0.0007	0.001	0.0006	89%	28.7%	91.5%	78%	95%	72.8%
$\sigma^2_{Y,M}$	0.08	11.6%	8.2%	8.5%	0.0004	0.0004	0.0003	71%	52%	90%	90.7%	92%	83.1%
	0.04	10.9%	5.8%	5%	0.0004	0.0005	0.0003	82%	48%	91.2%	87.7%	92.7%	79.8%
	**0.0192**	9.4%	6.1%	3.7%	0.0005	0.0009	0.0004	88%	31.3%	92.2%	82%	92.3%	79.9%
	0.01	9.3%	4.7%	3.6%	0.0007	0.001	0.0006	90.3%	30%	87.6%	79%	94.3%	79%
$E(\theta)$	0.2	8.5%	3.8%	2.8%	0.0007	0.001	0.0007	88.7%	28.4%	91.4%	81.3%	95.3%	79.5%
	**0.15**	9.8%	5.7%	3.3%	0.0005	0.0009	0.0004	87.7%	39%	92.5%	84.3%	92%	78.3%
	0.1	11.1%	5.8%	6.2%	0.0003	0.0004	0.0002	86%	45.7%	91.9%	80.7%	87.3%	75.6%
	0.05	17.7%	13.5%	15.4%	0.0002	0.0002	0.0002	86.7%	72%	92.9%	76%	79%	67.9%
MP	0.2	12.1%	5.5%	4.3%	0.0008	0.001	0.0007	86.7%	26.7%	94.9%	77.3%	94.3%	69.7%
	**0.15**	10.6%	5.7%	3.2%	0.0005	0.0009	0.0004	87.7%	35.7%	91.9%	84.3%	93.3%	81%
	0.1	8.1%	5%	4.3%	0.0003	0.0004	0.0002	85.3%	53.5%	90.9%	89%	92%	86.2%
	0.05	5.4%	4.6%	5.5%	0.0001	0.0002	0.0001	88.3%	73%	91.1%	92.7%	93%	90.7%

Table S7 | Failure characteristics of likelihood function optimization in the simulation study. The columns σ_γ^2 and σ_δ^2 indicate whether the optimization procedure for estimating corresponding parameters converged or whether the parameter estimates were very close to the zero bound (applicable only for I-LiMA). The column f indicates whether optimizing over a method's final objective function for estimating the direct effect α and total effect θ converged.

		Not converged				On bound		Undefined	
		LiMA	I-LiMA			I-LiMA		LiMA	I-LiMA
		f	f	σ_γ^2	σ_δ^2	σ_γ^2	σ_δ^2	σ_{MP}^2	σ_{MP}^2
	**default**	**0%**	**1.1%**	**0%**	**0%**	**0%**	**2.6%**	**0%**	**1.4%**
n_X	**300000**	0%	1%	0%	0%	0%	2.7%	0%	0.7%
	50000	0%	0%	0%	0%	0%	2.3%	0%	1.3%
	10000	0%	3.7%	0%	0%	0%	2.7%	0%	1.3%
	5000	0%	7.7%	0%	0%	0%	3%	0%	5%
n_M	30000	0%	3.3%	0%	0%	0%	1.7%	0%	3.3%
	**10000**	0%	1.3%	0%	0%	0%	1.7%	0%	2.3%
	3000	0%	1%	0%	0%	0%	5.7%	0%	2.7%
	1000	0%	2.7%	0%	0%	0%	23.1%	0%	4%
n_Y	**300000**	0%	1.7%	0%	0%	0%	2.7%	0%	1.7%
	50000	0%	1.3%	0%	0%	0%	7.3%	0%	2%
	10000	0%	3%	0%	0%	0%	20.7%	0%	2.7%
	5000	0%	4.3%	0%	0%	0%	31.7%	0%	3%
m	800	0%	1.3%	0%	0%	0%	2.7%	0.3%	3%
	400	0%	1.3%	0%	0%	0%	1.7%	0%	2.3%
	200	0%	0.7%	0%	0%	0%	3%	0%	1%
	**100**	0%	0%	0%	0%	0%	2.7%	0%	0.3%
h_X^2	0.5	0%	0%	0%	0%	0%	1.7%	0%	1.3%
	**0.35**	0%	1%	0%	0%	0%	2.3%	0%	1.7%
	0.2	0%	0.3%	0%	0%	0%	2.7%	0%	1%
	0.1	0%	3%	0%	0.3%	0%	8%	0%	2.3%
k	1000	0%	0.7%	0%	0%	0%	2.3%	0%	1.7%
	**500**	0%	1%	0%	0%	0%	3.7%	0%	1.7%
	300	0%	2.3%	0%	0%	0%	5%	0%	3.7%
	100	0%	2%	0%	0%	0%	8.3%	0%	2%
p_k	0.1	0%	0%	0%	0%	0%	3.7%	0%	1.3%
	**0.05**	0%	1.3%	0%	0%	0%	3%	0%	2.3%
	0.03	0%	0.7%	0%	0%	0%	4%	0%	2%
	0.01	0%	2.3%	0%	0%	0%	9.9%	0%	3%
P	0.2	0%	0.7%	0%	0%	0%	4.7%	0%	1.3%
	0.05	0%	0.7%	0%	0%	0%	1%	0%	1.3%
	**1e-04**	0%	1.7%	0%	0%	0%	2.7%	0%	1.3%
	5e-08	0%	0.7%	0%	0%	0%	3%	0%	1.7%
$\rho_{\gamma,\delta}$	0.5	0%	1.7%	0%	0%	0%	8.4%	0%	2%
	0.3	0%	1.3%	0%	0%	0%	4.7%	0%	1.3%
	**0.15**	0%	1.3%	0%	0%	0%	3%	0%	1.3%
	0.01	0%	1.3%	0%	0%	0%	2%	0%	1.7%
$\sigma_{Y,M}^2$	0.08	0%	1%	0%	0%	0%	3.7%	0%	2.7%
	0.04	0%	0.3%	0%	0%	0%	1%	0%	1%
	**0.0192**	0%	1%	0%	0%	0%	1.7%	0%	1.3%
	0.01	0%	1.3%	0%	0%	0%	4%	0%	2%
E(θ)	0.2	0%	1%	0%	0%	0%	1.7%	0.3%	2%
	**0.15**	0%	0.7%	0%	0%	0%	2.7%	0%	1.7%
	0.1	0%	1.7%	0%	0%	0%	5%	0%	1%
	0.05	0%	5%	0%	0%	0%	20.3%	0%	2.7%
MP	0.2	0%	1%	0%	0%	0%	2%	0%	1.7%
	**0.15**	0%	1%	0%	0%	0%	2.3%	0%	1%
	0.1	0%	0.7%	0%	0%	0%	4%	0.3%	0.7%
	0.05	0%	4.7%	0%	0%	0%	21.4%	0%	5.7%

Table S8 | Medically relevant UK Biobank complex traits used in the mediation study. We tested for a bidirectional causal effect between each pair of traits (with the exception of binary traits such as CHD and T2D—these were only used as outcomes) and performed a mediation analysis if there was a significant causal effect from a single direction.

Annotation	UKBB trait	Abbreviation	UKBB Field ID
ADIPOSITY	Whole body fat mass	BFM	23100
	Body mass index	BMI	21001
	Basal metabolism rate	BMR	23105
CARDIOVASCULAR	Chronic ischeamic heart disease	CHD	I25
	C-reactive protein	CRP	30710
	Diastolic blood pressure, automated reading	DBP	4079
	Pulse rate, automated reading	HR	102
	Systolic blood pressure, automated reading	SBP	4080
DIABETES	Glucose	Glu	30740
	Glycated haemoglobin	HbA1c	30750
	Type 2 diabetes, self reported	T2D	20002-1223
HEMATOLOGICAL	Eosinophill count	Eos	30150
	Lymphocyte count	Lym	30120
	Monocyte count	Mono	30130
	Neutrophil count	Neut	30140
	Platelet count	PLT	30080
	Red blood cell (erythrocyte) count	RBC	30010
	Reticulocyte count	Ret	30250
	White blood cell (leukocyte) count	WBC	30000
HEPATIC	Alkaline phosphatase	ALP	30610
	Alanine aminotransferase	ALT	30620
	Aspartate aminotransferase	AST	30650
	Albumin	Alb	30600
	Total bilirubin	Bili	30840
	Gamma glutamyltransferase	GGT	30730
HORMONAL	Insulin-like growth factor 1	IGF1	30770
	Sex hormone binding globulin	SHBG	30830
	Testosterone	Testo	30850
LIPID METABOLISM	Apolipoprotein A	ApoA	30630
	Apolipoprotein B	ApoB	30640
	High-density lipoprotein cholesterol	HDL	30760
	Low density lipoprotein cholesterol, direct	LDL	30780
	Lipoprotein A	LPA	30790
	Triglycerides	TG	30870
MUSCULOSKELETAL	Heel bone mineral density	BMD	3148
	Calcium	Ca	30680
	Forced vital capacity, spirometry	FVC	3062
	Phosphate	Phos	30810
	Vitamin D	VitD	30890
RENAL	Creatinine	Cr	30700
	Cystatin C	CysC	30720
	Serum urate	Urate	30880
	Urea	Urea	30670

Table S9 | Distribution of the number of mediator QTLs. We used the probability distribution of the number of significant *cis*-eQTLs in the eQTLGen data [4] after pruning genetic variants using a 500 kb window.

Nr of eQTLs	Probability	Cumulative probability
1	0.461	0.461
2	0.215	0.676
3	0.129	0.805
4	0.082	0.887
5	0.054	0.941
6	0.037	0.978
7	0.021	0.999
8	0.001	1

Supplemental Methods

Maximum likelihood method

Let X denote an exposure and Y an outcome such that X has a non-zero causal effect (α) on Y. Further, let $\boldsymbol{M} := (M_1, M_2, \ldots, M_k)$ represent k potential mediators of the $X \to Y$ relationship. Let $G_1, \ldots, G_m$ be instruments for X with effect sizes $\beta_1, \ldots, \beta_m$, respectively. Denote the union of the instruments for the mediators as $F_1, \ldots, F_l$, where the effect of F_i on M_j is $B_{i,j}$. Finally, the causal effects of X on mediator j is marked as γ_j, while the j-th mediator has an effect δ_j on Y. Note that the matrix of effects $B_{i,j}$ can be very sparse, but also the same instrument may have a direct effect on multiple mediators. To simplify the derivations, we introduce some column vector notations: $\boldsymbol{\beta} := (\beta_1, \ldots, \beta_m)$, $\boldsymbol{\gamma} := (\gamma_1, \ldots, \gamma_k)$ and $\boldsymbol{\delta} := (\delta_1, \ldots, \delta_m)$.

Let $C_{i,j}$ denote the (total) effect of G_i on M_j, c_i the effect of G_i on Y and b_i the effect of F_i on Y. The total causal effect of X on Y can thus be written as $\alpha = \sum_{i=1}^{k} \gamma_i \cdot \delta_i + \alpha_D = \boldsymbol{\gamma}' \cdot \boldsymbol{\delta} + \alpha_D$. Given the DAG structure, the following equations hold:

$$\begin{aligned} \boldsymbol{C} &= \boldsymbol{\beta} \cdot \boldsymbol{\gamma} + \boldsymbol{C}^{(P)} \\ \boldsymbol{c} &= \boldsymbol{\beta} \cdot (\alpha_D + \boldsymbol{\gamma}' \cdot \boldsymbol{\delta}) + \boldsymbol{c}^{(P)} \\ \boldsymbol{b} &= \boldsymbol{B} \cdot \boldsymbol{\delta} + \boldsymbol{b}^{(P)}, \end{aligned}$$

where $\boldsymbol{C}^{(P)}, \boldsymbol{c}^{(P)}$ and $\boldsymbol{b}^{(P)}$ represent uncorrelated pleiotropic effects—satisfying the InSIDE assumption. For simplicity we assume that they all come from Gaussian distributions with different variances: $\boldsymbol{C}^{(P)} \sim \mathcal{N}(0, \sigma_C^2 \cdot \Sigma_G), \boldsymbol{c}^{(P)} \sim \mathcal{N}(0, \sigma_c^2 \cdot \mathcal{I})$ and $\boldsymbol{b}^{(P)} \sim \mathcal{N}(0, \sigma_b^2 \cdot \mathcal{I})$. The covariance of the pleiotropic effects on the different mediators is proportional to the genetic correlation between them, hence Σ_G denotes the $k \times k$ genetic correlation between the mediators.

The available data we have to estimate α_D and $(\boldsymbol{\gamma}, \boldsymbol{\delta})$ are as follows:

$$\begin{aligned} \widehat{\boldsymbol{\beta}} &\sim \mathcal{N}(\boldsymbol{\beta}, D(\boldsymbol{\sigma_\beta^2})) \\ \widehat{\boldsymbol{C}} &\sim \mathcal{N}\left(\boldsymbol{\beta} \cdot \boldsymbol{\gamma} + \boldsymbol{C}^{(P)}, D(\boldsymbol{\sigma_C}) \cdot \Sigma \cdot D(\boldsymbol{\sigma_C})\right) \\ \widehat{\boldsymbol{B}} &\sim \mathcal{N}\left(\boldsymbol{B}, D(\boldsymbol{\sigma_B}) \cdot \Sigma \cdot D(\boldsymbol{\sigma_B})\right) \\ \widehat{\boldsymbol{c}} &\sim \mathcal{N}\left(\boldsymbol{\beta} \cdot (\alpha_D + \boldsymbol{\gamma}' \cdot \boldsymbol{\delta}) + \boldsymbol{c}^{(P)}, D(\boldsymbol{\sigma_c^2})\right) \\ \widehat{\boldsymbol{b}} &\sim \mathcal{N}\left(\boldsymbol{B} \cdot \boldsymbol{\delta} + \boldsymbol{b}^{(P)}, D(\boldsymbol{\sigma_b^2})\right), \end{aligned}$$

where Σ is the phenotypic correlation matrix between the mediators or in case the mediator effects are estimated in partially overlapping samples, $\Sigma_{i,j}$ is the cross-trait LD score intercept for mediator i and j. The effect size variances ($\boldsymbol{\sigma_\beta^2}, \boldsymbol{\sigma_C^2}, \boldsymbol{\sigma_B^2}, \boldsymbol{\sigma_c^2}$ and $\boldsymbol{\sigma_b^2}$) are assumed to be known (provided as part of the genome-wide summary statistics). Without loss of generality, we assume that all traits $(X, Y, M_1, \ldots, M_k)$ and genotypes $G_1, \ldots, G_m, F_1, \ldots, F_l$ are normalized to have zero mean and unit variance. Under these settings, the variances of the effect size estimates simplify to the inverse of the respective sample sizes. To further simplify the derivation, we assume that the effect size for all mediators are from the same sample (which is a reasonable assumption given that often a type of omics data or other multivariable data set may play the role of mediators). Thus, the estimator

variances simplify to

$$\begin{aligned}
D(\boldsymbol{\sigma^2_\beta}) &= \frac{1}{n_X} \cdot \mathcal{I} \\
D(\boldsymbol{\sigma^2_C}) &= \frac{1}{n_M} \cdot \mathcal{I} \\
D(\boldsymbol{\sigma^2_B}) &= \frac{1}{n_M} \cdot \mathcal{I} \\
D(\boldsymbol{\sigma^2_c}) &= \frac{1}{n_Y} \cdot \mathcal{I} \\
D(\boldsymbol{\sigma^2_b}) &= \frac{1}{n_Y} \cdot \mathcal{I}.
\end{aligned}$$

Hence the data can be written as

$$\begin{aligned}
\widehat{\boldsymbol{\beta}} &\sim \mathcal{N}\left(\boldsymbol{\beta}, \frac{1}{n_X} \cdot \mathcal{I}\right) \\
\widehat{\boldsymbol{C}} &\sim \mathcal{N}\left(\boldsymbol{\beta} \cdot \boldsymbol{\gamma}' + \boldsymbol{C}^{(P)}, \frac{1}{n_M} \cdot \Sigma\right) \\
\widehat{\boldsymbol{B}} &\sim \mathcal{N}\left(\boldsymbol{B}, \frac{1}{n_M} \cdot \Sigma\right) \\
\widehat{\boldsymbol{c}} &\sim \mathcal{N}\left(\boldsymbol{\beta} \cdot (\alpha_D + \boldsymbol{\gamma}' \cdot \boldsymbol{\delta}) + \boldsymbol{c}^{(P)}, \frac{1}{n_Y} \cdot \mathcal{I}\right) \\
\widehat{\boldsymbol{b}} &\sim \mathcal{N}\left(\boldsymbol{B} \cdot \boldsymbol{\delta} + \boldsymbol{b}^{(P)}, \frac{1}{n_Y} \cdot \mathcal{I}\right).
\end{aligned}$$

The pleiotropic effects $(\boldsymbol{C}^{(P)}, \boldsymbol{c}^{(P)}, \boldsymbol{b}^{(P)})$ can then be integrated out to yield

$$\begin{aligned}
\widehat{\boldsymbol{\beta}} &\sim \mathcal{N}\left(\boldsymbol{\beta}, \frac{1}{n_X} \cdot \mathcal{I}\right) \\
\widehat{\boldsymbol{B}} &\sim \mathcal{N}\left(\boldsymbol{B}, \frac{1}{n_M} \cdot \Sigma\right) \\
\widehat{\boldsymbol{C}} &\sim \mathcal{N}\left(\boldsymbol{\beta} \cdot \boldsymbol{\gamma}', \frac{1}{n_M} \cdot \Sigma + \sigma_C^2 \cdot \Sigma_G\right) \approx \mathcal{N}\left(\boldsymbol{\beta} \cdot \boldsymbol{\gamma}, \left(\frac{1}{n_M} + \sigma_C^2\right) \cdot \Sigma\right) \\
\widehat{\boldsymbol{c}} &\sim \mathcal{N}\left(\boldsymbol{\beta} \cdot (\alpha_D + \boldsymbol{\gamma}' \cdot \boldsymbol{\delta}), \left(\frac{1}{n_Y} + \sigma_c^2\right) \cdot \mathcal{I}\right) \\
\widehat{\boldsymbol{b}} &\sim \mathcal{N}\left(\boldsymbol{B} \cdot \boldsymbol{\delta}, \left(\frac{1}{n_Y} + \sigma_b^2\right) \cdot \mathcal{I}\right),
\end{aligned}$$

where the approximation is due to the similarity between genetic and phenotypic correlation. For more concise notation we introduce $\boldsymbol{B}_{i,\cdot}$ and $\boldsymbol{C}_{i,\cdot}$ to refer to row i of the respective matrices. Parameters $\boldsymbol{B}$ and $\boldsymbol{\beta}$ are nuisance, hence we will integrate them out in a step-wise manner. We can

rewrite the above equations as

$$\begin{aligned}
\epsilon_\beta &:= \widehat{\boldsymbol{\beta}} - \boldsymbol{\beta} \sim \mathcal{N}\left(0, \frac{1}{n_X} \cdot \mathcal{I}\right) \\
\epsilon_B &:= \widehat{\boldsymbol{B}} - \boldsymbol{B} \sim \mathcal{N}\left(0, \frac{1}{n_M} \cdot \Sigma\right) \\
\epsilon_C &:= \widehat{\boldsymbol{C}} - \boldsymbol{\beta} \cdot \boldsymbol{\gamma}' \sim \mathcal{N}\left(0, \left(\frac{1}{n_M} + \sigma_C^2\right) \cdot \Sigma\right) \\
\epsilon_c &:= \widehat{\boldsymbol{c}} - \boldsymbol{\beta} \cdot (\alpha_D + \boldsymbol{\gamma}' \cdot \boldsymbol{\delta}) \sim \mathcal{N}\left(0, \left(\frac{1}{n_Y} + \sigma_c^2\right) \cdot \mathcal{I}\right) \\
\epsilon_b &:= \widehat{\boldsymbol{b}} - \boldsymbol{B} \cdot \boldsymbol{\delta} \sim \mathcal{N}\left(0, \left(\frac{1}{n_Y} + \sigma_b^2\right) \cdot \mathcal{I}\right).
\end{aligned}$$

Combining the first two equations into the final three, we have

$$\begin{aligned}
\widehat{\boldsymbol{C}}_{i,\cdot} - \widehat{\boldsymbol{\beta}}_i \cdot \boldsymbol{\gamma}' &= (\boldsymbol{\beta}_i \cdot \boldsymbol{\gamma}' + \epsilon_C^{(i)}) - (\boldsymbol{\beta} + \epsilon_\beta^{(i)}) \cdot \boldsymbol{\gamma}' = \epsilon_C^{(i)} - \epsilon_\beta^{(i)} \cdot \boldsymbol{\gamma}' \\
\widehat{\boldsymbol{c}}_i - \widehat{\boldsymbol{\beta}}_i \cdot (\alpha_D + \boldsymbol{\gamma}' \cdot \boldsymbol{\delta}) &= (\boldsymbol{\beta} \cdot (\alpha_D + \boldsymbol{\gamma}' \cdot \boldsymbol{\delta}) + \epsilon_c^{(i)}) - (\boldsymbol{\beta} + \epsilon_\beta^{(i)}) \cdot (\alpha_D + \boldsymbol{\gamma}' \cdot \boldsymbol{\delta}) = \epsilon_c^{(i)} - \epsilon_\beta^{(i)} \cdot (\alpha_D + \boldsymbol{\gamma}' \cdot \boldsymbol{\delta}) \\
\widehat{\boldsymbol{b}}_i - \widehat{\boldsymbol{B}}_{i,\cdot} \cdot \boldsymbol{\delta} &= bB_i \cdot \boldsymbol{\delta} + \epsilon_b - (\boldsymbol{B}_i + \epsilon_B) \cdot \boldsymbol{\delta} = \epsilon_b - \epsilon_B \cdot \boldsymbol{\delta}
\end{aligned}$$

Since all error terms have zero mean, the above quantities also have zero mean and their variance can be computed (under the assumption that the three estimates come from three non-overlapping samples) as

$$\begin{aligned}
S_C &:= Var(\widehat{\boldsymbol{C}}_{i,\cdot} - \widehat{\boldsymbol{\beta}}_i \cdot \boldsymbol{\gamma}') = Var(\epsilon_C^{(i)} - \epsilon_\beta^{(i)} \cdot \boldsymbol{\gamma}') = \left(\frac{1}{n_M} + \sigma_C^2\right) \cdot \Sigma + \frac{\boldsymbol{\gamma} \cdot \boldsymbol{\gamma}'}{n_X} \\
s_c^2 &:= Var(\widehat{\boldsymbol{c}}_i - \widehat{\boldsymbol{\beta}}_i \cdot (\alpha_D + \boldsymbol{\gamma}' \cdot \boldsymbol{\delta})) = Var(\epsilon_c^{(i)} - \epsilon_\beta^{(i)} \cdot (\alpha_D + \boldsymbol{\gamma}' \cdot \boldsymbol{\delta})) \\
&= \frac{1}{n_Y} + \sigma_c^2 + \frac{(\alpha_D + \boldsymbol{\gamma}' \cdot \boldsymbol{\delta})^2}{n_X} \\
s_b^2 &:= Var(\widehat{\boldsymbol{b}}_i - \widehat{\boldsymbol{B}}_{i,\cdot} \cdot \boldsymbol{\delta}) = Var(\epsilon_b - \epsilon_B \cdot \boldsymbol{\delta}) = \frac{1}{n_Y} + \sigma_b^2 + \frac{1}{n_M} \cdot \boldsymbol{\delta}' \cdot \Sigma \cdot \boldsymbol{\delta}.
\end{aligned}$$

The last two equations imply the following form of the log-likelihood functions:

$$\log\left(Pr\left(\widehat{\boldsymbol{c}}_i | \widehat{\boldsymbol{\beta}}_i, \alpha_D, \boldsymbol{\gamma}, \boldsymbol{\delta}\right)\right) \propto -\frac{1}{2}\log(2\pi) - \frac{1}{2}\log(s_c^2) - \frac{1}{2\sigma_c^2} \cdot \left(\widehat{\boldsymbol{c}}_i - \widehat{\boldsymbol{\beta}}_i \cdot (\alpha_D + \boldsymbol{\gamma}' \cdot \boldsymbol{\delta})\right)^2 \tag{1}$$

$$\log\left(Pr\left(\widehat{\boldsymbol{b}}_i | \widehat{\boldsymbol{B}}_{i,\cdot}, \boldsymbol{\delta}\right)\right) \propto -\frac{1}{2}\log(2\pi) - \frac{1}{2}\log(s_b^2) - \frac{1}{2\sigma_b^2} \cdot \left(\widehat{\boldsymbol{b}}_i - \widehat{\boldsymbol{B}}_{i,\cdot} \cdot \boldsymbol{\delta}\right)^2. \tag{2}$$

To derive the likelihood function for $\widehat{\boldsymbol{C}}_{i,\cdot} - \widehat{\boldsymbol{\beta}}_i \cdot \boldsymbol{\gamma}'$, we need to express the inverse and the determinant of the variance-covariance matrix S_C. For this we will use the Sherman-Morrison formula and the Matrix determinant lemma:

$$\begin{aligned}
(A + uv')^{-1} &= A^{-1} - (A^{-1}uv'A^{-1})/(1 + v'A^{-1}u) \\
det(A + uv') &= det(A)(1 + v'A^{-1}u).
\end{aligned}$$

Thus for the covariance matrix inverse, we get

$$
\begin{aligned}
S_C^{-1} &= \left(\left(\frac{1}{n_M}+\sigma_C^2\right)\cdot\Sigma+\frac{\boldsymbol{\gamma}\cdot\boldsymbol{\gamma}'}{n_X}\right)^{-1}\\
&= \left(n_M^{-1}+\sigma_C^2\right)^{-1}\cdot\Sigma^{-1}-\left(n_M^{-1}+\sigma_C^2\right)^{-2}\cdot\frac{n_X^{-1}\cdot(\Sigma^{-1}\boldsymbol{\gamma})\cdot(\boldsymbol{\gamma}'\cdot\Sigma^{-1})}{1+\left(n_M^{-1}+\sigma_C^2\right)^{-1}\cdot n_X^{-1}\cdot\boldsymbol{\gamma}'\cdot\Sigma^{-1}\boldsymbol{\gamma}}\\
&= \left(n_M^{-1}+\sigma_C^2\right)^{-1}\cdot\left(\Sigma^{-1}-\frac{(\Sigma^{-1}\boldsymbol{\gamma})\cdot(\boldsymbol{\gamma}'\cdot\Sigma^{-1})}{n_X\cdot(n_M^{-1}+\sigma_C^2)+\boldsymbol{\gamma}'\cdot\Sigma^{-1}\boldsymbol{\gamma}}\right).
\end{aligned}
$$

Hence

$$
\begin{aligned}
&(\widehat{\boldsymbol{C}}_{i,\cdot}-\widehat{\boldsymbol{\beta}}_i\cdot\boldsymbol{\gamma}')\cdot S_C^{-1}(\widehat{\boldsymbol{C}}_{i,\cdot}-\widehat{\boldsymbol{\beta}}_i\cdot\boldsymbol{\gamma}')' =\\
=\;& (\widehat{\boldsymbol{C}}_{i,\cdot}-\widehat{\boldsymbol{\beta}}_i\cdot\boldsymbol{\gamma}')\cdot\left(n_M^{-1}+\sigma_C^2\right)^{-1}\cdot\left(\Sigma^{-1}-\frac{(\Sigma^{-1}\boldsymbol{\gamma})\cdot(\boldsymbol{\gamma}'\cdot\Sigma^{-1})}{n_X\cdot(n_M^{-1}+\sigma_C^2)+\boldsymbol{\gamma}'\cdot\Sigma^{-1}\boldsymbol{\gamma}}\right)(\widehat{\boldsymbol{C}}_{i,\cdot}-\widehat{\boldsymbol{\beta}}_i\cdot\boldsymbol{\gamma}')'\\
=\;& \left(n_M^{-1}+\sigma_C^2\right)^{-1}\cdot\left(\widehat{\boldsymbol{C}}_{i,\cdot}\Sigma^{-1}\widehat{\boldsymbol{C}}'_{i,\cdot}-2\widehat{\boldsymbol{\beta}}_i\widehat{\boldsymbol{C}}_{i,\cdot}\Sigma^{-1}\boldsymbol{\gamma}+\widehat{\boldsymbol{\beta}}_i^2\cdot\boldsymbol{\gamma}'\Sigma^{-1}\boldsymbol{\gamma}\right)\\
-\;& \frac{\left(n_M^{-1}+\sigma_C^2\right)^{-1}}{n_X\cdot(n_M^{-1}+\sigma_C^2)+\boldsymbol{\gamma}'\cdot\Sigma^{-1}\boldsymbol{\gamma}}\cdot\left((\widehat{\boldsymbol{C}}_{i,\cdot}\Sigma^{-1}\boldsymbol{\gamma})^2-2\widehat{\boldsymbol{\beta}}_i(\widehat{\boldsymbol{C}}_{i,\cdot}\Sigma^{-1}\boldsymbol{\gamma})\cdot(\boldsymbol{\gamma}'\Sigma^{-1}\boldsymbol{\gamma})+\widehat{\boldsymbol{\beta}}_i^2\cdot(\boldsymbol{\gamma}'\Sigma^{-1}\boldsymbol{\gamma})^2\right)\\
=\;& \frac{n_X\cdot(n_M^{-1}+\sigma_C^2)+\boldsymbol{\gamma}'\cdot\Sigma^{-1}\boldsymbol{\gamma}}{n_X\cdot(n_M^{-1}+\sigma_C^2)+\boldsymbol{\gamma}'\cdot\Sigma^{-1}\boldsymbol{\gamma}}\cdot\left(n_M^{-1}+\sigma_C^2\right)^{-1}\cdot\left(\widehat{\boldsymbol{C}}_{i,\cdot}\Sigma^{-1}\widehat{\boldsymbol{C}}'_{i,\cdot}-2\widehat{\boldsymbol{\beta}}_i\widehat{\boldsymbol{C}}_{i,\cdot}\Sigma^{-1}\boldsymbol{\gamma}+\widehat{\boldsymbol{\beta}}_i^2\cdot\boldsymbol{\gamma}'\Sigma^{-1}\boldsymbol{\gamma}\right)\\
-\;& \frac{\left(n_M^{-1}+\sigma_C^2\right)^{-1}}{n_X\cdot(n_M^{-1}+\sigma_C^2)+\boldsymbol{\gamma}'\cdot\Sigma^{-1}\boldsymbol{\gamma}}\cdot\left((\widehat{\boldsymbol{C}}_{i,\cdot}\Sigma^{-1}\boldsymbol{\gamma})^2-2\widehat{\boldsymbol{\beta}}_i(\widehat{\boldsymbol{C}}_{i,\cdot}\Sigma^{-1}\boldsymbol{\gamma})\cdot(\boldsymbol{\gamma}'\Sigma^{-1}\boldsymbol{\gamma})+\widehat{\boldsymbol{\beta}}_i^2\cdot(\boldsymbol{\gamma}'\Sigma^{-1}\boldsymbol{\gamma})^2\right)\\
=\;& \frac{\boldsymbol{\gamma}'\cdot\Sigma^{-1}\boldsymbol{\gamma}}{n_X\cdot(n_M^{-1}+\sigma_C^2)+\boldsymbol{\gamma}'\cdot\Sigma^{-1}\boldsymbol{\gamma}}\cdot\left(n_M^{-1}+\sigma_C^2\right)^{-1}\cdot\left(\widehat{\boldsymbol{C}}_{i,\cdot}\Sigma^{-1}\widehat{\boldsymbol{C}}'_{i,\cdot}-2\widehat{\boldsymbol{\beta}}_i\widehat{\boldsymbol{C}}_{i,\cdot}\Sigma^{-1}\boldsymbol{\gamma}+\widehat{\boldsymbol{\beta}}_i^2\cdot\boldsymbol{\gamma}'\Sigma^{-1}\boldsymbol{\gamma}\right)\\
+\;& \frac{n_X}{n_X\cdot(n_M^{-1}+\sigma_C^2)+\boldsymbol{\gamma}'\cdot\Sigma^{-1}\boldsymbol{\gamma}}\cdot\left(\widehat{\boldsymbol{C}}_{i,\cdot}\Sigma^{-1}\widehat{\boldsymbol{C}}'_{i,\cdot}-2\widehat{\boldsymbol{\beta}}_i\widehat{\boldsymbol{C}}_{i,\cdot}\Sigma^{-1}\boldsymbol{\gamma}+\widehat{\boldsymbol{\beta}}_i^2\cdot\boldsymbol{\gamma}'\Sigma^{-1}\boldsymbol{\gamma}\right)\\
-\;& \frac{\left(n_M^{-1}+\sigma_C^2\right)^{-1}}{n_X\cdot(n_M^{-1}+\sigma_C^2)+\boldsymbol{\gamma}'\cdot\Sigma^{-1}\boldsymbol{\gamma}}\cdot\left((\widehat{\boldsymbol{C}}_{i,\cdot}\Sigma^{-1}\boldsymbol{\gamma})^2-2\widehat{\boldsymbol{\beta}}_i(\widehat{\boldsymbol{C}}_{i,\cdot}\Sigma^{-1}\boldsymbol{\gamma})\cdot(\boldsymbol{\gamma}'\Sigma^{-1}\boldsymbol{\gamma})+\widehat{\boldsymbol{\beta}}_i^2\cdot(\boldsymbol{\gamma}'\Sigma^{-1}\boldsymbol{\gamma})^2\right)\\
=\;& \frac{n_X}{n_X\cdot(n_M^{-1}+\sigma_C^2)+\boldsymbol{\gamma}'\cdot\Sigma^{-1}\boldsymbol{\gamma}}\cdot\left(\widehat{\boldsymbol{C}}_{i,\cdot}\Sigma^{-1}\widehat{\boldsymbol{C}}'_{i,\cdot}-2\widehat{\boldsymbol{\beta}}_i\widehat{\boldsymbol{C}}_{i,\cdot}\Sigma^{-1}\boldsymbol{\gamma}+\widehat{\boldsymbol{\beta}}_i^2\cdot\boldsymbol{\gamma}'\Sigma^{-1}\boldsymbol{\gamma}\right)\\
+\;& \frac{\left(n_M^{-1}+\sigma_C^2\right)^{-1}}{n_X\cdot(n_M^{-1}+\sigma_C^2)+\boldsymbol{\gamma}'\cdot\Sigma^{-1}\boldsymbol{\gamma}}\cdot\left((\widehat{\boldsymbol{C}}_{i,\cdot}\Sigma^{-1}\widehat{\boldsymbol{C}}'_{i,\cdot})\cdot(\boldsymbol{\gamma}'\cdot\Sigma^{-1}\boldsymbol{\gamma})-(\widehat{\boldsymbol{C}}_{i,\cdot}\Sigma^{-1}\boldsymbol{\gamma})^2\right).
\end{aligned}
$$

The determinant takes the form

$$
\begin{aligned}
det\left(\left(n_M^{-1}+\sigma_C^2\right)\cdot\Sigma+\frac{\boldsymbol{\gamma}\cdot\boldsymbol{\gamma}'}{n_X}\right) &= \left(n_M^{-1}+\sigma_C^2\right)^k\cdot det(\Sigma)\cdot\left(1+n_X^{-1}\cdot(n_M^{-1}+\sigma_C^2)^{-1}\cdot\boldsymbol{\gamma}'\cdot\Sigma^{-1}\boldsymbol{\gamma}\right)\\
&= n_X^{-1}\cdot\left(n_M^{-1}+\sigma_C^2\right)^{k-1}\cdot det(\Sigma)\cdot\left(n_X\cdot(n_M^{-1}+\sigma_C^2)+\boldsymbol{\gamma}'\cdot\Sigma^{-1}\boldsymbol{\gamma}\right)\\
&= \left(n_M^{-1}+\sigma_C^2\right)^{k-1}\cdot det(\Sigma)\cdot\left(n_M^{-1}+\sigma_C^2+n_X^{-1}\cdot\boldsymbol{\gamma}'\cdot\Sigma^{-1}\boldsymbol{\gamma}\right).
\end{aligned}
$$

This enables us to simplify the log-likelihood function for $\widehat{\boldsymbol{C}}_{i,\cdot}$ to

$$\begin{aligned}
\log(Pr(\widehat{\boldsymbol{C}}_{i,\cdot}|\widehat{\boldsymbol{\beta}}_i,\boldsymbol{\gamma})) &= -\frac{k}{2}log(2\pi) - \frac{1}{2}\cdot\log(|S_C|) - \frac{1}{2}(\widehat{\boldsymbol{C}}_{i,\cdot} - \widehat{\boldsymbol{\beta}}_i\cdot\boldsymbol{\gamma}')\cdot S_C^{-1}(\widehat{\boldsymbol{C}}_{i,\cdot} - \widehat{\boldsymbol{\beta}}_i\cdot\boldsymbol{\gamma}')' \\
&= -\frac{k}{2}log(2\pi) - \frac{1}{2}\log(|\Sigma|) - \frac{k-1}{2}\cdot\log(n_M^{-1}+\sigma_C^2) \\
&- \frac{1}{2}\cdot\log\left(n_M^{-1}+\sigma_C^2+n_X^{-1}\cdot\boldsymbol{\gamma}'\cdot\Sigma^{-1}\boldsymbol{\gamma}\right) \\
&- \frac{1}{2}\cdot\frac{n_X}{n_X\cdot(n_M^{-1}+\sigma_C^2)+\boldsymbol{\gamma}'\cdot\Sigma^{-1}\boldsymbol{\gamma}}\cdot\left(\widehat{\boldsymbol{C}}_{i,\cdot}\Sigma^{-1}\widehat{\boldsymbol{C}}_{i,\cdot}' - 2\widehat{\boldsymbol{\beta}}_i\widehat{\boldsymbol{C}}_{i,\cdot}\Sigma^{-1}\boldsymbol{\gamma} + \widehat{\boldsymbol{\beta}}_i^2\cdot\boldsymbol{\gamma}'\Sigma^{-1}\boldsymbol{\gamma}\right) \\
&- \frac{1}{2}\cdot\frac{\left(n_M^{-1}+\sigma_C^2\right)^{-1}}{n_X\cdot(n_M^{-1}+\sigma_C^2)+\boldsymbol{\gamma}'\cdot\Sigma^{-1}\boldsymbol{\gamma}}\cdot\left((\widehat{\boldsymbol{C}}_{i,\cdot}\Sigma^{-1}\widehat{\boldsymbol{C}}_{i,\cdot}')\cdot(\boldsymbol{\gamma}'\cdot\Sigma^{-1}\boldsymbol{\gamma}) - (\widehat{\boldsymbol{C}}_{i,\cdot}\Sigma^{-1}\boldsymbol{\gamma})^2\right).
\end{aligned} \tag{3}$$

Combining Eqs. (1-3) and denoting $\boldsymbol{\theta} := (\alpha_D, \boldsymbol{\gamma}, \boldsymbol{\delta})$, the complete data likelihood is thus of the following form:

$$\begin{aligned}
Pr(\widehat{\boldsymbol{c}},\widehat{\boldsymbol{b}},\widehat{\boldsymbol{C}},\widehat{\boldsymbol{B}},\widehat{\boldsymbol{\beta}}|\boldsymbol{\theta}) &= Pr(\widehat{\boldsymbol{c}},\widehat{\boldsymbol{b}},\widehat{\boldsymbol{C}}|\widehat{\boldsymbol{B}},\widehat{\boldsymbol{\beta}},\boldsymbol{\theta})\cdot Pr(\widehat{\boldsymbol{B}},\widehat{\boldsymbol{\beta}}|\boldsymbol{\theta}) \\
&= Pr(\widehat{\boldsymbol{c}},\widehat{\boldsymbol{b}},\widehat{\boldsymbol{C}}|\widehat{\boldsymbol{B}},\widehat{\boldsymbol{\beta}},\boldsymbol{\theta})\cdot Pr(\widehat{\boldsymbol{B}})\cdot Pr(\widehat{\boldsymbol{\beta}}) \\
&\propto Pr(\widehat{\boldsymbol{c}},\widehat{\boldsymbol{b}},\widehat{\boldsymbol{C}}|\widehat{\boldsymbol{B}},\widehat{\boldsymbol{\beta}},\boldsymbol{\theta}) \\
&= Pr(\widehat{\boldsymbol{b}}|\widehat{\boldsymbol{B}},\widehat{\boldsymbol{\beta}},\boldsymbol{\theta})\cdot Pr(\widehat{\boldsymbol{c}},\widehat{\boldsymbol{C}}|\widehat{\boldsymbol{B}},\widehat{\boldsymbol{\beta}},\boldsymbol{\theta}) \\
&= Pr\left(\widehat{\boldsymbol{b}}|\widehat{\boldsymbol{B}},\boldsymbol{\theta}\right)\cdot Pr(\widehat{\boldsymbol{c}},\widehat{\boldsymbol{C}}|\widehat{\boldsymbol{\beta}},\boldsymbol{\theta}) \\
&= Pr\left(\widehat{\boldsymbol{b}}|\widehat{\boldsymbol{B}},\boldsymbol{\theta}\right)\cdot Pr\left(\widehat{\boldsymbol{c}}|\widehat{\boldsymbol{C}},\widehat{\boldsymbol{\beta}},\boldsymbol{\theta}\right)\cdot Pr(\widehat{\boldsymbol{C}}|\widehat{\boldsymbol{\beta}},\boldsymbol{\theta}) \\
&= \underbrace{Pr\left(\widehat{\boldsymbol{b}}|\widehat{\boldsymbol{B}},\boldsymbol{\delta}\right)}_{Eq.\ 1}\cdot\underbrace{Pr\left(\widehat{\boldsymbol{c}}|\widehat{\boldsymbol{\beta}},\boldsymbol{\theta}\right)}_{Eq.\ 2}\cdot\underbrace{Pr(\widehat{\boldsymbol{C}}|\widehat{\boldsymbol{\beta}},\boldsymbol{\gamma})}_{Eq.\ 3}.
\end{aligned}$$

Finally, we have

$$\log\left(Pr(\widehat{\boldsymbol{c}},\widehat{\boldsymbol{b}},\widehat{\boldsymbol{C}},\widehat{\boldsymbol{B}},\widehat{\boldsymbol{\beta}}|\boldsymbol{\theta})\right) \propto \sum_{i=1}^{l}\log\left(Pr\left(\widehat{\boldsymbol{b}}_i|\widehat{\boldsymbol{B}}_{i,\cdot},\boldsymbol{\delta}\right)\right) + \sum_{i=1}^{m}\log\left(Pr\left(\widehat{\boldsymbol{c}}_i|\widehat{\boldsymbol{\beta}}_i,\boldsymbol{\theta}\right)\right) + \sum_{i=1}^{m}\log\left(Pr(\widehat{\boldsymbol{C}}_{i,\cdot}|\widehat{\boldsymbol{\beta}},\boldsymbol{\gamma})\right).$$

Integrated likelihood method

Previously, we derived the likelihood for observing any sample-specific values of summary statistics conditional on causal effects α_D, γ and δ:

$$\Pr(\text{sumstats} \mid \alpha_D,\gamma,\delta) = \Pr(\hat{b}\mid\hat{B},\delta)\cdot\Pr(\hat{C}\mid\hat{\beta},\gamma)\cdot\Pr(\hat{c}\mid\hat{\beta},\alpha_D,\gamma,\delta), \tag{4}$$

with the following individual components:

$$\begin{aligned}
\Pr(\hat{b}\mid\hat{B},\delta) &= \mathcal{N}\left(\hat{B}\delta,\,\Lambda_{s_b^2}\right), \\
Pr(\hat{C}\mid\hat{\beta},\gamma) &= \mathcal{MN}\left(\hat{\beta}\gamma',\,I_m,\,S_C\right) \iff \mathrm{vec}(\hat{C}) \sim \mathcal{N}\left(\mathrm{vec}(\hat{\beta}\gamma'),\,S_C\otimes I_m\right), \\
\Pr(\hat{c}\mid\hat{\beta},\alpha_D,\gamma,\delta) &= \mathcal{N}\left(\hat{\beta}\cdot(\alpha_D+\gamma'\delta),\,\Lambda_{s_c^2}\right),
\end{aligned}$$

where Λ_x denotes a diagonal matrix with x on the diagonal and I_m denotes an $m \times m$ identity matrix. The variance components were defined as follows:

$$s_b^2 = \frac{1}{n_Y} + \sigma_b^2 + \frac{1}{n_M} \cdot \delta' \cdot \Sigma \cdot \delta, \tag{5}$$

$$s_c^2 = \frac{1}{n_Y} + \sigma_c^2 + \frac{(\alpha_D + \gamma' \cdot \delta)^2}{n_X}, \tag{6}$$

$$S_C = \left(\frac{1}{n_M} + \sigma_C^2\right) \cdot \Sigma + \frac{\gamma \cdot \gamma'}{n_X}. \tag{7}$$

As the number of parameters to estimate grows linearly with the number of mediators (there is a (γ_i, δ_i) pair for each mediator M_i), this likelihood function becomes difficult to optimize when the number of mediators gets large. Note however that we are not actually interested in the precise values of the mediation effects γ and δ. As such, we will seek to integrate these effects out from the likelihood in Equation 4.

Prior distribution

Before we can proceed to integrating out the mediation effects, we need to fix a prior distribution that these effects are assumed to follow. In this case, we assume that the mediation effect pairs of each mediator follow the bivariate Gaussian distribution below:

$$\begin{pmatrix} \gamma_i \\ \delta_i \end{pmatrix} \sim \mathcal{N}\left(\begin{pmatrix} 0 \\ 0 \end{pmatrix}, \begin{pmatrix} \sigma_\gamma^2 & \sigma_{\gamma,\delta} \\ \sigma_{\gamma,\delta} & \sigma_\delta^2 \end{pmatrix} \right).$$

The average indirect effect $\mathrm{E}(\gamma_i \delta_i)$ from the exposure to the outcome through the mediator M_i is thus determined by the covariance $\sigma_{\gamma,\delta}$. We assume that the pairs (γ_i, δ_i) for different mediators $M_i, M_j : i \neq j$ are independent.

To facilitate working with the total mediation effect $\gamma'\delta$ in the expectation of $\hat{c}$, we define $\omega = \gamma'\delta$ as a random variable and consider it as an additional parameter to integrate out. The distribution for this sum of products of dependent Gaussian random variables can be described through a multiplication of the absolute value of its expectation, its linear term in the exponential and a modified Bessel function of the second kind with it as an argument [5]. However, for ease of integration, instead we approximate it by a Gaussian distribution of mean

$$\mathrm{E}(\omega) = \mathrm{E}(\gamma'\delta) = k \cdot \mathrm{E}(\gamma_i \delta_i) = k \cdot \sigma_{\gamma,\delta} \tag{8}$$

and variance

$$\sigma_\omega^2 := \mathrm{Var}(\omega) = \mathrm{Var}(\gamma'\delta) = k \cdot \mathrm{Var}(\gamma_i \delta_i) = k \cdot \left(\mathrm{E}(\gamma_i^2 \delta_i^2) - \sigma_{\gamma,\delta}^2\right) = k \cdot (\sigma_\gamma^2 \sigma_\delta^2 + \sigma_{\gamma,\delta}^2), \tag{9}$$

where we used the law of total expectation, the properties of conditional Gaussian and the fourth moment of Gaussian distribution to express

$$\begin{aligned} \mathrm{E}(\gamma_i^2 \delta_i^2) &= \mathrm{E}\left[\mathrm{E}(\gamma_i^2 \delta_i^2 \mid \gamma_i)\right] = \mathrm{E}\left[\gamma_i^2 \cdot \mathrm{E}(\delta_i^2 \mid \gamma_i)\right] = \mathrm{E}\left[\gamma_i^2 \cdot \mathrm{Var}(\delta_i \mid \gamma_i) + \gamma_i^2 \cdot \left(\mathrm{E}(\delta_i \mid \gamma_i)\right)^2\right] \\ &= \mathrm{E}\left[\gamma_i^2 \cdot \left(\sigma_\delta^2 - \frac{\sigma_{\gamma,\delta}^2}{\sigma_\gamma^2}\right) + \gamma_i^2 \cdot \left(\gamma_i \frac{\sigma_{\gamma,\delta}}{\sigma_\gamma^2}\right)^2\right] = \left(\sigma_\delta^2 - \frac{\sigma_{\gamma,\delta}^2}{\sigma_\gamma^2}\right) \cdot \mathrm{E}(\gamma_i^2) + \frac{\sigma_{\gamma,\delta}^2}{\sigma_\gamma^4} \cdot \mathrm{E}(\gamma_i^4) \\ &= \sigma_\delta^2 \sigma_\gamma^2 - \sigma_{\gamma,\delta}^2 + 3\sigma_{\gamma,\delta}^2 = \sigma_\delta^2 \sigma_\gamma^2 + 2\sigma_{\gamma,\delta}^2. \end{aligned}$$

The Gaussian prior for ω is thus

$$\omega \sim \mathcal{N}\Big(k \cdot \sigma_{\gamma,\delta}, \quad k \cdot \big(\sigma_\gamma^2 \sigma_\delta^2 + \sigma_{\gamma,\delta}^2\big)\Big),$$

which provides a good approximation for its true form.

In order to combine the priors for (γ_i, δ_i) and ω, we assume they are jointly Gaussian. Thus we need to find the covariance of ω with γ_i and δ_i for all $i = 1, 2, \dots, k$. Due to independence of mediation effects:

$$\mathrm{Cov}(\gamma_i, \omega) = \mathrm{Cov}(\gamma_i, \gamma_i\delta_i) = \mathrm{E}\Big[\big(\gamma_i - \mathrm{E}(\gamma_i)\big)\big(\gamma_i\delta_i - \mathrm{E}(\gamma_i\delta_i)\big)\Big] = \mathrm{E}\big(\gamma_i^2\delta_i - \gamma_i\sigma_{\gamma,\delta}\big) = \mathrm{E}(\gamma_i^2\delta_i),$$
$$\mathrm{Cov}(\delta_i, \omega) = \mathrm{Cov}(\delta_i, \gamma_i\delta_i) = \mathrm{E}\Big[\big(\delta_i - \mathrm{E}(\delta_i)\big)\big(\gamma_i\delta_i - \mathrm{E}(\gamma_i\delta_i)\big)\Big] = \mathrm{E}\big(\gamma_i\delta_i^2 - \delta_i\sigma_{\gamma,\delta}\big) = \mathrm{E}(\gamma_i\delta_i^2).$$

Using the law of total expectation, we have

$$\mathrm{E}(\gamma_i^2\delta_i) = \mathrm{E}\big[\mathrm{E}(\gamma_i^2\delta_i \mid \gamma_i)\big] = \mathrm{E}\big[\gamma_i^2 \cdot \mathrm{E}(\delta_i \mid \gamma_i)\big] = \mathrm{E}\left[\gamma_i^2 \cdot \left(\mathrm{E}(\delta_i) + \frac{\sigma_{\gamma,\delta}}{\sigma_\gamma^2}\big(\gamma_i - \mathrm{E}(\gamma_i)\big)\right)\right] = \frac{\sigma_{\gamma,\delta}}{\sigma_\gamma^2}\mathrm{E}(\gamma_i^3),$$
$$\mathrm{E}(\gamma_i\delta_i^2) = \mathrm{E}\big[\mathrm{E}(\gamma_i\delta_i^2 \mid \delta_i)\big] = \mathrm{E}\big[\delta_i^2 \cdot \mathrm{E}(\gamma_i \mid \delta_i)\big] = \mathrm{E}\left[\delta_i^2 \cdot \left(\mathrm{E}(\gamma_i) + \frac{\sigma_{\gamma,\delta}}{\sigma_\delta^2}\big(\delta_i - \mathrm{E}(\delta_i)\big)\right)\right] = \frac{\sigma_{\gamma,\delta}}{\sigma_\delta^2}\mathrm{E}(\delta_i^3),$$

where we have once again utilised the properties of conditional Gaussian distributions. Since Gaussian distributions have skewness zero and both γ_i and δ_i are marginally Gaussian with expectation 0, then the third moments are $\mathrm{E}(\gamma_i^3) = 0$ and $\mathrm{E}(\delta_i^3) = 0$. The covariances are thus

$$\mathrm{Cov}(\gamma_i, \omega) = \frac{\sigma_{\gamma,\delta}}{\sigma_\gamma^2}\mathrm{E}(\gamma_i^3) = 0,$$
$$\mathrm{Cov}(\delta_i, \omega) = \frac{\sigma_{\gamma,\delta}}{\sigma_\delta^2}\mathrm{E}(\delta_i^3) = 0.$$

Finally, we can construct the joint prior for γ, δ and w as a $(2k+1)$-variate Gaussian distribution:

$$\begin{pmatrix}\gamma\\ \delta\\ \omega\end{pmatrix} \sim \mathcal{N}\left(\begin{pmatrix}0\\0\\0\end{pmatrix}, \begin{pmatrix}\sigma_\gamma^2 I_k & \sigma_{\gamma,\delta} I_k & 0\\ \sigma_{\gamma,\delta} I_k & \sigma_\delta^2 I_k & 0\\ 0 & 0 & \sigma_\omega^2\end{pmatrix}\right),$$

where I_k refers to the $k \times k$ identity matrix.

Marginal distribution

To integrate out mediation effects γ, δ and ω, we need to find the marginal distribution

$$\Pr(\text{sumstats} \mid \alpha_D) = \int_{(\gamma,\delta,\omega)} \Big[\Pr(\text{sumstats} \mid \alpha_D, \gamma, \delta, \omega) \cdot \Pr(\gamma, \delta, \omega)\Big] d(\gamma, \delta, \omega). \tag{10}$$

While deriving the conditional likelihood in Equation 4, we assumed that summary statistics for the mediators, and summary statistics for the exposure and outcome come from independent samples with no sample overlap. Furthermore, genetic instruments for the mediators are independent from genetic instruments for the exposure. Thus all the components of Equation 4 are independent. This product of independent Gaussian likelihoods is itself a Gaussian:

$$\Pr(\underbrace{\text{sumstats}}_{\begin{pmatrix}\hat{b}\\ \hat{c}\\ \mathrm{vec}(\hat{C})\end{pmatrix}} \mid \alpha_D, \gamma, \delta, \omega) = \mathcal{N}\left(\begin{pmatrix}\hat{B}\delta\\ \hat{\beta}\cdot(\alpha_D + \gamma'\delta)\\ \mathrm{vec}(\hat{\beta}\gamma')\end{pmatrix}, \begin{pmatrix}\Lambda_{s_b^2} & 0 & 0\\ 0 & \Lambda_{s_c^2} & 0\\ 0 & 0 & S_C \otimes I_m\end{pmatrix}\right),$$

where I_m refers to the identity matrix of size $m \times m$. By the same reasoning, the joint distribution under the integral in Equation 10—the product of conditional Gaussian and marginal Gaussian—is itself a multivariate Gaussian.

To get rid of the mediation effects in Equation 10—instead of going directly under the integral to complete the square with respect to the parameters we wish to integrate out—we use the known facts that the marginal distribution of a multivariate Gaussian is a Gaussian, and a Gaussian is defined by its mean and variance.

By the law of total expectation, the means of all the components of the summary statistics vector can be expressed as follows (note that we treat the summary statistics as fixed):

$$\begin{aligned}
\mathrm{E}(\hat{b} \mid \alpha_D) &= \mathrm{E}\big[\mathrm{E}(\hat{b} \mid \alpha_D, \delta)\big] = \mathrm{E}\big(\hat{B}\delta\big) = \hat{B} \cdot \mathrm{E}(\delta) = 0, \\
\mathrm{E}(\hat{c} \mid \alpha_D) &= \mathrm{E}\big[\mathrm{E}(\hat{c} \mid \alpha_D, \gamma, \delta)\big] = \mathrm{E}\big(\hat{\beta} \cdot (\alpha_D + \omega)\big) = \hat{\beta} \cdot \big(\alpha_D + \mathrm{E}(\omega)\big) = \hat{\beta} \cdot (\alpha_D + k\sigma_{\gamma,\delta}) \\
\mathrm{E}\big(\mathrm{vec}(\hat{C}) \mid \alpha_D\big) &= \mathrm{E}\big[\mathrm{E}\big(\mathrm{vec}(\hat{C}) \mid \alpha_D, \gamma\big)\big] = \mathrm{E}\big(\mathrm{vec}(\hat{\beta}\gamma')\big) = \mathrm{vec}\big(\hat{\beta} \cdot \mathrm{E}(\gamma')\big) = 0.
\end{aligned}$$

Similarly, we apply the law of total variance to find the variance of the components:

$$\begin{aligned}
\mathrm{Var}(\hat{b} \mid \alpha_D) &= \mathrm{E}\big[\mathrm{Var}(\hat{b} \mid \alpha_D, \delta)\big] + \mathrm{Var}\big[\mathrm{E}(\hat{b} \mid \alpha_D, \delta)\big] = \mathrm{E}\big(\Lambda_{s_b^2}\big) + \mathrm{Var}(\hat{B}\delta) \\
&= \Lambda_{\mu_{s_b^2}} + \sigma_\delta^2 \hat{B}\hat{B}', \\
\mathrm{Var}(\hat{c} \mid \alpha_D) &= \mathrm{E}\big[\mathrm{Var}(\hat{c} \mid \alpha_D, \gamma, \delta)\big] + \mathrm{Var}\big[\mathrm{E}(\hat{c} \mid \alpha_D, \gamma, \delta)\big] = \mathrm{E}\big(\Lambda_{s_c^2}\big) + \mathrm{Var}\big(\hat{\beta} \cdot (\alpha_D + \omega)\big) \\
&= \Lambda_{\mu_{s_c^2}} + \sigma_\omega^2 \hat{\beta}\hat{\beta}', \\
\mathrm{Var}\big(\mathrm{vec}(\hat{C}) \mid \alpha_D\big) &= \mathrm{E}\big[\mathrm{Var}\big(\mathrm{vec}(\hat{C}) \mid \alpha_D, \gamma\big)\big] + \mathrm{Var}\big[\mathrm{E}\big(\mathrm{vec}(\hat{C}) \mid \alpha_D, \gamma\big)\big] \\
&= \mathrm{E}\big(S_C \otimes I_m\big) + \mathrm{Var}\big(\mathrm{vec}(\hat{\beta}\gamma')\big) \\
&= \big(\mathrm{E}(S_C) \otimes I_m\big) + \sigma_\gamma^2 \cdot (I_k \otimes \hat{\beta}\hat{\beta}'),
\end{aligned}$$

where we used the properties of Kronecker product and vectorization to express $\mathrm{vec}(\hat{\beta}\gamma')$ and consequently its variance:

$$\begin{aligned}
\mathrm{vec}(\hat{\beta}\gamma') = \mathrm{vec}(\hat{\beta}\gamma' \cdot I_k) = (I_k \otimes \hat{\beta}) \cdot \mathrm{vec}(\gamma') = (I_k \otimes \hat{\beta}) \cdot \gamma, \\
\mathrm{Var}\big(\mathrm{vec}(\hat{\beta}\gamma')\big) = (I_k \otimes \hat{\beta}) \cdot \mathrm{Var}(\gamma) \cdot (I_k \otimes \hat{\beta})' = \sigma_\gamma^2 \cdot (I_k \otimes \hat{\beta}\hat{\beta}').
\end{aligned}$$

The expected values for the variance components defined in Equations 5, 6 and 7 are as follows (note

that we used Equation 8 for $\mathrm{E}(\omega)$ and Equation 9 for $\mathrm{Var}(\omega)$):

$$\begin{aligned}
\mu_{s_b^2} &:= \mathrm{E}(s_b^2) = \mathrm{E}\left(\frac{1}{n_Y} + \sigma_b^2 + \frac{1}{n_M}\cdot\delta'\cdot\Sigma\cdot\delta\right) = \frac{1}{n_Y} + \sigma_b^2 + \frac{1}{n_M}\cdot\mathrm{E}(\delta'\cdot\Sigma\cdot\delta)\\
&= \frac{1}{n_Y} + \sigma_b^2 + \frac{1}{n_M}\cdot\sum_{i,j=1}^{k}\mathrm{E}(\delta_i\delta_j)\Sigma_{i,j} = \frac{1}{n_Y} + \sigma_b^2 + \frac{1}{n_M}\cdot\sum_{i}^{k}\sigma_\delta^2\Sigma_{ii}\\
&= \frac{1}{n_Y} + \sigma_b^2 + \frac{1}{n_M}\cdot\sigma_\delta^2\cdot\mathrm{tr}(\Sigma),\\
\mu_{s_c^2} &:= \mathrm{E}(s_c^2) = \mathrm{E}\left(\frac{1}{n_Y} + \sigma_c^2 + \frac{(\alpha_D+\gamma'\delta)^2}{n_X}\right)\\
&= \frac{1}{n_Y} + \sigma_c^2 + \frac{1}{n_X}\Big(\alpha_D^2 + 2\alpha_D\cdot\mathrm{E}(\gamma'\delta) + \mathrm{E}\left((\gamma'\delta)^2\right)\Big)\\
&= \frac{1}{n_Y} + \sigma_c^2 + \frac{1}{n_X}\Big(\alpha_D^2 + 2\alpha_D\cdot\mathrm{E}(\omega) + \mathrm{Var}(\omega) + \big(\mathrm{E}(\omega)\big)^2\Big)\\
&= \frac{1}{n_Y} + \sigma_c^2 + \frac{1}{n_X}\Big(\sigma_\omega^2 + (\alpha_D + k\sigma_{\gamma,\delta})^2\Big),\\
\mathrm{E}(S_C) &= \mathrm{E}\left(\left(\frac{1}{n_M}+\sigma_C^2\right)\cdot\Sigma + \frac{\gamma\cdot\gamma'}{n_X}\right) = \left(\frac{1}{n_M}+\sigma_C^2\right)\cdot\Sigma + \frac{1}{n_X}\cdot\mathrm{E}(\gamma\gamma')\\
&= \left(\frac{1}{n_M}+\sigma_C^2\right)\cdot\Sigma + \frac{1}{n_X}\cdot\Lambda_{\sigma_\gamma^2}.
\end{aligned}$$

The form of $\mathrm{E}(S_C)$ makes it possible to use the properties of Kronecker product to simplify the expression for $\mathrm{Var}\big(\mathrm{vec}(\hat{C})\mid\alpha_D\big)$:

$$\begin{aligned}
\mathrm{Var}\big(\mathrm{vec}(\hat{C})\mid\alpha_D\big) &= \mathrm{E}(S_C)\otimes I_m + \sigma_\gamma^2\cdot(I_k\otimes\hat{\beta}\hat{\beta}')\\
&= \left(\left(\frac{1}{n_M}+\sigma_C^2\right)\cdot\Sigma + \frac{1}{n_X}\cdot\Lambda_{\sigma_\gamma^2}\right)\otimes I_m + \sigma_\gamma^2\cdot(I_k\otimes\hat{\beta}\hat{\beta}')\\
&= \left(\left(\frac{1}{n_M}+\sigma_C^2\right)\cdot\Sigma\right)\otimes I_m + \left(\frac{1}{n_X}\cdot\Lambda_{\sigma_\gamma^2}\right)\otimes I_m + I_k\otimes\left(\sigma_\gamma^2\hat{\beta}\hat{\beta}'\right)\\
&= \Sigma\otimes\Lambda_{s_M^2} + I_k\otimes\left(\frac{1}{n_X}\sigma_\gamma^2\cdot I_m\right) + I_k\otimes\left(\sigma_\gamma^2\hat{\beta}\hat{\beta}'\right)\\
&= \Sigma\otimes\Lambda_{s_M^2} + I_k\otimes\sigma_\gamma^2\left(\Lambda_{n_X^{-1}} + \hat{\beta}\hat{\beta}'\right),\ \text{where } s_M^2 := \frac{1}{n_M}+\sigma_C^2.
\end{aligned}$$

Finally, we use the law of total covariance to find the covariance between all the components:

$$\begin{aligned}
\mathrm{Cov}\big(\hat{b},\hat{c}\mid\alpha_D\big) &= \mathrm{E}\big[\mathrm{Cov}(\hat{b},\hat{c}\mid\alpha_D,\delta,\omega)\big] + \mathrm{Cov}\big[\mathrm{E}(\hat{b}\mid\alpha_D,\delta),\mathrm{E}(\hat{c}\mid\alpha_D,\omega)\big]\\
&= 0 + \mathrm{Cov}\big(\hat{B}\delta,\hat{\beta}(\alpha_D+\omega)\big)\\
&= \hat{B}\cdot\mathrm{Cov}(\delta,\omega)\cdot\hat{\beta}'\\
&= 0,
\end{aligned}$$

$$\begin{aligned}
\mathrm{Cov}\big(\hat{b},\mathrm{vec}(\hat{C})\big)\mid\alpha_D\big) &= \mathrm{E}\Big[\mathrm{Cov}\big(\hat{b},\mathrm{vec}(\hat{C})\big)\mid\alpha_D,\gamma,\delta\big)\Big] + \mathrm{Cov}\Big[\mathrm{E}(\hat{b}\mid\alpha_D,\delta),\mathrm{E}\big(\mathrm{vec}(\hat{C})\mid\alpha_D,\gamma\big)\Big]\\
&= 0 + \mathrm{Cov}\big(\hat{B}\delta,\mathrm{vec}(\hat{\beta}\gamma')\big)\\
&= \mathrm{Cov}\big(\hat{B}\delta,(I_k\otimes\hat{\beta})\cdot\gamma\big)
\end{aligned}$$

$$
\begin{aligned}
&= (\hat{B} \otimes 1) \cdot \mathrm{Cov}(\delta, \gamma) \cdot (I_k \otimes \hat{\beta})' \\
&= \sigma_{\gamma,\delta} \cdot (\hat{B} \otimes \hat{\beta}'),
\end{aligned}
$$

$$
\begin{aligned}
\mathrm{Cov}\big(\hat{c}, \mathrm{vec}\big(\hat{C}\big) \mid \alpha_D\big) &= \mathrm{E}\Big[\mathrm{Cov}\big(\hat{c}, \mathrm{vec}\big(\hat{C}\big) \mid \alpha_D, \gamma, \omega\big)\Big] + \mathrm{Cov}\Big[\mathrm{E}(\hat{c} \mid \alpha_D, \omega), \mathrm{E}\big(\mathrm{vec}\big(\hat{C}\big) \mid \alpha_D, \gamma\big)\Big] \\
&= 0 + \mathrm{Cov}\big(\hat{\beta} \cdot (\alpha_D + \omega), \mathrm{vec}(\hat{\beta}\gamma')\big) \\
&= \mathrm{Cov}\big(\hat{\beta}\omega, (I_k \otimes \hat{\beta}) \cdot \gamma\big) \\
&= \hat{\beta} \cdot \mathrm{Cov}(\omega, \gamma) \cdot (I_k \otimes \hat{\beta})' \\
&= 0.
\end{aligned}
$$

The marginal likelihood $\Pr(\text{sumstats} \mid \alpha_D)$ in Equation 10 is thus

$$
\mathcal{N}\left(\begin{pmatrix} 0 \\ \hat{\beta}(\alpha_D + k\sigma_{\gamma,\delta}) \\ 0 \end{pmatrix}, \begin{pmatrix} \Lambda_{\mu_{s_b^2}} + \sigma_\delta^2 \hat{B}\hat{B}' & 0 & \sigma_{\gamma,\delta} \cdot (\hat{B} \otimes \hat{\beta}') \\ 0 & \Lambda_{\mu_{s_c^2}} + \sigma_w^2 \hat{\beta}\hat{\beta}' & 0 \\ \sigma_{\gamma,\delta} \cdot (\hat{B}' \otimes \hat{\beta}) & 0 & \Sigma \otimes \Lambda_{s_M^2} + I_k \otimes \sigma_\gamma^2 \left(\Lambda_{n_X^{-1}} + \hat{\beta}\hat{\beta}'\right) \end{pmatrix}\right). \tag{11}
$$

Optimizing the likelihood

The optimization of the likelihood requires the calculation of the precision matrix – matrix inverse of the covariance matrix – and the determinant. For an $n \times n$ matrix, the algorithmic complexity of either operation is at best $O(n^{2.37286})$ [6] (the exact complexity depends on the matrix multiplication algorithm invoked by the software that is used to perform the operations). The covariance matrix in the derived marginal likelihood (Equation 11) is $(mk + m + l) \times (mk + m + l)$, where the number of instruments of a complex polygenic trait can easily be $m > 100$, the number of potential mediators to consider in omics layers can be $k > 1000$, and the total number of mediator instruments l can be even bigger. Thus it will be virtually infeasible to optimize the likelihood in its current form.

Note that the $\hat{c}$-component in the derived likelihood is independent of the other components. Thus we can easily simplify the likelihood as follows:

$$
\mathcal{N}\left(0, \begin{pmatrix} \Lambda_{\mu_{s_b^2}} + \sigma_\delta^2 \hat{B}\hat{B}' & \sigma_{\gamma,\delta} \cdot (\hat{B} \otimes \hat{\beta}') \\ \sigma_{\gamma,\delta} \cdot (\hat{B}' \otimes \hat{\beta}) & \Sigma \otimes \Lambda_{s_M^2} + I_k \otimes \sigma_\gamma^2 \left(\Lambda_{n_X^{-1}} + \hat{\beta}\hat{\beta}'\right) \end{pmatrix}\right) \cdot \mathcal{N}\left(\hat{\beta}(\alpha_D + k\sigma_{\gamma,\delta}), \Lambda_{\mu_{s_c^2}} + \sigma_w^2 \hat{\beta}\hat{\beta}'\right). \tag{12}
$$

Now, the covariance matrix in the first Gaussian is $(mk + l) \times (mk + l)$ and the covariance matrix for the second Gaussian is $m \times m$. This is already slightly better than before but we can improve even further.

Simplifying the likelihood function for $\hat{c}$

Remember that we used the notation Λ_x to describe diagonal matrices with x on the diagonal. Thus, by the Sherman-Morrison formula, we can express the precision matrix of the second Gaussian in Equation 12 as

$$
\Sigma_2^{-1} = \left(\Lambda_{\mu_{s_c^2}} + \sigma_\omega^2 \hat{\beta}\hat{\beta}'\right)^{-1} = \Lambda_{\mu_{s_c^2}^{-1}} - \frac{\Lambda_{\mu_{s_c^2}^{-1}} \cdot \sigma_\omega^2 \hat{\beta}\hat{\beta}' \cdot \Lambda_{\mu_{s_c^2}^{-1}}}{1 + \hat{\beta}' \cdot \Lambda_{\mu_{s_c^2}^{-1}} \cdot \sigma_\omega^2 \hat{\beta}}
$$

$$= \mu_{s_c^2}^{-1} I_m - \frac{\sigma_\omega^2}{\mu_{s_c^2}^2 \left(1 + \frac{\sigma_\omega^2}{\mu_{s_c^2}} \hat{\beta}' \hat{\beta}\right)} \hat{\beta}\hat{\beta}' = \frac{1}{\mu_{s_c^2}} I_m - \frac{1}{\mu_{s_c^2}} \left(\frac{\sigma_\omega^2}{\mu_{s_c^2} + \sigma_\omega^2 \hat{\beta}'\hat{\beta}} \right) \hat{\beta}\hat{\beta}'$$

$$= \frac{1}{\mu_{s_c^2}} \left(I_m - \xi_{\hat{c}} \hat{\beta}\hat{\beta}' \right), \text{ where } \xi_{\hat{c}} = \frac{\sigma_\omega^2}{\mu_{s_c^2} + \sigma_\omega^2 \hat{\beta}'\hat{\beta}}. \tag{13}$$

The complexity of inverting the covariance matrix Σ_2 is now $O(m^2)$, i.e. quadratic in m. Similarly, using the matrix determinant lemma, we can find the determinant of Σ_2 in $O(m)$ time as

$$\begin{aligned}
\det(\Sigma_2) &= \det\left(\Lambda_{\mu_{s_c^2}} + \sigma_\omega^2 \hat{\beta}\hat{\beta}'\right) = \left(1 + \sigma_\omega^2 \hat{\beta}' \Lambda_{\mu_{s_c^2}^{-1}} \hat{\beta}\right) \cdot \det\left(\Lambda_{\mu_{s_c^2}}\right) \\
&= \left(1 + \frac{\sigma_\omega^2}{\mu_{s_c^2}} \hat{\beta}'\hat{\beta}\right) \cdot \det\left(\mu_{s_c^2} I_m\right) = \mu_{s_c^2}^m \cdot \frac{\mu_{s_c^2} + \sigma_\omega^2 \hat{\beta}'\hat{\beta}}{\mu_{s_c^2}} \\
&= \frac{\mu_{s_c^2}^{m-1} \sigma_\omega^2}{\xi_{\hat{c}}}.
\end{aligned}$$

The likelihood simplifies further if we consider that the precision matrix in Equation 13 is multiplied from left and right by a vector. Let $\xi_D := (\alpha_D + k\sigma_{\gamma,\delta})$ such that the mean of $\hat{c}$ is $\mu_2 = \xi_D \hat{\beta}$, then:

$$\begin{aligned}
\Pr(\hat{c} \mid \alpha_D) &= \frac{1}{\sqrt{(2\pi)^m \cdot \det(\Sigma_2)}} \exp\left(-\frac{1}{2} (\hat{c} - \mu_2)' \Sigma_2^{-1} (\hat{c} - \mu_2) \right) \\
&= \frac{1}{\sqrt{(2\pi)^m \cdot \det(\Sigma_2)}} \exp\left(-\frac{1}{2\mu_{s_c^2}} \left(\hat{c} - \xi_D \hat{\beta}\right)' \left(I_m - \xi_{\hat{c}} \hat{\beta}\hat{\beta}'\right) \left(\hat{c} - \xi_D \hat{\beta}\right) \right) \\
&= \frac{1}{\sqrt{(2\pi)^m \cdot \det(\Sigma_2)}} \exp\left(\frac{\xi_{\hat{c}}}{2\mu_{s_c^2}} \left(\hat{c} - \xi_D \hat{\beta}\right)' \hat{\beta}\hat{\beta}' \left(\hat{c} - \xi_D \hat{\beta}\right) - \frac{1}{2\mu_{s_c^2}} \left(\hat{c} - \xi_D \hat{\beta}\right)' \left(\hat{c} - \xi_D \hat{\beta}\right) \right) \\
&= \frac{1}{\sqrt{(2\pi)^m \cdot \det(\Sigma_2)}} \exp\left(\frac{\xi_{\hat{c}}}{2\mu_{s_c^2}} \left(\hat{c}'\hat{\beta} - \xi_D \hat{\beta}'\hat{\beta}\right)^2 - \frac{1}{2\mu_{s_c^2}} \left(\hat{c} - \xi_D \hat{\beta}\right)' \left(\hat{c} - \xi_D \hat{\beta}\right) \right) \\
&= \frac{1}{\sqrt{\left(2\pi\right)^m \mu_{s_c^2}^{m-1} \sigma_\omega^2 \xi_{\hat{c}}^{-1}}} \exp\left(\frac{\xi_{\hat{c}}}{2\mu_{s_c^2}} \left(\hat{c}'\hat{\beta} - \xi_D \hat{\beta}'\hat{\beta}\right)^2 - \frac{1}{2\mu_{s_c^2}} \left(\hat{c} - \xi_D \hat{\beta}\right)' \left(\hat{c} - \xi_D \hat{\beta}\right) \right).
\end{aligned} \tag{14}$$

While there are no big matrix multiplications remaining in the likelihood in Equation 14, computing the m-power terms might lead to integer overflow if m is large. Thus we take a logarithm:

$$\begin{aligned}
\log \Pr(\hat{c} \mid \alpha_D) = &-\frac{1}{2}\Big(m \cdot \log(2\pi) + (m-1) \cdot \log\left(\mu_{s_c^2}\right) + \log\left(\sigma_\omega^2\right) - \log\left(\xi_{\hat{c}}\right) \Big) \\
&+ \frac{\xi_{\hat{c}}}{2\mu_{s_c^2}} \left(\hat{c}'\hat{\beta} - \xi_D \hat{\beta}'\hat{\beta}\right)^2 - \frac{1}{2\mu_{s_c^2}} \left(\hat{c} - \xi_D \hat{\beta}\right)' \left(\hat{c} - \xi_D \hat{\beta}\right).
\end{aligned} \tag{15}$$

This completes the simplification of the likelihood of $\hat{c}$.

Simplifying the likelihood function for $\begin{pmatrix}\hat{b}\\ \text{vec}(\hat{C})\end{pmatrix}$

By the 2×2 block matrix inversion formula, we can express the precision matrix of the first Gaussian in Equation 12 as:

$$\begin{aligned}\Sigma_1^{-1} &= \begin{pmatrix}L_{11} & L_{12}\\ L_{21} & L_{22}\end{pmatrix} = \begin{pmatrix}A & B\\ C & D\end{pmatrix}^{-1}\\ &= \begin{pmatrix}(A-BD^{-1}C)^{-1} & -(A-BD^{-1}C)^{-1}BD^{-1}\\ -D^{-1}C(A-BD^{-1}C)^{-1} & D^{-1}+D^{-1}C(A-BD^{-1}C)^{-1}BD^{-1}\end{pmatrix}.\end{aligned} \tag{16}$$

The unknown elements in this formula are D^{-1} and $L_{11}=(A-BD^{-1}C)^{-1}$. As D is an $mk\times mk$ Kronecker sum (a sum of two Kronecker products), D^{-1} does not have a nice general form and can be very expensive to compute. In order to simplify the computation of D^{-1}, we will force D into a nicer form by assuming that Σ is an identity matrix—our simulations show that this is a reasonable assumption to make (Fig. S1). Therefore,

$$D = I_k\otimes\Lambda_{s_M^2} + I_k\otimes\sigma_\gamma^2\left(\Lambda_{n_X^{-1}}+\hat{\beta}\hat{\beta}'\right) = I_k\otimes\left(\Lambda_{s_C^2}+\sigma_\gamma^2\hat{\beta}\hat{\beta}'\right),\ \text{where } s_C^2 \coloneqq s_M^2+\frac{1}{n_X}\sigma_\gamma^2.$$

We can make use of Kronecker product's inversion property and the result we derived in Equation 13 to express the inverse as

$$\begin{aligned}D^{-1} &= \left(I_k\otimes\left(\Lambda_{s_C^2}+\sigma_\gamma^2\hat{\beta}\hat{\beta}'\right)\right)^{-1} = I_k\otimes\left(\Lambda_{s_C^2}+\sigma_\gamma^2\hat{\beta}\hat{\beta}'\right)^{-1}\\ &= I_k\otimes\frac{1}{s_C^2}\left(I_m-\xi_{\hat{b}}\hat{\beta}\hat{\beta}'\right),\ \text{where } \xi_{\hat{b}} \coloneqq \frac{\sigma_\gamma^2}{s_C^2+\sigma_\gamma^2\hat{\beta}'\hat{\beta}}.\end{aligned}$$

The top-left block of the precision matrix in Equation 16 can now be expressed as

$$\begin{aligned}L_{11} &= (A-BD^{-1}C)^{-1}\\ &= \left(\Lambda_{\mu_{s_b^2}}+\sigma_\delta^2\hat{B}\hat{B}'-\sigma_{\gamma,\delta}\left(\hat{B}\otimes\hat{\beta}'\right)\left(I_k\otimes\frac{1}{s_C^2}\left(I_m-\xi_{\hat{b}}\hat{\beta}\hat{\beta}'\right)\right)\left(\hat{B}'\otimes\hat{\beta}\right)\sigma_{\gamma,\delta}\right)^{-1}\\ &= \left(\Lambda_{\mu_{s_b^2}}+\sigma_\delta^2\hat{B}\hat{B}'-\frac{\sigma_{\gamma,\delta}^2}{s_C^2}\hat{B}\hat{B}'\otimes\left(\hat{\beta}'\left(I_m-\xi_{\hat{b}}\hat{\beta}\hat{\beta}'\right)\hat{\beta}\right)\right)^{-1}\\ &= \left(\Lambda_{\mu_{s_b^2}}+\sigma_\delta^2\hat{B}\hat{B}'-\frac{\sigma_{\gamma,\delta}^2}{s_C^2}\hat{\beta}'\hat{\beta}\left(1-\xi_{\hat{b}}\hat{\beta}'\hat{\beta}\right)\cdot\hat{B}\hat{B}'\right)^{-1}\\ &= \left(\Lambda_{\mu_{s_b^2}}+\xi_1\hat{B}\hat{B}'\right)^{-1},\ \text{where } \xi_1 \coloneqq \sigma_\delta^2-\frac{\sigma_{\gamma,\delta}^2}{s_C^2}\hat{\beta}'\hat{\beta}\left(1-\xi_{\hat{b}}\hat{\beta}'\hat{\beta}\right).\end{aligned} \tag{17}$$

The inversion in Equation 17 is over an $l\times l$ matrix. We can use the Woodbury matrix identity to have us take an inverse of a $k\times k$ matrix instead:

$$\begin{aligned}L_{11} &= \left(\Lambda_{\mu_{s_b^2}}+\xi_1\hat{B}\hat{B}'\right)^{-1} = \Lambda_{\mu_{s_b^2}^{-1}}-\Lambda_{\mu_{s_b^2}^{-1}}\hat{B}\left(\Lambda_{\xi_1^{-1}}+\hat{B}'\Lambda_{\mu_{s_b^2}^{-1}}\hat{B}\right)^{-1}\hat{B}'\Lambda_{\mu_{s_b^2}^{-1}}\\ &= \frac{1}{\mu_{s_b^2}}\left(I_l-\frac{1}{\mu_{s_b^2}}\hat{B}L_{11}^*\hat{B}'\right),\ \text{where } L_{11}^* \coloneqq \left(\frac{1}{\xi_1}I_k+\frac{1}{\mu_{s_b^2}}\hat{B}'\hat{B}\right)^{-1}.\end{aligned} \tag{18}$$

Calculating L_{11} using Equation 18 instead of Equation 17 is faster whenever mediators have on average 1.5 instruments. The remaining blocks of the precision matrix in Equation 16 can be calculated in terms of L_{11} as follows:

$$\begin{aligned}
L_{12} &= -L_{11}BD^{-1} \\
&= -\sigma_{\gamma,\delta}L_{11}\left(\hat{B}\otimes\hat{\beta}'\right)\left(I_k\otimes\frac{1}{s_C^2}\left(I_m-\xi_{\hat{b}}\hat{\beta}\hat{\beta}'\right)\right) \\
&= -\frac{\sigma_{\gamma,\delta}}{s_C^2}L_{11}\left(\hat{B}\otimes\left(\hat{\beta}'\left(I_m-\xi_{\hat{b}}\hat{\beta}\hat{\beta}'\right)\right)\right) \\
&= -\frac{\sigma_{\gamma,\delta}}{s_C^2}L_{11}\left(\hat{B}\otimes\left(\left(1-\xi_{\hat{b}}\hat{\beta}'\hat{\beta}\right)\hat{\beta}'\right)\right) \\
&= \xi_2L_{11}\left(\hat{B}\otimes\hat{\beta}'\right),\ \text{where } \xi_2 := -\frac{\sigma_{\gamma,\delta}}{s_C^2}\left(1-\xi_{\hat{b}}\hat{\beta}'\hat{\beta}\right), \qquad (19) \\
L_{21} &= L_{12}' = \xi_2\left(\hat{B}'\otimes\hat{\beta}\right)L_{11}, \qquad (20) \\
L_{22} &= D^{-1}-D^{-1}CL_{12} = D^{-1}-\xi_2D^{-1}CL_{11}\left(\hat{B}\otimes\hat{\beta}'\right) = D^{-1}+\xi_2L_{21}\left(\hat{B}\otimes\hat{\beta}'\right) \\
&= \frac{1}{s_C^2}\left(I_k\otimes\left(I_m-\xi_{\hat{b}}\hat{\beta}\hat{\beta}'\right)\right)+\xi_2^2\left(\hat{B}'\otimes\hat{\beta}\right)L_{11}\left(\hat{B}\otimes\hat{\beta}'\right). \qquad (21)
\end{aligned}$$

Simplifying the determinant

The general formula for a determinant of a 2×2 block matrix is

$$\det(\Sigma_1) = \det\begin{pmatrix} A & B \\ C & D\end{pmatrix} = \det(D)\cdot\det(A-BD^{-1}C). \qquad (22)$$

We can find the determinants on the right-hand side of Equation 22 by applying Kronecker product and determinant properties, matrix determinant lemma for $\det(D)$, and Weinstein-Aronszajn identity for $\det(A-BD^{-1}C)$ as follows:

$$\begin{aligned}
\det(D) &= \det\left(I_k\otimes\left(\Lambda_{s_C^2}+\sigma_\gamma^2\hat{\beta}\hat{\beta}'\right)\right) = \left(\det(I_k)\right)^m\cdot\left(\det\left(s_C^2I_m+\sigma_\gamma^2\hat{\beta}\hat{\beta}'\right)\right)^k \\
&= 1\cdot\left(\left(1+\frac{\sigma_\gamma^2}{s_C^2}\hat{\beta}'I_m\hat{\beta}\right)\det\left(s_C^2I_m\right)\right)^k \\
&= s_C^{2mk}\cdot\xi_3^k,\quad\text{where } \xi_3 = 1+\frac{\sigma_\gamma^2}{s_C^2}\hat{\beta}'\hat{\beta}, \\
\det\left(A-BD^{-1}C\right) &= \det\left(\Lambda_{\mu_{s_b^2}}+\xi_1\hat{B}\hat{B}'\right) = \mu_{s_b^2}^l\cdot\det\left(I_l+\frac{\xi_1}{\mu_{s_b^2}}\hat{B}\hat{B}'\right) \\
&= \mu_{s_b^2}^l\cdot\det\left(I_k+\frac{\xi_1}{\mu_{s_b^2}}\hat{B}'\hat{B}\right).
\end{aligned}$$

Thus the determinant in Equation 22 simplifies to

$$\det(\Sigma_1) = \mu_{s_b^2}^l\cdot s_C^{2mk}\cdot\xi_3^k\cdot\det\left(I_k+\frac{\xi_1}{\mu_{s_b^2}}\hat{B}'\hat{B}\right), \qquad (23)$$

which requires taking a determinant only over a $k\times k$ matrix.

Expressing the likelihood

Having found simple forms for the precision matrix in Equation 18 (or Equation 17) and for the determinant in Equation 23, we can plug these into the first multivariate Gaussian in Equation 12 to simplify the joint likelihood of $\hat{b}$ and $\mathrm{vec}(\hat{C})$:

$$\Pr\begin{pmatrix}\hat{b}\\ \mathrm{vec}(\hat{C})\end{pmatrix} = \frac{1}{\sqrt{(2\pi)^{mk+l}\cdot\det(\Sigma_1)}}\exp\left(-\frac{1}{2}\begin{pmatrix}\hat{b}\\ \mathrm{vec}(\hat{C})\end{pmatrix}'\Sigma_1^{-1}\begin{pmatrix}\hat{b}\\ \mathrm{vec}(\hat{C})\end{pmatrix}\right)$$
$$= \frac{\exp\left(-\frac{1}{2}\hat{b}'L_{11}\hat{b} - \hat{b}'L_{12}\mathrm{vec}(\hat{C}) - \frac{1}{2}\mathrm{vec}(\hat{C})L_{22}\mathrm{vec}(\hat{C})\right)}{\sqrt{(2\pi)^{mk+l}\cdot\mu_{s_{\hat{b}}^2}^{l}\cdot s_C^{2mk}\cdot\xi_3^k\cdot\det\left(I_k + \frac{\xi_1}{\mu_{s_{\hat{b}}^2}}\hat{B}'\hat{B}\right)}}. \tag{24}$$

To simplify the exponent, we can express L_{12} and L_{22} in terms of L_{11} as derived in Equation 19 and Equation 21, and make use of the relationship between Kronecker products and vec-operations by which $(Z'\otimes X)\mathrm{vec}(Y) = \mathrm{vec}(XYZ)$ for matrices X, Y, Z of appropriate dimensions:

$$\hat{b}'L_{12}\mathrm{vec}(\hat{C}) = \xi_2\hat{b}'L_{11}\left(\hat{B}\otimes\hat{\beta}'\right)\mathrm{vec}(\hat{C}) = \xi_2\hat{b}'L_{11}\mathrm{vec}(\hat{\beta}'\hat{C}\hat{B}')$$
$$= \xi_2\hat{b}'L_{11}\hat{B}\hat{C}'\hat{\beta},$$
$$\mathrm{vec}(\hat{C})'L_{22}\mathrm{vec}(\hat{C}) = \mathrm{vec}(\hat{C})'\left(\frac{1}{s_C^2}\left(I_k\otimes\left(I_m - \xi_{\hat{b}}\hat{\beta}\hat{\beta}'\right)\right) + \xi_2^2\left(\hat{B}'\otimes\hat{\beta}\right)L_{11}\left(\hat{B}\otimes\hat{\beta}'\right)\right)\mathrm{vec}(\hat{C})$$
$$= \frac{1}{s_C^2}\mathrm{vec}(\hat{C})'\mathrm{vec}\left(\left(I_m - \xi_{\hat{b}}\hat{\beta}\hat{\beta}'\right)\hat{C}I_k\right) + \xi_2^2\mathrm{vec}\left(\hat{\beta}'\hat{C}\hat{B}'\right)'L_{11}\mathrm{vec}\left(\hat{\beta}'\hat{C}\hat{B}'\right)$$
$$= \frac{1}{s_C^2}\mathrm{vec}(\hat{C})'\mathrm{vec}\left(\hat{C} - \xi_{\hat{b}}\hat{\beta}\hat{\beta}'\hat{C}\right) + \xi_2^2(\hat{B}\hat{C}'\hat{\beta})'L_{11}\hat{B}\hat{C}'\hat{\beta},$$

Plugging these into the exponent of Equation 24 together with the first summand already there permits us to complete the square and simplify the likelihood as follows:

$$\Pr\begin{pmatrix}\hat{b}\\ \mathrm{vec}(\hat{C})\end{pmatrix} = \frac{\exp\left(-\frac{1}{2}\left(\left(\hat{b} + \xi_2\hat{B}\hat{C}'\hat{\beta}\right)'L_{11}\left(\hat{b} + \xi_2\hat{B}\hat{C}'\hat{\beta}\right) + \frac{1}{s_C^2}\mathrm{vec}(\hat{C})'\mathrm{vec}\left(\hat{C} - \xi_{\hat{b}}\hat{\beta}\hat{\beta}'\hat{C}\right)\right)\right)}{\sqrt{(2\pi)^{mk+l}\cdot\mu_{s_{\hat{b}}^2}^{l}\cdot s_C^{2mk}\cdot\xi_3^k\cdot\det\left(I_k + \frac{\xi_1}{\mu_{s_{\hat{b}}^2}}\hat{B}'\hat{B}\right)}},$$

where L_{11} can be expressed by Equation 17 or Equation 18. To prevent integer overflow in calculating this likelihood, it is useful to take a logarithm of it:

$$\log\Pr\begin{pmatrix}\hat{b}\\ \mathrm{vec}(\hat{C})\end{pmatrix} = -\frac{1}{2}\left(\left(\hat{b} + \xi_2\hat{B}\hat{C}'\hat{\beta}\right)'L_{11}\left(\hat{b} + \xi_2\hat{B}\hat{C}'\hat{\beta}\right) + \frac{1}{s_C^2}\mathrm{vec}(\hat{C})'\mathrm{vec}\left(\hat{C} - \xi_{\hat{b}}\hat{\beta}\hat{\beta}'\hat{C}\right)\right)$$
$$-\frac{1}{2}\left((mk+l)\log(2\pi) + l\log\left(\mu_{s_{\hat{b}}^2}\right) + mk\log\left(s_C^2\right) + k\log\left(\xi_3\right) + \log\det\left(I_k + \frac{\xi_1}{\mu_{s_{\hat{b}}^2}}\hat{B}'\hat{B}\right)\right). \tag{25}$$

This completes the simplification of the likelihood of $\begin{pmatrix}\hat{b}\\ \mathrm{vec}(\hat{C})\end{pmatrix}$.

Determining the variance components σ_γ^2 and σ_δ^2

Different strategies can be used to determine the prior variances of mediation effects, σ_γ^2 and σ_δ^2. As opposed to estimating them together with α and $\sigma_{\gamma,\delta}$ by optimizing over the marginal likelihood (Equation 11), we found it can often be more beneficial to estimate them separately and then fix their values for the optimization procedure (Fig. S10).

One possibility is to leverage MVMR to estimate $\widehat{\sigma}_\gamma^2 = \mathrm{Var}\big(\widehat{\gamma}\big)$ and $\widehat{\sigma}_\delta^2 = \mathrm{Var}\big(\widehat{\boldsymbol{\delta}}\big)$. However, this can be dangerous when the mediation effects are estimated with bias.

By default in this paper, we estimated the variance components by maximizing over just the relevant parts of the marginal likelihood:

$$\widehat{\sigma}_\gamma^2 = \underset{\sigma_\gamma^2}{\arg\max}\left\{\log\left(\Pr\left(\widehat{\mathbf{C}}, \widehat{\boldsymbol{\beta}}, \sigma_\gamma^2\right)\right)\right\},$$
$$\widehat{\sigma}_\delta^2 = \underset{\sigma_\delta^2}{\arg\max}\left\{\log\left(\Pr\left(\widehat{\boldsymbol{b}}, \widehat{\mathbf{B}}, \sigma_\delta^2\right)\right)\right\},$$

corresponding to the third and first row in Equation 11, respectively. In doing so, we do not consider the possible correlation between indirect effects $\boldsymbol{\gamma}$ and $\boldsymbol{\delta}$.

Relaxing the assumption of uncorrelated mediators

To reach the simplified likelihood function in Equation 25, we relied on the assumption that the mediator correlation matrix Σ is identity. Unit variances on the main diagonal are ensured by the standardization of mediator summary statistics. However, the assumption of uncorrelated mediators might not be met in many realistic application scenarios of our method, such as in mediation analyses where the mediators are entire omics layers. We have shown in simulations that our method is robust to the violation of this assumption, even when there is strong correlation structure present in Σ (Fig. S1). Nevertheless, we explored options to relax this assumption.

The assumption of uncorrelated mediators can be enforced by performing an orthogonal transformation on the original mediators. A popular strategy for doing so is to perform principal component analysis (PCA). The resulting principal components (PCs) would be uncorrelated and could act as the new mediators. As this would bring about a change of mediator variables, the effect sizes in the matrices $\hat{B}$ and $\hat{C}$ would have to be transformed accordingly.

Let $w = (w_1, w_2, \dots w_k) \in \mathbb{R}^k$ be a weight vector corresponding to the vector of mediators $M = (M_1, M_2, \dots M_k)'$. Any PC – denote it by a random variable P – is a linear combination of the mediators and their weights (also called loadings):

$$P := w'M = \sum_{i=1}^{k} w_i M_i,$$

The summary statistics of association between a genotype G and principal component P are thus

$$\hat{\beta}_{G,P} = \frac{g' \cdot \sum_{i=1}^{k} w_i z_i}{g'g} = \sum_{i=1}^{k} w_i \cdot \frac{g' z_i}{g'g} = \sum_{i=1}^{k} w_i \cdot \hat{\beta}_{G,M_i}, \tag{26}$$

$$\mathrm{Var}(\hat{\beta}_{G,P}) = \mathrm{Var}\left(\sum_{i=1}^{k} w_i \cdot \hat{\beta}_{G,M_i}\right) = \mathrm{Var}\left(w' \cdot \hat{\beta}_{G,M}\right) = w' \cdot \mathrm{Var}\left(\hat{\beta}_{G,M}\right) \cdot w = \frac{1}{N} w' \Sigma w, \tag{27}$$

where g and z_i denote the sample realizations of G and M_i for all $i = 1, 2, \dots k$, and the vector $\hat{\beta}_{G,M}$ comprises the effect sizes $\hat{\beta}_{G,M_i}$ of genotype G on mediator M_i—all available and known to us.

Performing PCA on standardized mediators is equivalent to performing eigenvalue decomposition on $\Sigma = W'\Lambda W$, where W is the matrix of eigenvectors (also called principal directions) and Λ is the diagonal matrix with eigenvalues on the diagonal. The matrix Σ is unknown in reality but corresponds to the cross-trait LD Score (LDSC) regression intercept and can thus be calculated for a variety of traits (also available at HAIL, https://ukbb-rg.hail.is/rg_browser/). Transforming matrices $\hat{B}$ and $\hat{C}$ based on the formula 26 is straightforward:

$$\hat{B}^* = W\hat{B}, \quad \hat{C}^* = W\hat{C}.$$

The PCs are naturally uncorrelated. However, the variance of the mediators would not be equal to 1 anymore, owing to the correlation structure between the mediators. This problem is also reflected in formula 27 by which $\text{Var}(\hat{\beta}_{G,P})$ can differ from N^{-1} if $\Sigma \neq I_k$. Our derivations rely on this assumption together with the assumption of a diagonal Σ. Thus, while PCA can be used to enforce the assumption of uncorrelated mediators, it can break the assumption of unit variance of the mediators in the process. Overall, there does not seem to be any clear gain to transforming the mediators with PCA.

While uncorrelated mediators represent a strong assumption on paper, we have demonstrated the robustness of this assumption (Fig. S1). Generating Σ from real gene expression data of the CoLaus cohort did not add to the bias of the mediation proportion. We saw a minor increase in the bias only when the generated correlation structure was artificially strong. Thus we feel confident to recommend our method even when the assumption of uncorrelated mediators is not met. Nevertheless, pruning the mediators to be nearly uncorrelated remains an option.

Impact of mediator selection on (I-)LiMA bias

We have observed that as the mediator selection becomes less and less stringent the downward bias in the mediation proportion estimation of the (I-)LiMA method becomes more and more pronounced. This phenomenon is caused by two opposing forces acting against each other: (i) As mediator selection becomes milder, more and more real mediators are included, increasing the expectation of $\widehat{MP}$. (ii) As more mediators are included in the model, the $\widehat{\omega}/(\widehat{\omega}+\widehat{\alpha})$ ratio becomes more downward biased.

The impact of the first phenomenon is easy to quantify: the milder the threshold, the more true mediator is included, and hence a greater proportion of the mediation is captured. In our setting, we were well-powered to pick up all true mediators already at $p_T = 10^{-4}$ and we are rather interested in why a downward bias emerges as the threshold gets milder.

This downward bias is caused by the second phenomenon, and can be approximated by the Delta method applied to the expectation of the ratio of two random variables. To isolate the impact of adding more null mediators from adding more true mediators, let us assume that all true mediators are included in the model, and we examine the effect of adding more null mediators

$$E\left[\frac{\widehat{\omega}}{\widehat{\omega}+\widehat{\alpha}}\right] \approx \frac{\omega}{\omega+\alpha} - \frac{\alpha}{(\omega+\alpha)^3}\cdot Var(\widehat{\omega}) + (\omega-\alpha)\cdot\frac{cov(\widehat{\omega},\widehat{\alpha})}{(\omega+\alpha)^3} + \frac{\omega}{(\omega+\alpha)^3}\cdot Var(\widehat{\alpha})$$

Since $Var(\widehat{\omega}) >> Var(\widehat{\alpha})$ by orders of magnitude, the last two terms are negligible compared to the (first and) second terms, hence we ignore them for simplicity and we can substitute the expression for the variance of $\widehat{\omega}$ (Eq. 9) to simplify the expression to

$$E\left[\frac{\widehat{\omega}}{\widehat{\omega}+\widehat{\alpha}}\right] \approx \frac{\omega}{\omega+\alpha} - \frac{\alpha}{(\omega+\alpha)^3}\cdot Var(\widehat{\omega})$$

The variance of $\widehat{\omega}$ was derived above, but (γ_i, δ_i) were assumed to follow a bivariate Gaussian. However, when null mediators are also included, the derivation of $Var(\widehat{\omega})$ needs to be modified. Let us assume that all true mediators are included and they represent only π fraction of the total selected mediators (for which $\widehat{\gamma}_i$ has a P-value $< p_T$). Note that as the P-value selection threshold gets milder this proportion (π) decreases. Using the Wick's formula [https://www.unige.ch/~vilmart/paper_isserlis.pdf], we obtain $E[(\gamma_i \cdot \delta_i)^2] = \pi \cdot (\sigma_\gamma^2 \cdot \sigma_\delta^2 + 2 \cdot \sigma_{\gamma,\delta}^2)$. Thus, the variance can be written as

$$Var(\omega) \;=\; k \cdot \left(\pi \cdot (\sigma_\gamma^2 \cdot \sigma_\delta^2 + 2 \cdot \sigma_{\gamma,\delta}^2) - \pi^2 \cdot \sigma_{\gamma,\delta}^2\right)$$

Let $p_1 = k \cdot \pi$, i.e. the number of selected non-null mediators. Therefore, $\omega = p_1 \cdot \sigma_{\gamma,\delta}$. Substituting this into the above equation we get

$$Var(\omega) \;=\; p_1 \cdot \sigma_\gamma^2 \cdot \sigma_\delta^2 + p_1 \cdot \sigma_{\gamma,\delta}^2 \cdot (2 - \pi)$$

Assuming that all non-null mediators have already been picked up at threshold p_T, π can be expressed as

$$\pi \;\approx\; \frac{p_1}{p_T \cdot k \cdot (1 - p_k) + k \cdot p_k}$$

Substituting this into the variance equation we have

$$Var(\omega) \;=\; p_1 \cdot \sigma_\gamma^2 \cdot \sigma_\delta^2 + p_1 \cdot \sigma_{\gamma,\delta}^2 \cdot \left(2 - \frac{p_1}{p_T \cdot k \cdot (1 - p_k) + k \cdot p_k}\right)$$

Finally,

$$\begin{aligned} E\left[\frac{\widehat{\omega}}{\widehat{\omega} + \widehat{\alpha}}\right] &\approx \frac{\omega}{\omega + \alpha} - \frac{\alpha}{(\omega + \alpha)^3} \cdot Var(\widehat{\omega}) \\ &\approx \frac{\omega}{\omega + \alpha} - \frac{\alpha}{(\omega + \alpha)^3} \cdot \left[p_1 \cdot \sigma_\gamma^2 \cdot \sigma_\delta^2 + p_1 \cdot \sigma_{\gamma,\delta}^2 \cdot \left(2 - \frac{p_1}{p_T \cdot k \cdot (1 - p_k) + k \cdot p_k}\right)\right] \end{aligned}$$

The shape of the bias as a function of the threshold p_T is of the form shown in Figure S12.

In summary, these two behaviors explain why introducing milder mediator filtering (based on $X \to M$ or similarly $M \to Y$) leads first to an increase in MP estimates, then starts to decrease. These trends and the tipping point depends on many parameters and it is unknown for real data applications.

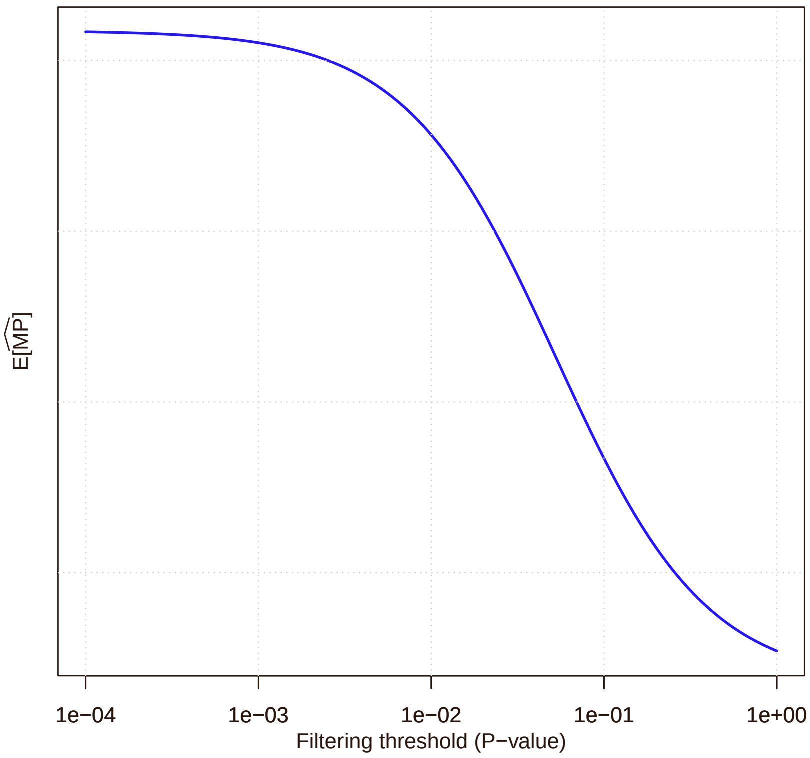

Fig. S12 | (I-)LiMA MP estimation bias as a function of the mediator filtering threshold (p_T).

References

[1] Sun, B. B., Maranville, J. C., Peters, J. E., Stacey, D., Staley, J. R., Blackshaw, J., Burgess, S., Jiang, T., Paige, E., Surendran, P., et al. (2018). Genomic atlas of the human plasma proteome. Nature *558*, 73–79.

[2] Zhu, J., Burgess, S., and Grant, A. J. (2022). Bias in multivariable mendelian randomization studies due to measurement error on exposures.

[3] Shin, S.-Y., Fauman, E. B., Petersen, A.-K., Krumsiek, J., Santos, R., Huang, J., Arnold, M., Erte, I., Forgetta, V., Yang, T.-P., et al. (2014). An atlas of genetic influences on human blood metabolites. Nat. Genet. *46*, 543–550.

[4] Võsa, U., Claringbould, A., Westra, H.-J., Bonder, M. J., Deelen, P., Zeng, B., Kirsten, H., Saha, A., Kreuzhuber, R., Yazar, S., et al. (2021). Large-scale cis- and trans-eQTL analyses identify thousands of genetic loci and polygenic scores that regulate blood gene expression. Nat. Genet. *53*, 1300–1310.

[5] Nadarajah, S. and Pogány, T. K. (2016). On the distribution of the product of correlated normal random variables. C. R. Math. *354*, 201–204.

[6] Alman, J. and Williams, V. V. (2021). A refined laser method and faster matrix multiplication. In Proceedings of the 2021 ACM-SIAM Symposium on Discrete Algorithms (SODA) pp. 522–539. (Society for Industrial and Applied Mathematics).
